# The *Bacillus subtilis* Conjugative Plasmid pLS20 Encodes Two Ribbon-Helix-Helix Type Auxiliary Relaxosome Proteins That Are Essential for Conjugation

**DOI:** 10.3389/fmicb.2017.02138

**Published:** 2017-11-03

**Authors:** Andrés Miguel-Arribas, Jian-An Hao, Juan R. Luque-Ortega, Gayetri Ramachandran, Jorge Val-Calvo, César Gago-Córdoba, Daniel González-Álvarez, David Abia, Carlos Alfonso, Ling J. Wu, Wilfried J. J. Meijer

**Affiliations:** ^1^Department of Virology and Microbiology, Centro de Biología Molecular “Severo Ochoa” (CSIC-UAM), Instituto de Biología Molecular “Eladio Viñuela” (CSIC), Autonomous University of Madrid, Madrid, Spain; ^2^The Institute of Seawater Desalination and Multipurpose Utilization (SOA), Tianjin, China; ^3^Centro de Investigaciones Biológicas (CSIC), Madrid, Spain; ^4^Centre for Bacterial Cell Biology, Institute for Cell and Molecular Biosciences, Newcastle University, Newcastle Upon Tyne, United Kingdom

**Keywords:** conjugation, relaxosome, auxiliary protein, DNA binding protein, Ribbon-Helix-Helix, antibiotic resistance, Firmicutes, horizontal gene transfer

## Abstract

Bacterial conjugation is the process by which a conjugative element (CE) is transferred horizontally from a donor to a recipient cell via a connecting pore. One of the first steps in the conjugation process is the formation of a nucleoprotein complex at the origin of transfer (*oriT*), where one of the components of the nucleoprotein complex, the relaxase, introduces a site- and strand specific nick to initiate the transfer of a single DNA strand into the recipient cell. In most cases, the nucleoprotein complex involves, besides the relaxase, one or more additional proteins, named auxiliary proteins, which are encoded by the CE and/or the host. The conjugative plasmid pLS20 replicates in the Gram-positive Firmicute bacterium *Bacillus subtilis*. We have recently identified the relaxase gene and the *oriT* of pLS20, which are separated by a region of almost 1 kb. Here we show that this region contains two auxiliary genes that we name *aux1_LS20_* and *aux2_LS20_*, and which we show are essential for conjugation. Both Aux1_LS20_ and Aux2_LS20_ are predicted to contain a Ribbon-Helix-Helix DNA binding motif near their N-terminus. Analyses of the purified proteins show that Aux1_LS20_ and Aux2_LS20_ form tetramers and hexamers in solution, respectively, and that they both bind preferentially to *oriT_LS20_*, although with different characteristics and specificities. *In silico* analyses revealed that genes encoding homologs of Aux1_LS20_ and/or Aux2_LS20_ are located upstream of almost 400 relaxase genes of the Rel_LS20_ family (MOB_L_) of relaxases. Thus, Aux1_LS20_ and Aux2_LS20_ of pLS20 constitute the founding member of the first two families of auxiliary proteins described for CEs of Gram-positive origin.

## Introduction

Bacteria exchange genetic material at gross scale, even between distantly related species, via different routes collectively called horizontal gene transfer (HGT) (for review see, Ochman et al., 2000; Frost et al., 2005; Thomas and Nielsen, 2005; Boto, 2010). Horizontal exchange of DNA provides bacteria instantly with a new set of gene(s) and hence is an important driver for the rapid adaptation and evolution of bacteria. Among the genes that are spread by HGT are those responsible for antibiotic resistance (AR), which poses a serious and increasingly worrisome economic and health problem at a global scale. Three main mechanisms are responsible for HGT: transformation through natural competence, transduction via bacterial phage, and conjugation (Ochman et al., 2000; Frost et al., 2005; Thomas and Nielsen, 2005). Of these, conjugation appears to be the route that is predominantly responsible for spreading AR genes (Mazel and Davies, 1999; Waters, 1999; Norman et al., 2009; Davies and Davies, 2010). Conjugation is the process by which a conjugative element (CE) is transferred from a donor cell to a recipient cell through a dedicated transportation pore connecting both cells. CEs contain all the genes required for processing the DNA, establishing contact with the recipient cell, those encoding the structural proteins of the connecting pore as well as those for transporting the DNA. CEs can be integrated in a bacterial chromosome or be present on plasmids, which are named integrative and conjugative elements (ICEs) and conjugative plasmids, respectively. Due to the enormous numbers and density of microbes and the constant replenishment of bacteria upon the intake of food and liquids, the intestinal gut of humans and animals is a niche that is particularly apt for emerging, pooling, and spreading AR (Sommer et al., 2009, 2010; Forsberg et al., 2012; Penders et al., 2013).

Conjugative elements are commonly present in Gram-positive (G+) and Gram-negative (G-) bacteria and the basic concepts of the transfer process are conserved (Alvarez-Martinez and Christie, 2009; De la Cruz et al., 2010; Smillie et al., 2010; Goessweiner-Mohr et al., 2013). However, whereas in most systems conjugation involves the transfer of a single DNA strand (see below), DNA is transferred in its double-stranded form during conjugation in G+ mycelial *Streptomyces* bacteria (Goessweiner-Mohr et al., 2013; Thoma and Muth, 2016), which is not further considered here. Conjugation starts with a process named mating pair formation (Mpf) in which a donor cell recognizes and interacts with a suitable recipient cell. Probably, this triggers the signal for processing the DNA of the CE and subsequent transfer of one of its strands, named T-strand, into the recipient cell. The sophisticated, multi-component pore connecting the donor and the recipient cell is named transferosome, which is a type IV secretion system (T4SS). The enzyme responsible for initiating the generation of the T-strand is a relaxase, a phosphodiesterase, that cleaves the DNA in a strand- and site-specific manner at a specific position called the *nic* site, which is located within the origin of transfer region (*oriT*). Relaxase-mediated cleavage generates a hydroxyl group at 3′ end of the *nic* site which functions as a primer for DNA elongation; i.e., the relaxase initiates a rolling-circle type of DNA replication (also named DNA transfer replication [Dtr]). Upon nicking, the relaxase remains covalently attached to the 5′-end of the nicked T-strand which is then transferred, together with the attached T-strand, into the recipient cell. In most cases the active site residue that becomes covalently attached to the T-strand concerns a tyrosine. However, very recently it has been shown that relaxases of the MOB_V_ family employ a histidine instead of a tyrosine residue to nick the DNA (Pluta et al., 2017). Due to its crucial role in conjugation, relaxases have attained considerable attention and several of them have been characterized in detail at the biochemical, functional and structural levels. In some cases, for instance ICE*Bs1* of *Bacillus subtilis* and the broad host range conjugative plasmid pIP501, the relaxase is the only protein that is required for processing the DNA (Kopec et al., 2005; Lee and Grossman, 2007; Grohmann et al., 2016). However, in the majority of cases additional protein(s), encoded either by the CE or the host, bind to the *oriT* and are involved in processing of the DNA. The nucleoprotein complex at *oriT* formed by the relaxase and additional proteins is called the relaxosome, and the additional proteins are named auxiliary or accessory proteins. Although their name may suggest that they play secondary role(s) in the processing reaction, most if not all of the auxiliary proteins studied so far have been shown to be essential for conjugation.

Most conjugation studies are based on CE present in G- bacteria, with knowledge on conjugation-related aspects in G+ bacteria lagging far behind. This is especially the case for auxiliary proteins (see Discussion). In our laboratory we study the conjugative plasmid pLS20 which was originally isolated from the Gram+ Firmicute bacterium *B. subtilis* natto IFO3335 (Tanaka et al., 1977). This strain is used for the fermentation of soybeans to produce “natto,” a popular dish in South Asia, and hence it is conceivable that pLS20 or relatives play a role in the conjugation-mediated HGT in the gut of humans and animals. A derivative of pLS20 containing a chloramphenicol-resistance gene, pLS20cat, has been constructed (Itaya et al., 2006) and its sequence has been determined in our lab and in the lab of M. Itaya (Mitsuhiro Itaya, Keio University, Japan). All conjugation genes are located in one large operon spanning genes *28* till *74* according to our nomenclature (Singh et al., 2013). pLS20cat genes *25-27* are involved in regulating the expression of the conjugation genes (Singh et al., 2013; Ramachandran et al., 2014). Recently, we have identified and characterized the relaxase (gene *58*) and the *oriT* of pLS20cat, which we named Rel_LS20_ and *oriT_LS20_*, respectively (Ramachandran et al., 2017). Contrary to many other plasmids, the relaxase gene and *oriT* are located within its large conjugation operon, and Rel*_LS20_* turned out to be the founding member of a novel relaxase family containing >800 members.

Here, we addressed the question whether pLS20cat contains auxiliary relaxosome genes. We demonstrate that genes *56* and *57*, located in between the relaxase gene *rel_LS20_* and *oriT_LS20_* are two auxiliary genes that are essential for conjugation and denominated them as *aux1_LS20_* and *aux2_LS20_*, respectively. Both gene products were purified and biochemical analyses showed that one of them formed tetramers and the other hexamers in solution. We also show that the proteins bind to distinct DNA motifs present in *oriT_LS20_*. *In silico* analyses revealed that a large fraction of the relaxase genes coding for the MOB_L_ family of relaxases are preceded by genes encoding homologs of Aux1_LS20_ and/or Aux2_LS20_. The findings obtained for Aux1_LS20_ and Aux2_LS20_ are placed in perspective with other auxiliary proteins of CE present in G+ and G- organisms.

## Materials and Methods

### Bacterial Strains, Plasmids, Media and Oligonucleotides

*Escherichia coli* and *B. subtilis* strains were grown in Luria-Bertani (LB) liquid medium or on 1.5% LB agar plates. When appropriate, media were supplemented with the following antibiotics: ampicillin (100 μg/ml), erythromycin (1 and 150 μg/ml in *B. subtilis* and *E. coli*, respectively), chloramphenicol (5 μg/ml), spectinomycin (100 μg/ml), and kanamycin (10 and 30 μg/ml in *B. subtilis* and *E. coli*, respectively). *B. subtilis* strains used were isogenic with *B. subtilis* strain 168 and are listed in Supplementary Table S1. Plasmids and oligonucleotides used are listed in Supplementary Tables S2, S3, respectively. All oligonucleotides were purchased from Isogen Life Science, Netherlands.

### Transformation

*Escherichia coli* cells were transformed using standard methods (Sambrook et al., 1989). Preparation of competent *B. subtilis* cells and transformation were carried as described before (Bron et al., 1989). Transformants were selected on LB agar plates with appropriate antibiotics. pLS20cat encodes a protein, Rok_LS20_, that inhibits the development of competence by repressing *comK*, the key transcriptional activator of competence genes (Singh et al., 2012). Therefore, to manipulate genes on pLS20cat we prepared competent cells of a pLS20cat-harboring strain that contains a chromosomal P_xyl_-*comK* fusion (PKS56) using a standard protocol (Singh et al., 2012).

### Construction of Plasmids and Strains

The correctness of sequences of all cloned PCR fragments was confirmed by sequence analysis. Amplification by PCR of pLS20cat regions was performed using as template total DNA isolated from pLS20cat harboring strain PKS11. Details regarding the construction of integration vectors based on plasmids pDR110 (*amyE* integration vector with IPTG-inducible P*_spank_* promoter) or pAX01 (*lacA* integration vector with xylose-inducible P*_xyl_* promoter) are given in Supplementary Table S2. In summary, gene *56* was cloned under the control of the P*_xyl_* promoter or the P*_spank_* promoter. In addition, genes *56-57-58*, genes *57-58*, or gene *58* were cloned behind the P*_spank_* promoter. Plasmid DNA of the constructed pAXO1 and pDR110 derivatives was isolated from *E. coli* cells and then used to transform competent *B. subtilis* cells. Double-crossover integration into the chromosome was checked by PCR in the case of the pAXO1-derivatives. When pDR110 derivatives were used to transform competent *B. subtilis* cells, double cross over integration was tested by the loss of amylase activity. The pLS20cat genes 58 (*rel_LS20_*), 57 (*aux1_LS20_*) and 56 (*aux2_LS20_*) were cloned in the *E. coli* expression vector pET28b+ to generate fusion genes containing a C-terminal *his_(6)_* extension. Details regarding these cloning strategies are given in Supplementary Table S2. The resulting derivatives of pET28b+ were constructed using *E. coli* strain XL1-Blue. Once verified its correctness, the plasmids were transformed into *E. coli* strain BL21(DE3).

### Conjugation Assays

Conjugation was carried out in liquid medium as described previously (Singh et al., 2013). The effect of ectopic expression of a given gene placed under the control of the inducible P*_spank_* and/or P*_xyl_* promoter on conjugation was studied by adding the inducer (1 mM IPTG, 1% xylose) to prewarmed LB medium used to dilute overnight cultures of the donor cells.

### Analytical Ultracentrifugation Experiments

Sedimentation velocity (SV), sedimentation equilibrium (SE), and dynamic light scattering (DLS) assays and processing of the data, including estimations of molar masses of the relaxosome proteins from the hydrodynamic measurements, were carried out using the same conditions to those used before in the analysis of Rel_LS20_ (Ramachandran et al., 2017).

### Over Expression and Purification of Recombinant Rel_LS20_, Aux1_LS20_, and Aux2_LS20_ Containing a C-Terminal His_(6)_ Tag

Recombinant versions of Rel_LS20_, Aux1_LS20_, and Aux2_LS20_ were expressed and purified using similar protocols. In brief, *E. coli* BL21(DE3) cells containing plasmid pAND83 (*rel_LS20_His_(6)_*), or pHJA56 (*aux1_LS20_His_(6)_*), or pHJA57 (*aux2_LS20_His_(6)_*) were inoculated in fresh LB media complemented with 30 μg/ml kanamicin and grown at 37°C with shaking (200 rpm). At an OD_600_ of about 0.6, IPTG was added to a final concentration of 1 mM to induce the recombinant protein and growth was continued for 2 h. Next, cells were collected by centrifugation and processed as described before (Singh et al., 2012). The nickel-column purified proteins (>95% pure) were finally dialysed against buffer B (20 mM Tris-HCl pH 8.0, 1 mM EDTA, 500 mM NaCl, 10 mM MgCl_2_, 7 mM β-mercaptoethanol, 50% v/v glycerol) and stored in aliquots at -80°C. Bradford assay and OD_280_ determination were used to determine the protein concentrations.

### Gel Retardation Assays

Gel retardation assays were essentially carried out as described before (Singh et al., 2012). Thus, different DNA fragments were amplified by PCR using pLS20cat as template. The resulting PCR fragments were purified and 170 ng of DNA [200 or 362 bp] (with or without 220 ng of control DNA [176 bp]) were incubated on ice in binding buffer [20 mM Tris HCl pH 8, 1 mM EDTA, 5 mM MgCl_2_, 0.5 mM DTT, 100 mM KCl, 10% (v/v) glycerol, 0.05 mg ml^-1^ BSA] without or with purified Aux1_LS20_ or Aux2_LS20_ to a fixed final concentration of 90 nM (Supplementary Figure S3) or using twofold increasing concentrations ranging from 0.09 to 5.76 μM (**Figure 3**) in a total volume of 16 μl. The negative control, corresponding to bp numbers 63,774–63,950 of accession number NC_015148.1, has an AT-content that is very similar to the AT content of the *oriT* fragment (61.4 vs. 61.1%). This DNA corresponds to sequences located inside a gene (gene *24*), lowering the possibility that it harbors particular features for recruiting a transcriptional regulator or other DNA binding protein. In addition, it is predicted to lack a static bend. After careful mixing, samples were incubated for 20 min at 30°C, placed back on ice for 10 min, then loaded onto 2% agarose gel in 0.5XTBE. Electrophoresis was carried out in 0.5XTBE at 50 V at 4°C. Finally, the gel was stained with ethidium bromide, destained in 0.5XTBE and photographed with UV illumination.

### *In Silico* Analyses

#### Identification of Mob_L_ Members

Rel_LS20_ was used as a query sequence to execute a psi-blast (version 2.6.1+) search against the NCBI nr protein database (July, 2017), allowing up to 10 rounds of reiteration with an *e*-value threshold of 1e-15 (Altschul et al., 1997, 2005; Schaffer et al., 2001) producing 1445 hits. The program “USEARCH” (version v10.0.240_i86linux32) was then used to identify and remove redundant sequences showing 100% identity (Edgar, 2010), resulting in 1249 unique hits showing high similarity to Rel_LS20_.

#### Identification of Putative Auxiliary Proteins

Protein sequences of Aux1_LS20_ and Aux2_LS20_ were used as query against the NCBI nr protein database (July 2017) using psi-blast (version 2.6.1+), with an e-value threshold of 1e-6 and 1e-7, respectively, until no new hits were retrieved. The sequence identifiers obtained from psi-blast, were crossed with the sequence identifiers preceding the MOB_L_ family relaxase members, obtained from the nucleotide entries from they were translated.

#### Prediction of Secondary Structure for Aux1_LS20_ and Aux2_LS20_ Homologs

Corresponding sequences were submitted to the RaptorX property web server (Wang et al., 2016) and predictions for β-strands and α-helices along the sequences were plotted with “R”^1^) (R Core Team, 2017).

## Results

### Identification of Putative Relaxosome Genes of pLS20cat by *in Silico* Analysis

**FIGURE 1:**
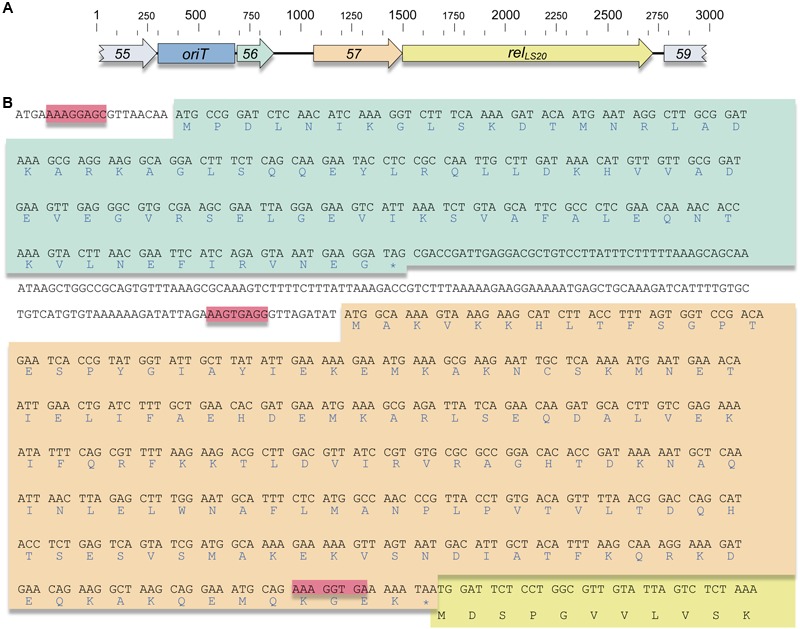
The pLS20cat relaxosome region. **(A)** Genetic organization of the pLS20cat genes *55–59*. The genes, their sizes and orientation of transcription are indicated with arrows. Genes *55* and *59* are colored gray. Genes *56, 57*, and *58* (*rel_LS20_*) are colored green, orange, and yellow, respectively. The same color code is used in “**B**,” as well as in **Figure 4** (see below). The 362 bp *oriT_LS20_* region is indicated with a blue box labeled *oriT*. Base pair numbering is given on the top. **(B)** DNA sequence of genes *56* and *57* and their deduced protein sequences. Stop codons are indicated with an asterisk and likely Ribosomal Binding sites (RBS) are highlighted with a red box. Note that genes *57* and *rel_LS20_* are translationally coupled. Only the first 11 codons of the *rel_LS20_* gene are given.

Recently, we have shown that pLS20cat gene *58* is essential for conjugation and that it encodes the relaxase, which we named Rel_LS20_ (Ramachandran et al., 2017). In these studies we also identified the *nic* site of Rel_LS20_ and delineated the functional *oriT*, named *oriT_LS20_*, to a region of 362 bp. Remarkably, *oriT_LS20_* and *rel_LS20_* are separated by a region of 865 bp, which has been annotated to contain two relatively small putative genes, designated genes *56* and *57* (Singh et al., 2013, see **Figure 1** for a schematic view of this region). Often, but not always, conjugative plasmid-located relaxase genes are accompanied by small auxiliary relaxosome genes that generally are located upstream of the relaxase gene. This prompted us to investigate whether genes *56* and *57* might encode auxiliary relaxosome genes of pLS20cat. *In silico* analyses of pLS20cat genes *56* and *57* show that, firstly, *rel_LS20_* is translationally coupled to the preceding gene *57* [i.e., the stop (TAA) and start codon (ATG) of genes *57* and *rel_LS20_*, respectively, overlap; see **Figure 1**], and only a small intergenic region of 183 bp separates gene *57* from its preceding gene *56*. Second, gene *56* and *57* are both small genes (79 and 147 codons, respectively). And third, the proteins encoded by these genes are both putative DNA binding proteins predicted to contain a Ribbon-Helix-Helix (RHH) motif in their N-terminal regions. An overview of the secondary structure prediction of both proteins and their homology with CopG, a paradigm of RHH DNA binding protein (Gomis-Ruth et al., 1998; Del Solar et al., 2002), is shown in Supplementary Figure S1. This figure shows that both Aux1_LS20_ and Aux2_LS20_ contain several lysine and arginine residues near the end of their predicted helix 1 and beginning of helix 2. The corresponding region in known RHH structures has been shown to be close to the phosphate backbone of the DNA (for example see, Schildbach et al., 1999). In summary, *in silico* analyses suggested that the two small genes *56* and *57* preceding the relaxase gene *rel_LS20_* may encode auxiliary relaxosome proteins.

### pLS20cat Genes *56* and *57* Are Essential for Conjugation

Previously, we engineered a derivative of pLS20cat, pLS20Δ56-58, in which the putative genes *56-57* together with the relaxase gene *rel_LS20_ (*gene *58)* have been deleted, and demonstrated that this plasmid was deficient in conjugation. Conjugation of pLS20Δ56-58 was restored when all three genes (*56-58*), were ectopically expressed from the IPTG-inducible P*_spank_* promoter at the chromosomal *amyE* locus, but not in the absence of gene *58*, showing that Rel_LS20_ was essential for conjugation (Ramachandran et al., 2017). We used a similar approach to study whether genes *56* and/or *57* were essential for conjugation. Thus, we constructed strain GR153, which harbors pLS20Δ56-58 and also contains *rel_LS20_* (gene *58*), but not *56* and *57*, under the control of the P*_spank_* promoter at the *amyE* locus. We then employed this strain as donor to determine the conjugation efficiencies using a standard protocol (see Materials and Methods). Strains PKS11, GR149 and GR150 were included as controls. As shown in **Table 1**, the efficiency of conjugation observed for the wild type plasmid pLS20cat was in the range of 10^-3^, which is similar to values reported previously under similar conditions (Singh et al., 2013; Ramachandran et al., 2014, 2017). As reported before (Ramachandran et al., 2017), conjugation was observed for pLS20Δ56-58 only when genes *56-58* were expressed from the chromosome (**Table 1**, strain GR149 and GR150). Importantly, no transconjugants were obtained when strain GR153 (*amyE*::P*_spank_*-*rel_LS20_*, pLS20Δ56-58) was used as donor in conjugation experiments, regardless of whether they were grown in the presence or absence of IPTG. These results showed that pLS20cat gene *56* and/or *57* are necessary for conjugation.

**Table 1:** pLS20cat genes *56* and *57* are required for conjugation.

Strain	Genotype	Plasmid	Inductor	Conjugation efficiency^∗^
PKS11	168	pLS20cat	-	7.9 × 10^-3^
GR149	168	pLS20Δ56-58	-	<10^-7^
GR150	168, *amyE*::P*_spank_ 56-58*	pLS20Δ56-58	-	<10^-7^
			+	3.2 × 10^-3^
GR153	168, *amyE*::P*_spank_ 58*	pLS20Δ56-58	-	<10^-7^
			+	<10^-7^
GR197	168, *amyE*::P*_spank_ 57-58*	pLS20Δ56-58	-	<10^-7^
			+	<10^-7^
GR200	168, *amyE*::P*_spank_ 58, lacA*::P*_xyl_-56*	pLS20Δ56-58	-	<10^-7^
			+	<10^-7^
GR225	168, *amyE*::P*_spank_* 57-*58, lacA*::P*_xyl_-56*	pLS20Δ56-58	-	<10^-7^
			+	5.8 × 10^-4^

*^∗^Conjugation efficiencies were calculated as transconjugants/donor, and correspond to the mean value of at least three independent experiments. When indicated, the inducer IPTG was added at a final concentration of 1 mM in the case of strains GR150 and GR197. In the case of GR200 and GR225, the final concentrations of the inducers was 1 mM (IPTG) and 1% (xylose).*

We next tested whether only one or both genes were required for conjugation. For this, we constructed the pLS20Δ56-58-harboring strains GR197 and GR200 in which *rel_LS20_* together with either gene *57* (strain GR197) or gene *56* (strain GR200) could be induced from the bacterial genome. When used as donor, no transconjugants were obtained for each strain regardless whether they were grown in the absence or presence of the inductor(s) (see **Table 1**), demonstrating that both genes are essential for conjugation.

In the above conjugation experiments, one or a combination of genes *56, 57, rel_LS20_* was complemented by expressing them from the IPTG-inducible P*_spank_* promoter for all the strains except for strain GR200. In this strain *rel_LS20_* is controlled by P*_spank_* at the *amyE* locus and gene *56* by the xylose-inducible P*_xyl_* promoter at the *lacA* locus. To rule out the possibility that transconjugants were not obtained for donor strain GR200 because the genes were expressed from different promoters at a different locus, we constructed strain GR225 in which gene *56* was placed under the control of the P*_xyl_* promoter at *lacA*, and genes *57* and *58* under the control of the P*_spank_* promoter at *amyE*. Transconjugants were obtained for this strain when cells were grown in the presence of both inducers (**Table 1**), demonstrating that the gene products expressed from the two different promoters and chromosomal loci were all functional. These results demonstrate therefore that besides *rel_LS20_* genes *56* and *57* are also required for conjugation. Taking into account these results, together with the structural organization of these genes with respect to *rel_LS20_* and *oriT_LS20_*, the *in silico* analyses presented above, and additional evidence presented below, we conclude that pLS20cat gene *56* and *57* encode auxiliary relaxosome proteins which we name Aux1_LS20_ and Aux2_LS20_, respectively.

### *In Vitro* Analysis of the Relaxosome Proteins Aux1_LS20_ and Aux2_LS20_, and Rel_LS20_

#### Oligomerization State Determined by Analytical Ultracentrifugation and DLS Techniques

**FIGURE 2:**
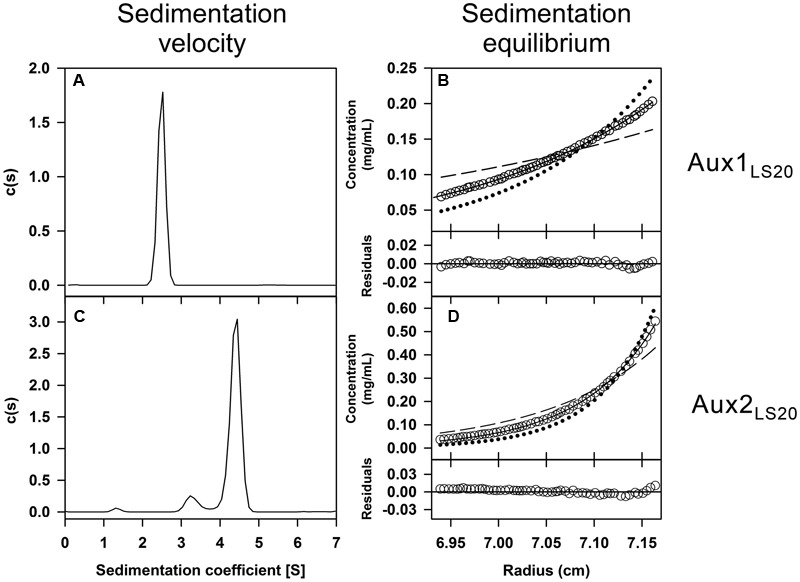
Determination of the oligomerization state of the relaxosome proteins Aux1_LS20_ and Aux2_LS20_. Purified proteins in solution at 12 μM were studied by sedimentation velocity (SV) and sedimentation equilibrium (SE). Plots **(A**,**C)** are representations of the sedimentation coefficient distribution c(s) profiles obtained by SV and correspond to Aux1_LS20_ and Aux2_LS20_, respectively. Concentration gradients obtained by SE assays: **(B)** Data obtained with Aux1_LS20_ (empty circles) and best-fit analysis assuming a protein tetramer (black line), dimer (dashed line) and hexamer (dotted line) species model; **(D)** Data collected with Aux2_LS20_ (empty circles) and best-fitting assuming a protein hexamer (black line), tetramer (dashed line) and octamer (dotted line) species model. Lower part in the SE plots represents the difference between experimental data and estimated values for the best fit to a single species model (residuals).

To characterize the auxiliary relaxosome proteins *in vitro*, we purified Aux1_LS20_ (Mw 10,601 Da) and Aux2_LS20_ (Mw 18,605 Da) from *E. coli*, each fused to a His_(6)_ tag at its C-terminus. We first determined the oligomerization state of the proteins, and also investigated putative interactions among them and with Rel_LS20_, using two complementary analytical ultracentrifugation approaches, i.e., SV and SE (**Figures 2A–D**), together with DLS experiments using the same experimental conditions.

Sedimentation profiles obtained by SV assays showed Aux1_LS20_ as a single species with an experimental sedimentation coefficient of 2.5 S (*s*_20,w_ = 2.9 S) compatible with a moderately elongated tetrameric form of the protein (*f*/*f*_0_ = 1.5) (**Figure 2A**). Subsequent analysis of Aux1_LS20_ gave a *D*-value of 52.5 ± 0.3 μm^2^/s. The obtained *S-* and *D*-values, once introduced in the Svedberg equation, yielded an apparent molar mass of 46,290 Da. SE data, best-fit analysis to single species model gave an average molecular mass of 42,200 Da ± 300 Da, confirming that Aux1_LS20_ is a tetramer in solution (**Figure 2B**).

In the case of Aux2_LS20_, analysis of the sedimenting boundaries showed a sedimentation profile with a main peak corresponding to 90.0% of the total proteins at 4.4 S (*s*_20,w_ = 5.1 S), together with a second peak at 3.3 S (*s*_20,w_ = 3.8 S) encompassing 7% of the sample (**Figure 2C**). The *S*-value of the main peak is compatible with the theoretical behavior of a spherical Aux2_LS20_ tetramer (*f*/*f*_0_ = 1.2), as well as with a moderately elongated hexamer (*f*/*f*_0_ = 1.6). DLS analysis of Aux2_LS20_ yielded a *D* of 38.2 ± 1.0 μm^2^/s, which combined with the obtained *S*-value of 4.4 in the Svedberg formula resulted in an apparent molar mass of 113,400 Da that is very close to the molecular mass of Aux2_LS20_ hexamers (111,630 Da). SE experiments were decisive for establishing the oligomerization state of Aux2_LS20_, as the best fit of the SE data gave an average molecular mass of 111,300 ± 1,200 Da, unequivocally demonstrating that Aux2_LS20_ forms hexamers in solution (**Figure 2D**). In summary, the outcome of three complementary experimental approaches showed that Aux1_LS20_ and Aux2_LS20_ form tetramer and hexamers in solution, respectively.

Previously, we determined that purified Rel_LS20_ forms monomers in solution (Ramachandran et al., 2017). To study possible interactions between the relaxosome proteins in solution we used combinations of Aux1_LS20_, Aux2_LS20_ and Rel_LS20_ and subjected these to SV experiments (Supplementary Figure S2). No additional peaks with increased *S*-values reflecting new protein hetero-complexes were obtained in any of the combinations tested implying that the relaxosome proteins of pLS20cat do not interact in solution, at least not under the conditions tested.

#### Aux1_LS20_ and Aux2_LS20_ Bind Specifically to *oriT_LS20_*

**FIGURE 3:**
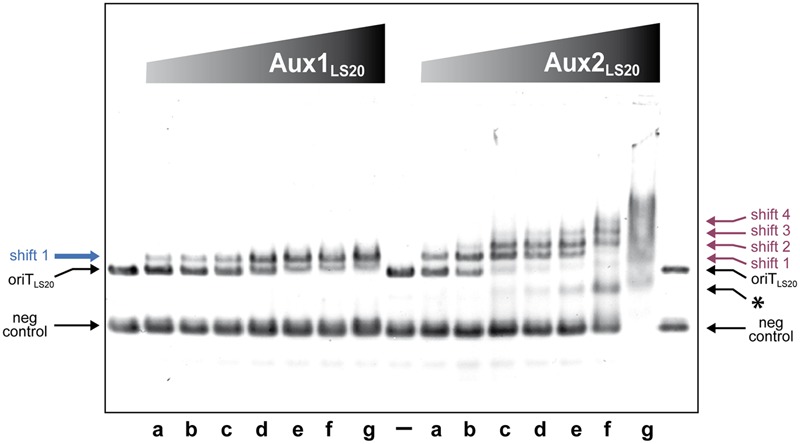
Auxiliary protein Aux1_LS20_ and Aux2_LS20_ are DNA binding proteins that preferentially bind to *oriT_LS20_*. DNA binding activity of Aux1_LS20_ and Aux2_LS20_ was analyzed by EMSA. Each lane contained two DNA fragments; one corresponding to *oriT_LS20_* (OriT_LS20_, 362 bp, 170 ng), and a control fragment (neg control, 176 bp, 220 ng, corresponding to pLS20 positions 63,774–63,950 of accession number NC_015148.1). –, loaded without protein. Increasing concentrations of Aux1_LS20_ or Aux2_LS20_ were prepared using twofold dilution steps, and ranged from 0.09 (lanes “a”) to 5.76 μM (lanes “g”). Positions of unbound control DNA (neg control) and *oriT_LS20_* (oriT_LS20_) are indicated. The single shifted *oriT_LS20_* species observed for Aux1_LS20_ is indicated on the left with a blue arrow. The different shifted *oriT_LS20_* species observed for Aux2_LS20_ are indicated on the right with purple arrows, and the shifted species of the control DNA fragment is indicated with an asterisk.

Electrophoretic Mobility Shift Assays (EMSA) were performed to study the DNA binding properties of Aux1_LS20_ and Aux2_LS20_. The results presented in **Figure 3** show that both auxiliary proteins bound DNA, and that both bound preferentially to *oriT_LS20_*. Nevertheless, there were distinct differences in binding characteristics between the two proteins. The addition of Aux1_LS20_ resulted in the appearance of only one retarded species of *oriT_LS20_*, and even at the highest concentration tested Aux1_LS20_ did not bind to the negative control DNA (**Figure 3**, left panel). One retarded *oriT_LS20_* species was also observed for Aux2_LS20_ at low concentrations. However, higher Aux2_LS20_ concentrations resulted in the appearance of additional shifted species of *oriT_LS20_*. In addition, at higher concentrations Aux2_LS20_ bound also to the negative control DNA, and at the highest concentration tested a smear of retarded species was observed (**Figure 3**, right panel). These results show that both proteins bind preferentially to *oriT_LS20_*, but Aux1_LS20_ appears to bind *oriT_LS20_* with a higher specificity than Aux2_LS20_.

To delineate further the binding sites of Aux1_LS20_ and Aux2_LS20_ we generated thirteen overlapping 200 bp DNA fragments (F21–F33) covering the *oriT_LS20_* region with a sliding window of 25 bp, and used them in EMSA. The results presented in Supplementary Figure S3 show that Aux1_LS20_ bound to fragments F22-F29, which share the 25 bp sequence 5′-CAAATAAATCTGGTACCACGAAAAA-3′ located in the 5′ half of *oriT_LS20_*. This sequence contains the inverted repeat 5′-TGGTACCA-3′, which could be the binding site of Aux1_LS20_. In the case of Aux2_LS20_ retarded species of *oriT_LS20_* with strong and weak intensity were observed for fragments F21–F25 and F26–F28, respectively. No shifts were observed for fragments F29–F33 at the protein concentration used. This shows that Aux2_LS20_ binds the 5′ half region of *oriT_LS20_* upstream of Aux1_LS20_. The sequence motif 5′-TGTGCAT-3′ is present three times in a directed repeated orientation in the 5′ half of *oriT_LS20_*. While fragments F21–F25 each contain the three 5′-TGTGCAT-3′ motifs, fragment F26 contains only two, and the motif is present only once on fragments F27 and F28. This suggests that the motif 5′-TGTGCAT-3′ may be the preferred binding site for Aux2_LS20_. It is worth mentioning that two of the 5′-TGTGCAT-3′ motifs are embedded within a larger motif (5′-TTTATGTGCATT-3′).

### Over 400 Members of the MOB_L_ Family of Relaxase Genes Contain Upstream Genes Encoding Homologs of Aux1_LS20_ and/or Aux2_LS20_

Previously, we reported that the pLS20cat-encoded Rel_LS20_ constitutes the founding member of a novel, large family of relaxases that we named MOB_L_, which contained 817 members that were almost exclusively encoded in bacteria belonging to the phylum Firmicutes (Ramachandran et al., 2017). We wanted to know whether other MOB_L_ relaxase genes were also preceded by genes encoding putative homologs of Aux1_LS20_ and/or Aux2_LS20_. To study this we first determined the current number of MOB_L_ relaxase genes, applying the same method as that used in our previous study; i.e., we performed a psi-blastp search of the NCBI nr database using Rel_LS20_ as a query. After removing redundant sequences this search now resulted in 1,453 hits that showed high similarity with Rel_LS20_ (threshold value *P* = 1e-15). Next, the corresponding DNA accession number of each identified MOB_L_ relaxase was retrieved, which was subsequently used to generate a database that contains the accession number of each MOB_L_ member together with that of the protein encoded by the gene located upstream and downstream of the relaxase gene. We then performed the same procedures for Aux1_LS20_ and Aux2_LS20_; i.e., we identified proteins sharing a high level of similarity with Aux1_LS20_ and Aux2_LS20_ and generated databases that contained these accession numbers together with those of the proteins encoded by the flanking genes. Finally, the three databases were crossed to identify those MOB_L_ members that are preceded by a gene encoding a putative homolog of Aux1_LS20_ and/or Aux2_LS20_. This approach revealed 387 MOB_L_ relaxase genes that were preceded by a gene encoding a putative Aux2_LS20_ homolog; and of these 87 contained an additional Aux1_LS20_ homolog encoding gene upstream. Without exception, the identified MOB_L_ relaxase genes having upstream gene(s) encoding putative homologs of Aux1_LS20_ and/or Aux2_LS20_ are all present in bacteria belonging to the phylum Firmicutes. Although stringent settings were used to identify proteins sharing high similarity with Aux1_LS20_ or Aux2_LS20_, this does not automatically imply that the identified proteins will contain a Ribbon-Helix-Helix motif in their N-terminal region, which is a characteristic feature of both Aux1_LS20_ and Aux2_LS20_ (see above). We therefore carried out secondary structure prediction for all the putative Aux1_LS20_ and Aux2_LS20_ homologs identified (see Materials and Methods). The results of these analyses, which are presented in Supplementary Table S4, show that 86 of the 87 (98.9%), and 384 of the 387 (99.2%) putative homologs of Aux1_LS20_ and Aux2_LS20_, respectively, contain a typical Ribbon-Helix-Helix signature in their N-terminal region, and thereby support the view that they are auxiliary proteins of the corresponding relaxase. In summary, these analyses provide compelling evidence that almost 400 MOB_L_ relaxase genes are preceded by a gene encoding an Aux2_LS20_ homolog, and that in 87 of these cases this putative auxiliary gene is preceded by another auxiliary gene encoding an Aux1_LS20_ homolog. Consequently, pLS20 encoded Aux1_LS20_ and Aux2_LS20_ are the founding members of two families of Ribbon-Helix-Helix type auxiliary proteins that are encoded by Firmicutes bacteria.

## Discussion

In this study we have demonstrated that the pLS20cat genes *56* (*aux1_LS20_*) and *57* (*aux2_LS20_*) encode the auxiliary relaxosome proteins of pLS20cat. Combined with our previously published results (Ramachandran et al., 2017), we have identified the relaxosome module of pLS20cat that includes *oriT_LS20_* and the downstream genes *aux1_LS20_, aux2_LS20_*, and *rel_LS20_*. This module is embedded within the large conjugation operon of pLS20cat (Singh et al., 2013). In addition, we have provided strong evidence that Aux1_LS20_ and Aux2_LS20_ constitute the founding member of corresponding families of Ribbon-Helix-Helix type auxiliary proteins whose genes precede a large fraction of the MOB_L_ type relaxase genes. Thereby, our results provide a better understanding of the relaxosome components present on Gram+ mobile elements in general and particularly those belonging to the phylum Firmicutes.

**FIGURE 4:**
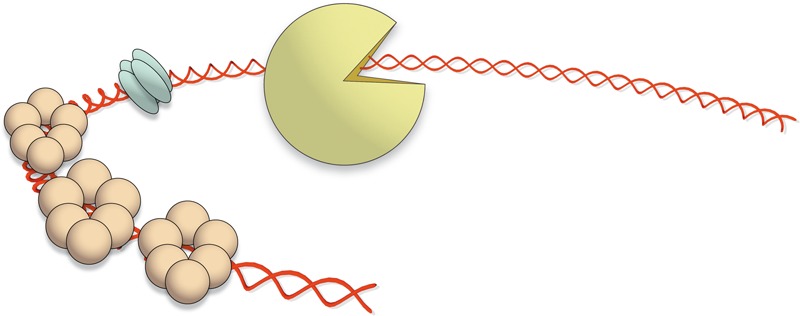
Schematic model of the nucleoprotein complex formed at *oriT_LS20_*. DNA is shown as a red double helix. The tetrameric Aux1_LS20_ unit, the hexameric Aux2_LS20_ units and the monomeric Rel_LS20_ are depicted as green, orange, and yellow cartoons, respectively. It is well possible that binding of one or more of the auxiliary proteins to *oriT_LS20_* alters the topology of the DNA. This is not taken into account in this simplified model.

The results presented here, together with those obtained previously (Ramachandran et al., 2017), show that *aux1_LS20_* and *aux2_LS20_* encode trans-acting proteins that are essential for conjugation. We also showed that Aux1_LS20_ and Aux2_LS20_ form tetramers and hexamers in solution, respectively, and we detected no interaction between the three pLS20 relaxosome proteins under the conditions tested. We cannot exclude the possibility that they interact when they form a nucleoprotein complex at *oriT_LS20_*. Aux1_LS20_ bound with high specificity to a region of 25 bp located about 100 bp upstream of the *nic* site that contains the inverted repeated sequence 5′-TGGTACCA-3′. The preferred binding site of Aux2_LS20_ resulted to be a 140 bp fragment located at the 5′ half of *oriT_LS20_* and that contains three times the sequence 5′-TGTGCAT-3′. In our previous study (Ramachandran et al., 2017), we showed that a derivative of *oriT_LS20_* that includes the *nic* site and the binding site for Aux1_LS20_, but lacks the 5′-located 100 bp containing two of the three 5′-TGTGCAT-3′ motifs was not functional *in vivo*. The topology of DNA can have a large effect on the binding characteristics of DNA binding proteins and which in turn may affect their function (Gimenes et al., 2008; Fogg et al., 2012). The *oriT* regions of several conjugative plasmids contain an intrinsic bend that is thought to be important for optimal binding and functionality of the relaxosome proteins (for review see, De la Cruz et al., 2010). We have demonstrated that the *oriT_LS20_* region is also intrinsically bent, and that the bend is located in the 5′ half of oriT*_LS20_* (Ramachandran et al., 2017), which we show here corresponds to the region where Aux1_LS20_ and Aux2_LS20_ (preferentially) bind. When we combine the results obtained here and in our previous study a picture emerges that is schematically presented in **Figure 4**. Aux1_LS20_ and Aux2_LS20_ bind to the left half of *oriT_LS20_* that is intrinsically bent and we envisage that the formation of this nucleoprotein complex contributes to optimal functioning of Rel_LS20_. In other systems, auxiliary proteins have been described to stimulate relaxase-mediated nicking at *oriT* by recruiting the relaxase to *oriT*, probably by facilitating the relaxase to access the *nic* site, and/or by acting as molecular wedges to melt double-stranded DNA (reviewed in, Alvarez-Martinez and Christie, 2009). Thus, it is conceivable that the auxiliary proteins of pLS20 fulfill similar function(s).

Most of our knowledge on auxiliary proteins is related to those encoded by conjugative plasmids replicating in G- bacteria; in particular, the auxiliary proteins of F and related plasmids have been studied in detail at the functional, biochemical and structural levels (for review see, Alvarez-Martinez and Christie, 2009; De la Cruz et al., 2010; Wong et al., 2012). Upon binding, TraY and TraM of plasmid F bent the DNA and therewith play important roles in organizing the relaxosome complex at *oriT* and influencing the nicking reaction of the relaxase. In addition, they both play a role in gene expression by regulating the activity of their own promoters. TraM also has a key role in delivering the relaxosome to the conjugative pore by interacting with its cognate T4CP (Wong et al., 2011; Peng et al., 2014). Future studies are needed to determine whether the auxiliary proteins of pLS20 fulfill similar functions to those of F, although it is doubtful that Aux1_LS20_ and Aux2_LS20_ play a role in gene regulation due to the different genetic organization. In the case of F, the monocistronic *traM* gene is located directly downstream of its *oriT*. *TraM* is followed by another monocistronic gene, *traJ*, which in turn is followed by a large multicistronic operon in which *traY* is the first gene (Zatyka and Thomas, 1998). In the case of pLS20, though, the relaxosome genes are embedded within the large conjugation operon and are under the control of the main conjugation promoter P_c_ that is located almost 26 kbp upstream of *aux1_LS20_* (Singh et al., 2013; Ramachandran et al., 2014). At present, we cannot fully exclude the possibility that the relaxosome genes of pLS20cat are controlled by an additional promoter that is regulated by Aux1_LS20_ or Aux2_LS20_. RNAseq data showed, however, that repression of the main conjugation promoter results in silencing of the relaxosome genes, as well as other genes in the conjugation operon of pLS20cat (Singh et al., 2013).

Far less is known about auxiliary proteins encoded by conjugative plasmids of Gram+ origin. The monomeric Helix-Turn-Helix protein TraN of the *Enterococcus faecalis* conjugative plasmid pIP501 binds to its *oriT* region, which suggested that it might be an auxiliary protein of pIP501. However, recent results revealed that *traN* is not essential for conjugation, and it is now believed that it may be a repressor of conjugation by regulating either the expression of the conjugation operon or activity of the relaxase TraA (Goessweiner-Mohr et al., 2014; Grohmann et al., 2016).

The auxiliary proteins PcfF encoded by the *E.nterococcus faecalis* plasmid pCF10, and LtrF of *Lactococcus lactis* plasmid pRS01 share 47% sequence identity. As far as we know, these are the only auxiliary proteins encoded by conjugative plasmids of Gram+ origin that have been studied in some detail (Chen et al., 2007, 2008). The *pcfF* and *ltrF* genes are essential for conjugation and purified PcfF and LtrF bind their cognate *oriT*s. Moreover, evidence supports a model in which PcfF recruits the relaxase PcfG to *oriT*, and that PcfF, probably in conjunction with the relaxase PcfG, interacts with its cognate T4CP and hence plays an important role in delivering the relaxosome to the conjugative pore.

Several auxiliary proteins of conjugative plasmids of Gram- origin are described to contain a RHH motif. These include, TraY and TraM of F plasmid, TrwA of R388, VirC2 of *Agrobacterium tumefaciens*, NikA of R64, TraJ of RP4, MobC of RSF1010, MbeC of ColE1, MobC of RA3 (Bowie and Sauer, 1990; Zhang and Meyer, 1997; Moncalian and De la Cruz, 2004; Ragonese et al., 2007; Yoshida et al., 2008; Lu et al., 2009; Varsaki et al., 2009; Godziszewska et al., 2016). For some of them structure-based mutational analyses have demonstrated the importance of the RHH motif in *oriT* binding as well as relaxase recruitment (Yoshida et al., 2008; Lu et al., 2009). Interestingly, Aux1_LS20_ and Aux2_LS20_ are also predicted to contain an RHH DNA-binding domain in their N-terminal region (Supplementary Figure S1). In addition, our *in silico* analyses predict that the auxiliary PcfF and LtrF proteins of Gram+ *E. faecalis* pCF10 and *L. lactis* pRS01 plasmids, respectively, also contain an RHH motif in their N-terminal region (our unpublished results). The presence of a likely RHH motif in Aux1_LS20_ and Aux2_LS20_ is therefore in line with the conclusion that they are auxiliary proteins. More importantly, the observation that the auxiliary proteins encoded by plasmids pLS20, pRS01, and pCF10, replicating in Gram+ bacteria, all contain a predicted RHH motif indicates that this is a conserved motif in auxiliary proteins encoded by CEs of both Gram- and Gram+ origin, and suggests that auxiliary proteins share a common ancestor. We have made use of this feature, combined with the genetic organization, to identify putative auxiliary genes located upstream of the MOB_L_ type relaxase genes that encode homologs of Aux1_LS20_ and Aux2_LS20_. This strategy resulted in the identification of about 400 and 90 genes encoding homologs of Aux2_LS20_ and Aux1_LS20_, respectively; 99.2% (Aux2_LS20_) and 98.9% (Aux1_LS20_) of these homologs were predicted to contain a Ribbon-Helix-Helix motif in their N-terminal region. These results reinforce therefore the view that an N-terminal Ribbon-Helix-Helix DNA binding motif is a characteristic feature of auxiliary relaxosome proteins. In addition, these data showed that Aux1_LS20_ and Aux2_LS20_ are the founding members of two families of auxiliary proteins whose genes are genetically linked to a MOB_L_ type relaxase gene. In summary, we have demonstrated that pLS20cat genes *56* (*aux1_LS20_*) and *57* (*aux2_LS20_*) encode the auxiliary proteins of pLS20 that are essential for conjugation, and that they form the founding members of families of auxiliary relaxosome proteins that are encoded in Firmicutes bacteria.

## Author Contributions

All authors listed have made substantial, direct experimental and/or intellectual contribution to the work. AM-A, J-AH, GR, CG-C, DG-A, and JV-C generated all plasmids and strains, purified proteins and executed all the experiments except the ultracentrifugation studies, which were performed by JL-O and CA. DA performed *in silico* analyses contributed to the general design and analyses of the results. LW and WM designed the experimental plan and were principally responsible for analyzing the results and writing the paper. WM supervised AM-A, J-AH, GR, CG-C, DG-A and JV-C.

## Conflict of Interest Statement

The authors declare that the research was conducted in the absence of any commercial or financial relationships that could be construed as a potential conflict of interest. The handling Editor declared a shared affiliation, though no other collaboration, with several of the authors JL-O and CA.

## Footnotes

1 https://www.R-project.org/
