# Supplementary material for: The Bacillus subtilis Conjugative Plasmid pLS20 Encodes Two Ribbon-Helix-Helix Type Auxiliary Relaxosome Proteins That Are Essential for Conjugation
Source: Front Microbiol. 2017 Nov 3;8:2138. doi: 10.3389/fmicb.2017.02138 (PMC5675868; doi:10.3389/fmicb.2017.02138)
Supplement: Supplementary file 1 [file Table_1.docx]

Supplementary Material

The *Bacillus subtilis* conjugative plasmid pLS20 encodes two ribbon-helix-helix type auxiliary relaxosome proteins that are essential for conjugation

Andrés Miguel-Arribas, Jian-An Hao, Juan Roman Luque-Ortega, Gayetri Ramachandran, Jorge Val-Calvo, César Gago-Córdoba, Daniel González-Álvarez, David Abia, Carlos Alfonso, Ling J. Wu and Wilfried J.J. Meijer*

*** Correspondence:** wmeijer@cbm.csic.es

**Supplemental Table 1**

| **Supplemental TableS1. Strains used** | | |
| --- | --- | --- |
| **Strains** | **genotype or description** | **Source or Reference** |
| *E. coli* |  |  |
| XL1-Blue | *end*A1 *gyr*A96(nal^R^) *thi*-1 *rec*A1 *rel*A1 *lac* *gln*V44 F'[ ::Tn10 *pro*AB^+^ *lac*I^q^ Δ(*lac*Z)M15] *hsd*R17 (r_K_^-^ m_K_^+^) | (Bullock et al., 1987) |
| BL21(DE3) | F^–^ *ompT* *gal* *dcm* *lon* *hsdS_B_*(*r_B_*^–^*m_B_*^–^) λ(DE3 [*lacI* *lacUV5*-*T7 gene 1* *ind1* *sam7* *nin5*]) [*malB*^+^]_K-12_(λ^S^) | laboratory stock |
| AZ37 | XL1-Blue strain harboring pET28b+ derivative pAND83 (containing *rel_LS20_His_(6)_*) | (Ramachandran et al., 2017) |
| AZ42 | BL21(DE3) strain harboring pET28b+ derivative pAND83 (containing *rel_LS20_His_(6)_*) | (Ramachandran et al., 2017) |
| BA50 | XL1-Blue strain harboring pET28b+ derivative pHJA56 (containing *aux1_LS20_His_(6)_*) | This work |
| BA54 | BL21(DE3) strain harboring pET28b+ derivative pHJA56 (containing *aux1_LS20_His_(6)_*) | This work |
| BA52 | XL1-Blue strain harboring pET28b+ derivative pHJA57 (containing *aux2_LS20_His_(6)_*) | This work |
| BA55 | BL21(DE3) strain harboring pET28b+ derivative pHJA56 (containing *aux2_LS20_His_(6)_*) | This work |
| *B. subtilis* |  |  |
| 168 (1A700) | *trpC2* | BGSC* |
| PKS11 | 168 harboring pLS20cat | (Singh et al., 2012) |
| GR126 | *trpC2*, *amyE*::P*_spank_*-56-58 (Spec) | (Ramachandran et al., 2017) |
| GR127 | *trpC2,* *amy*E::P*_spank_*-*rel_LS20_*, (Spec) | This work |
| GR149 | *trpC2,* pLS20Δ56-58 (Km, Cm) | (Ramachandran et al., 2017) |
| GR150 | *trpC2*, *amyE*::P*_spank_*-56-58 (Spec), pLS20Δ56-58 (Km, Cm) | (Ramachandran et al., 2017) |
| GR153 | *trpC2,* *amyE*::P*_spank_*-*rel_LS20_* (Spec), pLS20Δ56-58 (Km, Cm) | This work |
| GR193 | *trpC2,* *amy*E::P_spank_-*rel_LS20_* (Spec), lacA::P_xyl_-*56* (Em) | This work |
| GR197 | *trpC2,* *amy*E::P_spank_-*57*-*58* (Spec), pLS20Δ56-58 (Km, Cm) | This work |
| GR200 | *trpC2,* *amy*E::P_spank_-*rel_LS20_*, *lac*A::P_xyl_-*56* (Em), pLS20Δ56-58 (Km, Cm) | This work |
| GR206 | *trpC2,* *amy*E::P_spank_-*56-57* (Spec), pLS20Δ56-58 (Km, Cm) | This work |
| *, BGSC:  *Bacillus* Genetic Stock Center, Department of Bioch*em*istry, The Ohio State University, Columbus, OH, USA. (<http://www.bgsc.org/> | | |

**REFERENCES**

Bullock, W. O., Fernandez, J. M., and Short, J. M. (1987). XL1-blue: a high efficiency plasmid transforming *recA* *Escherichia coli* strain with Beta-galactosidase selection. *Biotechniques* 5, 376-379.

Ramachandran, G., Miguel-Arribas, A., Abia, D., Singh, P. K., Crespo, I., Gago-Cordoba, C. et al. (2017). Discovery of a new family of relaxases in Firmicutes bacteria. *PLoS Genet.* 13, e1006586.

Singh, P. K., Ramachandran, G., Duran-Alcalde, L., Alonso, C., Wu, L. J., and Meijer, W. J. (2012). Inhibition of Bacillus subtilis natural competence by a native, conjugative plasmid-encoded comK repressor protein. *Environ. Microbiol.* 14, 2812-2825.
