# Supplementary material for: The Bacillus subtilis Conjugative Plasmid pLS20 Encodes Two Ribbon-Helix-Helix Type Auxiliary Relaxosome Proteins That Are Essential for Conjugation
Source: Front Microbiol. 2017 Nov 3;8:2138. doi: 10.3389/fmicb.2017.02138 (PMC5675868; doi:10.3389/fmicb.2017.02138)
Supplement: Supplementary file 2 [file Table_2.docx]

Supplementary Material

The *Bacillus subtilis* conjugative plasmid pLS20 encodes two ribbon-helix-helix type auxiliary relaxosome proteins that are essential for conjugation

Andrés Miguel-Arribas, Jian An Hao, Juan Roman Luque-Ortega, Gayetri Ramachandran, Jorge Val-Calvo, César Gago-Córdoba, Daniel González-Álvarez, David Abia, Carlos Alfonso, Ling J. Wu and Wilfried J.J. Meijer*

*** Correspondence:** wmeijer@cbm.csic.es

| **Supplemental Table S2. Plasmids used** | | |
| --- | --- | --- |
| **Plasmid** | **Description** | **Reference or source** |
| pLS20cat | Native plasmid pLS20 labelled with Cm resistance cassette in the unique *Sal*I site | (Itaya et al., 2006) |
| pDR110 | *B. subtilis amyE* integration vector containing IPTG-inducible P*_spank_* promoter (Spec) | D. Rudner* |
| pAXO1 | *B. subtilis lacA* integration vector containing xylose-inducible P*_xyl_* promoter (Em) | (Hartl et al., 2001) |
| pLS20Δ56-58 | pLS20cat derivative in which genes *56-58* are replaced by kanamycin resistance gene of pBEST501 | (Ramachandran et al., 2017) |
| pET28b+ | Vector for expressing His-tagged labeled proteins in *E. coli* | Novagene, Madison, WI, USA |
| pGR23 | pDR110 derivative containing pLS20cat gene *58* (*rel_LS20_*) behind the IPTG-inducible P*_spank_* promoter. Gene *58* was amplified with primer set oGR45/oGR60. The resulting PCR fragment was digested with *Nhe*I and *Sph*I and cloned into the same sites of pDR110. | This work |
| pGR27 | pDR110 derivative containing pLS20cat gene *56 (aux1_LS20_)-57 (aux2_LS20_)- 58* (*rel_LS20_*) behind the IPTG-inducible P*_spank_* promoter. Genes *56-57-58* were amplified with primer set oGR43/oGR60. The resulting PCR fragment was digested with *Nhe*I and *Sph*I and cloned into the same sites of pDR110. | (Ramachandran et al., 2017) |
| pGR42 | pDR110 derivative containing pLS20cat gene *57 (aux2_LS20_)* and *58* (*rel_LS20_*) behind the IPTG-inducible P*_spank_* promoter. Genes *57-58* were amplified with primer set oGR26/oGR60. The resulting PCR fragment was digested with *Nhe*I and *Sph*I and cloned into the same sites of pDR110. | This work |
| pGR43 | pAX01 derivative containing pLS20cat gene *56* behind the xylose-inducible P*_xyl_* promoter. Gene *56* was amplified using primer set oGR133/oGR134. The resulting PCR fragment was digested with *Spe*I and *BamH*I and cloned into the same sites of pAX01. | This work |
| pAND83 | pET28b+ expression vector containing pLS20cat gene *58* (*rel_LS20_*) cloned in *Xba*I-*Sal*I sites to generate *rel_LS20_His_(6)_*. Gene *58* was amplified using primer set oWM001/oWM002. The resulting PCR fragment was digested with *Nco*I and *Sal*I and cloned into the same sites of pET28b+. | (Ramachandran et al., 2017) |
| pHJA56 | pET28b+ expression vector containing pLS20cat gene *56* (*aux1_LS20_*) cloned in *Xba*I-*Sal*I sites to generate *aux1_LS20_His_(6)_*. Contrary to *rel_LS20_* and *aux2_LS20_*, the second codon of *aux1_LS20_* does not start with a GC basepair and hence the *Nco*I site could not be used for cloning. To maintain the original sequence of *aux1_LS20_*, the DNA region comprising this gene was first amplified using the primer set oJH03/oJH05. The resulting PCR product was then used as template in a second PCR reaction using primer set oJH04/oJH05 to generate a region upstream of *aux1_LS20_* that is identical as in the pET28b+ vector up to the *Xba*I site. The PCR fragment of this second PCR reaction was digested with *Xba*I and *Sal*I and cloned into pET28b+ digested with the same enzymes. | This work |
| pHJA57 | pET28b+ expression vector containing pLS20cat gene *57* (*aux2_LS20_*) cloned in *Xba*I-*Sal*I sites to generate *aux2_LS20_His_(6)_* fusion gene. Gene *57* was amplified using primer set oJH01/oJH02. The resulting PCR fragment was digested with *Nco*I and *Sal*I and cloned into the same sites of pET28b+. | This work |
| *: D. Rudner, Department of Microbiology and Immunobiology, Harvard Medical School, 77 Avenue Louis Pasteur, Boston, MA 02115, USA | | |

**REFERENCES**

Hartl, B., Wehrl, W., Wiegert, T., Homuth, G., and Schumann, W. (2001). Development of a new integration site within the Bacillus subtilis chromosome and construction of compatible expression cassettes. *J. Bacteriol.* 183, 2696-2699.

Itaya, M., Sakaya, N., Matsunaga, S., Fujita, K., and Kaneko, S. (2006). Conjugational transfer kinetics of pLS20 between Bacillus subtilis in liquid medium. *Biosci. Biotechnol. Biochem.* 70, 740-742.

Ramachandran, G., Miguel-Arribas, A., Abia, D., Singh, P. K., Crespo, I., Gago-Cordoba, C. et al. (2017). Discovery of a new family of relaxases in Firmicutes bacteria. *PLoS Genet.* 13, e1006586.
