# Supplementary material for: The Bacillus subtilis Conjugative Plasmid pLS20 Encodes Two Ribbon-Helix-Helix Type Auxiliary Relaxosome Proteins That Are Essential for Conjugation
Source: Front Microbiol. 2017 Nov 3;8:2138. doi: 10.3389/fmicb.2017.02138 (PMC5675868; doi:10.3389/fmicb.2017.02138)
Supplement: Supplementary file 3 [file Table_3.docx]

Supplementary Material

The *Bacillus subtilis* conjugative plasmid pLS20 encodes two ribbon-helix-helix type auxiliary relaxosome proteins that are essential for conjugation

Andrés Miguel-Arribas, Jian-An Hao, Juan Roman Luque-Ortega, Gayetri Ramachandran, Jorge Val-Calvo, César Gago-Córdoba, Daniel González-Álvarez, David Abia, Carlos Alfonso, Ling J. Wu and Wilfried J.J. Meijer*

*** Correspondence:** wmeijer@cbm.csic.es

**Supplemental Table 3**

| **Supplemental Table S3. Oligonucleotides used** | | |
| --- | --- | --- |
| Name | Sequence (5’-3’) | Purpose |
| ori-up | ccccggatcCAGGCCCGGGGCTTTACGTCAA | checking transconjugants or transformants for the presence of pLS20cat, in combination with ori-dn |
| ori-dn | ttttggatccGGGAATAACAGTATACGTTAGTG | checking transconjugants or transformants for the presence of pLS20cat, in combination with ori-up |
| oGR133 | ttttactagtCAAAGTAATGTGCAGAAATCGATGA | Cloning pLS20cat gene *56* in vector pDR110 in combination with oGR134 |
| oGR134 | ttttggatccCGCGCTTTAAACACTGCGGCCAGCT | Cloning pLS20cat gene *56* in vector pDR110 in combination with oGR133 |
| oGR26 | ttttgctagcAAAAAAGATATTAGAAAGTGAGGGT | Cloning pLS20cat gene *57-58* in vector pDR110 in combination with oGR60 |
| oGR43 | ttttgctagcCAAAGTAATGTGCAGAAATCGATGA | Cloning pLS20cat genes *56-57-58* in vector pDR110 in combination with oGR60 |
| oGR60 | ttttgcatgcTCCTTTAATTTCAGAATTGCCTACC | Cloning pLS20cat genes *56-57-58* in vector pDR110 in combination with oGR43. Cloning of pLS20cat gene *58* in pDR110 in combination with oGR45. Cloning pLS20cat gene *57-58* in vector pDR110 in combination with oGR26 |
| oGR45 | ttttgctagcCTAGCAGAAGGCTAAGCAGGAAATGCAGAA | Cloning pLS20cat genes *58* in vector pDR110 in combination with oGR60 |
| oJH01 | atatccatggCAAAAGTAAAGAAGCATCTTACCTTTAGTGGTCCG | Cloning *aux2_LS20_* in pET28b+ in combination with oJH02 |
| oJH02 | aaaagtcgacTTTTTCACCTTTCTGCATTTCCTGCTT | Cloning *aux2_LS20_* in pET28b+ in combination with oJH01 |
| oJH03 | TTAACTTTAAGAAGGAGATATACCATGCCGGATCTCAACATCAAAGGTCTTTCAA | Cloning *aux1_LS20_* in pET28b+ in combination with oJH05 |
| oJH04 | aaaatctagaGAAATAATTTTGTTTAACTTTAAGAAGGAGATATACCATGCCGGATCTC | Cloning *aux1_LS20_* in pET28b+ in combination with oJH05 |
| oJH05 | aaaagtcgacTCCTTCATTTACTCTGATGAATTCGTTAAGT | Cloning *aux1_LS20_* in pET28b+ in combination with oJH03 and oJH04 |
| oJAH2b | GCTGAATGCACATAAAAAAAACATTTATG | Generation 200 bp fragment F21 in combination with oJAH6a, to study binding Aux1_LS20_ and Aux2_LS20_ to *oriT_LS20_* region. |
| oJAH3b | GAAGGAGGGGGTGGAAAAGGAAAGAGCATAAGGGG | Generation 200 bp fragment F25 in combination with oJAH7a, to study binding Aux1_LS20_ and Aux2_LS20_ to *oriT_LS20_* region |
| oJAH4b | CATCGATTTCTGCACATTACTTTGCAACG | Generation 200 bp fragment F29 in combination with oJAH8a, to study binding Aux1_LS20_ and Aux2_LS20_ to *oriT_LS20_* region |
| oJAH5b | TGAGAAAGTCCTGCCTTCCTCGCTT | Generation 200 bp fragment F33 in combination with oJAH9a, to study binding Aux1_LS20_ and Aux2_LS20_ to *oriT_LS20_* region |
| oJAH6a | ACGAGCAAAGAAATGCTAGGTGAACTTG | Generation 200 bp fragment F21 in combination with oJAH2b; to study binding Aux1_LS20_ and Aux2_LS20_ to *oriT_LS20_* region. |
| oJAH7a | CAGTTATTTTTCGTGTGCATAAAATAAAG | Generation 200 bp fragment F25 in combination with oJAH3b, to study binding Aux1_LS20_ and Aux2_LS20_ to *oriT_LS20_* region |
| oJAH8a | CAATAAATCTGGTACCACGAAAAAACAAACCGC | Generation 200 bp fragment F29 in combination with oJAH4b, to study binding Aux1_LS20_ and Aux2_LS20_ to *oriT_LS20_* region |
| oJAH9a | CCTTCGGAATCGGGGGCCGGCTTTTTGCTGCC | Generation 200 bp fragment F22 in combination with oJAH21b, to study binding Aux1_LS20_ and Aux2_LS20_ to *oriT_LS20_* region |
| oJAH21a | CTTGAAAAACGGAATGAATTATTGAAGAAAAG | Generation 200 bp fragment F33 in combination with oJAH5b, to study binding Aux1_LS20_ and Aux2_LS20_ to *oriT_LS20_* region |
| oJAH21b | TTTTTCGTGGTACCAGATTTATTGCTGA | Generation 200 bp fragment F22 in combination with oJAH21a, to study binding Aux1_LS20_ and Aux2_LS20_ to *oriT_LS20_* region |
| oJAH22a | AGAAAAGAAAGAGCAATCTCGTCATCGA | Generation 200 bp fragment F23 in combination with oJAH22b, to study binding Aux1_LS20_ and Aux2_LS20_ to *oriT_LS20_* region |
| oJAH22b | ATGGTGAAACCGCAGTGCGGTTTGTTTTTTCGTGGT | Generation 200 bp fragment F23 in combination with oJAH22a, to study binding Aux1_LS20_ and Aux2_LS20_ to *oriT_LS20_* region |
| oJAH23a | CGAAGACTAAATTTCTGTATGGAAAACAGTT | Generation 200 bp fragment F24 in combination with oJAH23b, to study binding Aux1_LS20_ and Aux2_LS20_ to *oriT_LS20_* region |
| oJAH23b | ATAAGGGGAAACTGGCACCATTTGCATGGTG | Generation 200 bp fragment F24 in combination with oJAH23a, to study binding Aux1_LS20_ and Aux2_LS20_ to *oriT_LS20_* region |
| oJAH24a | TAAAGATTTATGTGCATTTAGTTCTAAATCACCT | Generation 200 bp fragment F26 in combination with oJAH24b, to study binding Aux1_LS20_ and Aux2_LS20_ to *oriT_LS20_* region |
| oJAH24b | AAAGCCGGCCCCCGATTCCGAAGGAAGGAGGGGGTGG | Generation 200 bp fragment F26 in combination with oJAH24a, to study binding Aux1_LS20_ and Aux2_LS20_ to *oriT_LS20_* region |
| oJAH25a | AAATCACCTAAATAATGGTTGAACATAAATG | Generation 200 bp fragment F27 in combination with oJAH25b, to study binding Aux1_LS20_ and Aux2_LS20_ to *oriT_LS20_* region |
| oJAH25b | TTTCCAAGGCTTTTTGCGGCAGCAAAAAGCCGGCCCCCG | Generation 200 bp fragment F27 in combination with oJAH25a, to study binding Aux1_LS20_ and Aux2_LS20_ to *oriT_LS20_* region |
| oJAH26a | TAAATGTTTTTTTTATGTGCATTCAGCA | Generation 200 bp fragment F28 in combination with oJAH26b, to study binding Aux1_LS20_ and Aux2_LS20_ to *oriT_LS20_* region |
| oJAH26b | CGATCTGTTCAAATAAGTGTTTCTTTTTCC | Generation 200 bp fragment F28 in combination with oJAH26a, to study binding Aux1_LS20_ and Aux2_LS20_ to *oriT_LS20_* region |
| oJAH27a | ACAAACCGCACTGCGGTTTCACCATGCAA | Generation 200 bp fragment F30 in combination with oJAH27b, to study binding Aux1_LS20_ and Aux2_LS20_ to *oriT_LS20_* region |
| oJAH27b | CGGCATTTGTTAACGCTCCTTTTCATCG | Generation 200 bp fragment F30 in combination with oJAH27a, to study binding Aux1_LS20_ and Aux2_LS20_ to *oriT_LS20_* region |
| oJAH28a | GCAAATGGTGCCAGTTTCCCCTTATGCTCTTTCC | Generation 200 bp fragment F31 in combination with oJAH28b, to study binding Aux1_LS20_ and Aux2_LS20_ to *oriT_LS20_* region |
| oJAH28b | TTGAAAGACCTTTGATGTTGAGATCCGGC | Generation 200 bp fragment F31 in combination with oJAH28a, to study binding Aux1_LS20_ and Aux2_LS20_ to *oriT_LS20_* region |
| oJAH29a | GCTCTTTCCTTTTCCACCCCCTCCTTCCTTCGG | Generation 200 bp fragment F32 in combination with oJAH29b, to study binding Aux1_LS20_ and Aux2_LS20_ to *oriT_LS20_* region |
| oJAH29b | TCCGCAAGCCTATTCATTGTATCTTTTGAA | Generation 200 bp fragment F32 in combination with oJAH29a, to study binding Aux1_LS20_ and Aux2_LS20_ to *oriT_LS20_* region |
| 5´- overhang sequences are indicated in lower case and restriction sites are underlined | | |
