# Supplementary material for: The Bacillus subtilis Conjugative Plasmid pLS20 Encodes Two Ribbon-Helix-Helix Type Auxiliary Relaxosome Proteins That Are Essential for Conjugation
Source: Front Microbiol. 2017 Nov 3;8:2138. doi: 10.3389/fmicb.2017.02138 (PMC5675868; doi:10.3389/fmicb.2017.02138)
Supplement: Supplementary file 4 [file Table_4.pdf]

Table S4. MOB_L-encoding relaxase genes preceded by genes encoding homologs of $Aux2_{LS20}$ and/or $Aux1_{LS20}$

Nº	Species	MOB_L relaxase*	$Aux2_{LS20}$ homolog*	Secondary structure prediction of $Aux2_{LS20}$ homolog** and protein size	$Aux1_{LS20}$ homolog*	Secondary structure prediction of $Aux1_{LS20}$ homolog** and protein size
1	*Bacillus altitudinis*	WP_047947260 (NZ_LDPI01000047.1)	WP_047947259 (NZ_LDPI01000047.1)	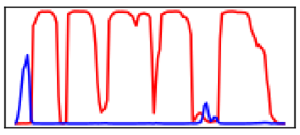 139AA	WP_047947258 (NZ_LDPI01000047.1)	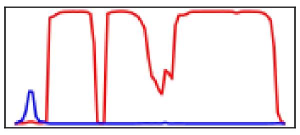 80AA
2	*Bacillus aminovorans*	WP_063974512 (NZ_LQWZ01000007.1)	WP_018395846 (NZ_LQWZ01000007.1)	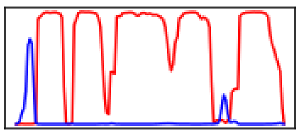 134AA	-	-
3	*Bacillus amyloliquefaciens*	WP_073982227 (NZ_CYHP01000076.1)	WP_073982226 (NZ_CYHP01000076.1)	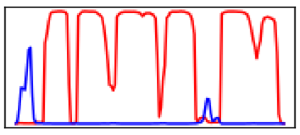 147AA	WP_032859023 (NZ_CYHP01000076.1)	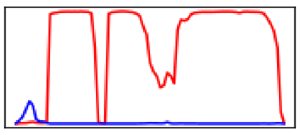 79AA
4	*Bacillus aryabhattai*	WP_088568153 (NZ_NHZZ01000039.1)	WP_088568152 (NZ_NHZZ01000039.1)	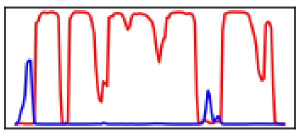 145AA	-	-
5	*Bacillus aryabhattai*	WP_043977025 (NZ_JXRC01000006.1)	WP_043977017 (NZ_JXRC01000006.1)	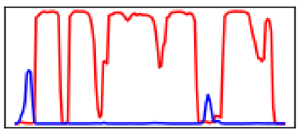 145AA	-	-
6	*Bacillus aryabhattai*	WP_047751924 (NZ_KQ087174.1)	WP_047751925 (NZ_KQ087174.1)	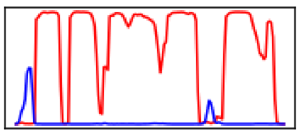 144AA	-	-
7	*Bacillus aryabhattai B8W22*	WP_074682325 (NZ_FMZY01000007.1)	WP_057275479 (NZ_FMZY01000007.1)	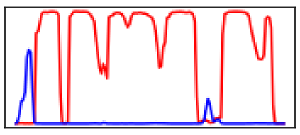 145AA	-	-
8	*Bacillus aryabhattai B8W22*	WP_074682817 (NZ_FMZY01000009.1)	WP_074682816 (NZ_FMZY01000009.1)	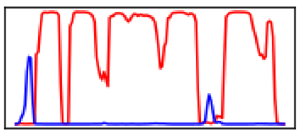 144AA	-	-
9	*Bacillus atrophaeus*	WP_061670953 (NZ_BCVV01000015.1)	WP_061670952 (NZ_BCVV01000015.1)	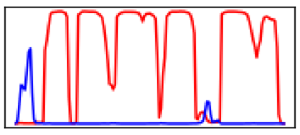 147AA	WP_061670951 (NZ_BCVV01000015.1)	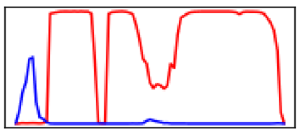 79AA
10	*Bacillus badius*	WP_041098556 (NZ_JXLO01000004.1)	WP_041098553 (NZ_JXLO01000004.1)	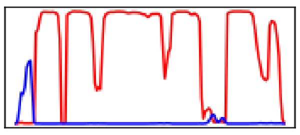 147AA	-	-
11	*Bacillus cereus*	WP_000606311 (NZ_CM000735.1)	WP_000526095 (NZ_JH792172.1)	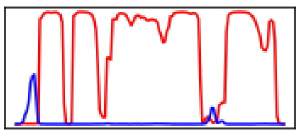 145AA	-	-
12	*Bacillus cereus*	WP_048536194 (NZ_LABH01000058.1)	WP_048536195 (NZ_LABH01000058.1)	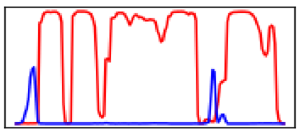 147AA	-	-
13	*Bacillus cereus*	WP_075716618 (NZ_MNKZ01000006.1)	WP_075716617 (NZ_MNKZ01000006.1)	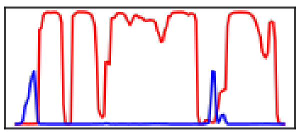 147AA	-	-

14	*Bacillus cereus*	WP_078205288 (NZ_MUAJ01000032.1)	WP_078205287 (NZ_MUAJ01000032.1)	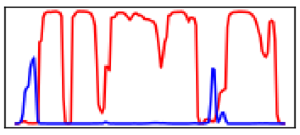 147AA	-	-
15	*Bacillus cereus*	WP_061664322 (NZ_LOMO01000249.1)	WP_061664321 (NZ_LOMO01000249.1)	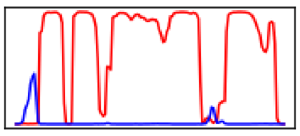 145AA	-	-
16	*Bacillus cereus*	WP_048567233 (NZ_JYPF01000010.1)	WP_048567234 (NZ_JYPF01000010.1)	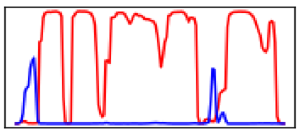 147AA	-	-
17	*Bacillus cereus*	WP_046960175 (NZ_LCYN01000033.1)	WP_046960176 (NZ_LCYN01000033.1)	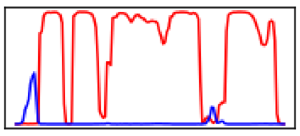 145AA	-	-
18	*Bacillus cereus*	WP_061674695 (NZ_LOMP01000081.1)	WP_061674696 (NZ_LOMP01000081.1)	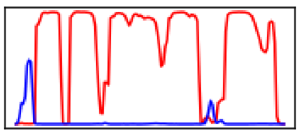 143AA	-	-
19	*Bacillus cereus*	WP_074651536 (NZ_FMZR01000016.1)	WP_074651535 (NZ_FMZR01000016.1)	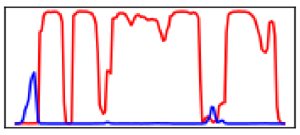 145AA	-	-
20	*Bacillus cereus*	WP_063217430 (NZ_LJKB01000004.1)	WP_063217429 (NZ_LJKB01000004.1)	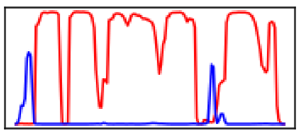 145AA	-	-
21	*Bacillus cereus*	WP_081143328 (NZ_NBNG01000016.1)	WP_081143329 (NZ_NBNG01000016.1)	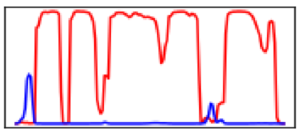 143AA	-	-
22	*Bacillus cereus*	WP_074596406 (NZ_MIEV01000011.1)	WP_074596405 (NZ_MIEV01000011.1)	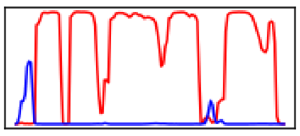 143AA	-	-
23	*Bacillus cereus*	WP_078175123 (NZ_MUAG01000030.1)	WP_078175122 (NZ_MUAG01000030.1)	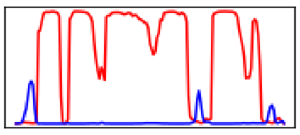 155AA	-	-
24	*Bacillus cereus*	WP_059304442 (NZ_CP011146.1)	WP_059304443 (NZ_CP011146.1)	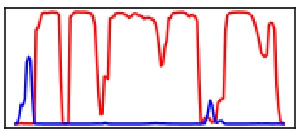 143AA	-	-
25	*Bacillus cereus*	WP_088911278 (NZ_CP016596.1)	WP_088911277 (NZ_CP016596.1)	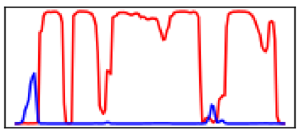 145AA	-	-
26	*Bacillus cereus*	WP_048535287 (NZ_LABH01000044.1)	WP_048535284 (NZ_LABH01000044.1)	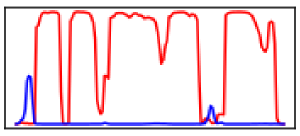 143AA	-	-
27	*Bacillus cereus*	WP_025966431 (NZ_CP015730.1)	WP_025966432 (NZ_CP015730.1)	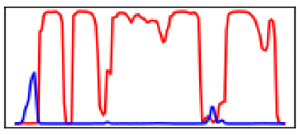 145AA	-	-

28	*Bacillus cereus*	WP_089172223 (NZ_CP018741.1)	WP_089172222 (NZ_CP018741.1)	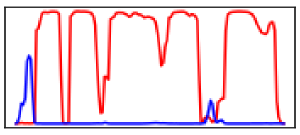 143AA	-	-
29	*Bacillus cereus*	WP_061687714 (NZ_LONG01000143.1)	WP_061687715 (NZ_LONG01000143.1)	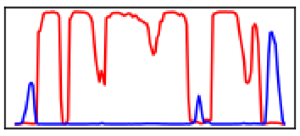 155AA	-	-
30	*Bacillus cereus*	WP_078400789 (NZ_MQTF01000036.1)	WP_078400790 (NZ_MQTF01000036.1)	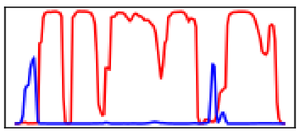 147AA	-	-
31	*Bacillus cereus*	WP_044796823 (NZ_JYCM01000089.1)	WP_044796824 (NZ_JYCM01000089.1)	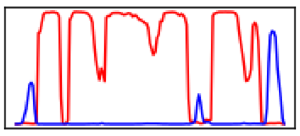 155AA	-	-
32	*Bacillus cereus*	WP_033694044 (NZ_KN049964.1)	WP_033694042 (NZ_KN049964.1)	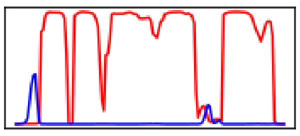 138AA	WP_033694040 (NZ_MTAT01000011.1)	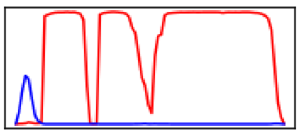 84AA
33	*Bacillus cereus*	WP_061667861 (NZ_LOBE01000191.1)	WP_061667862 (NZ_LOBE01000191.1)	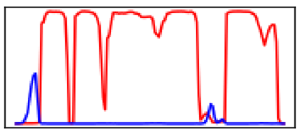 136AA	WP_061667863 (NZ_LOBE01000191.1)	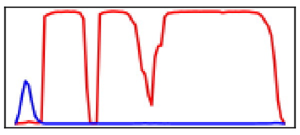 84AA
34	*Bacillus cereus*	WP_063539203 (NZ_CP011157.1)	WP_063539205 (NZ_CP011157.1)	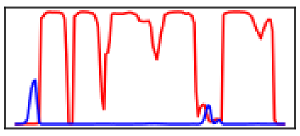 138AA	-	-
35	*Bacillus cereus*	WP_088338626 (NZ_NHVH01000062.1)	WP_088338625 (NZ_NHVH01000062.1)	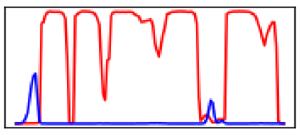 136AA	WP_088338624 (NZ_NHVH01000062.1)	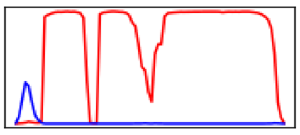 84AA
36	*Bacillus cereus BAG1O-2*	WP_000063689 (NZ_JH792323.1)	WP_000368962 (NZ_JH792323.1)	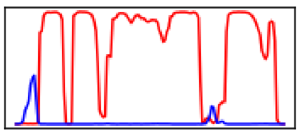 145AA	-	-
37	*Bacillus cereus BAG1O-3*	WP_016513357 (NZ_KE332539.1)	WP_016513358 (NZ_KE332539.1)	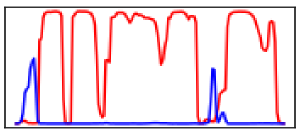 147AA	-	-
38	*Bacillus cereus BAG1X1-3*	WP_000063697 (NZ_KB976299.1)	WP_000104365 (NZ_KB976299.1)	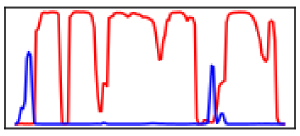 145AA	-	-
39	*Bacillus cereus BAG2O-2*	WP_000063692 (NZ_LOBA01000080.1)	WP_000368961 (NZ_LOBA01000080.1)	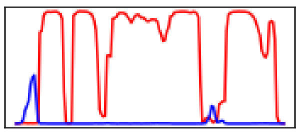 145AA	-	-
40	*Bacillus cereus BAG4X2-1*	WP_000063696 (NZ_JH804619.1)	WP_000428255 (NZ_JH804619.1)	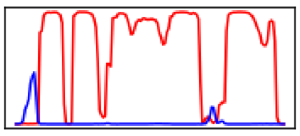 145AA	-	-
41	*Bacillus cereus BAG5O-1*	WP_001057949 (NZ_JH791947.1)	WP_000104361 (NZ_JH791947.1)	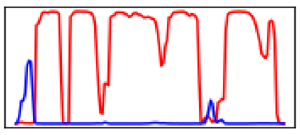 143AA	-	-

42	*Bacillus cereus BAG5X12-1*	WP_000093051 (NZ_KB976558.1)	WP_000428254 (NZ_KB976558.1)	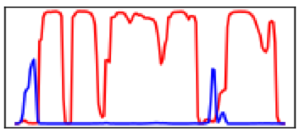 147AA	-	-
43	*Bacillus cereus CER074*	WP_002130409 (NZ_JH791992.1)	WP_002130406 (NZ_JH791992.1)	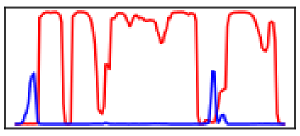 147AA	-	-
44	*Bacillus cereus HuA2-1*	WP_002204212 (NZ_JH804674.1)	WP_002204213 (NZ_JH804674.1)	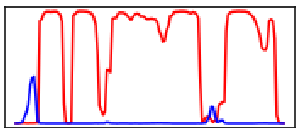 145AA	-	-
45	*Bacillus cereus HuA2-9*	WP_016093909 (NZ_KB976155.1)	WP_016093910 (NZ_KB976155.1)	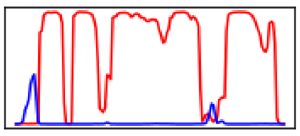 145AA	-	-
46	*Bacillus cereus HuA4-10*	WP_002151031 (NZ_JH792149.1)	WP_002151032 (NZ_JH792149.1)	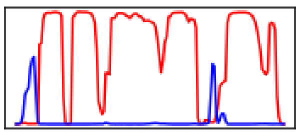 147AA	-	-
47	*Bacillus cereus HuB5-5*	WP_001057948 (NZ_JH792121.1)	WP_000526096 (NZ_JH792121.1)	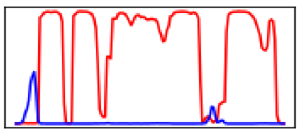 145AA	-	-
48	*Bacillus cereus MSX-A1*	WP_001057731 (NZ_JH792106.1)	WP_000428253 (NZ_JH792106.1)	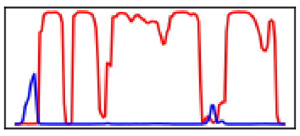 145AA	-	-
49	*Bacillus cereus MSX-A12*	WP_001095808 (NZ_JH792045.1)	WP_000038318 (NZ_JH792045.1)	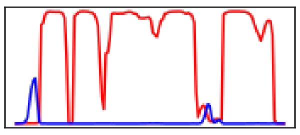 138AA	-	-
50	*Bacillus cereus Rock3-29*	EEL36934 (CM000731.1)	EEL36933 (CM000731.1)	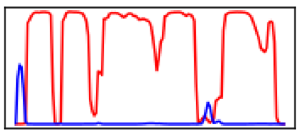 138AA	-	-
51	*Bacillus cereus VD021*	WP_016102511 (NZ_KB976282.1)	WP_016102510 (NZ_KB976282.1)	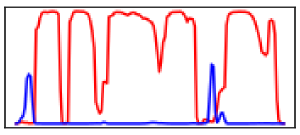 145AA	-	-
52	*Bacillus cereus VD045*	WP_001095810 (NZ_JH792088.1)	WP_000038319 (NZ_JH792088.1)	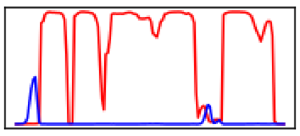 138AA	-	-
53	*Bacillus cereus VD078*	WP_002169630 (NZ_JH792252.1)	WP_002169631 (NZ_JH792252.1)	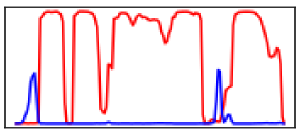 143AA	-	-
54	*Bacillus cereus VD118*	WP_016105659 (NZ_KB976799.1)	WP_016103577 (NZ_KB976799.1)	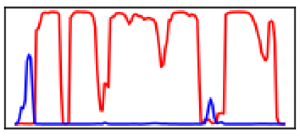 143AA	-	-
55	*Bacillus cereus VD131*	WP_016107045 (NZ_LXLJ01000097.1)	WP_016107046 (NZ_LXLJ01000097.1)	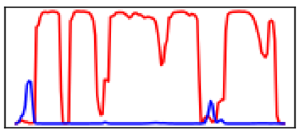 143AA	-	-

56	*Bacillus cereus VD133*	WP_016110483 (NZ_KB976176.1)	WP_016110482 (NZ_KB976176.1)	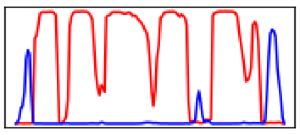 155AA	-	-
57	*Bacillus cereus VD142*	WP_002089986 (NZ_KE150048.1)	WP_002089988 (NZ_KE150048.1)	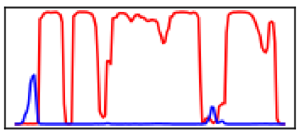 145AA	-	-
58	*Bacillus cereus VD142*	WP_016362211 (NZ_KE150063.1)	WP_002090364 (NZ_KE150063.1)	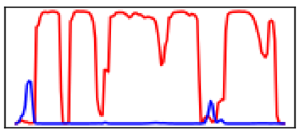 143AA	-	-
59	*Bacillus cereus VD146*	WP_016119360 (NZ_KB976675.1)	WP_016119359 (NZ_KB976675.1)	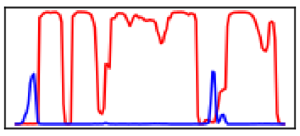 147AA	-	-
60	*Bacillus cereus VD146*	WP_016121217 (NZ_KB976679.1)	WP_016121216 (NZ_KB976679.1)	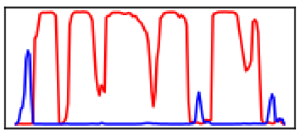 155AA	-	-
61	*Bacillus cereus VD148*	WP_000063695 (NZ_JH792158.1)	WP_000104364 (NZ_MOOP01000065.1)	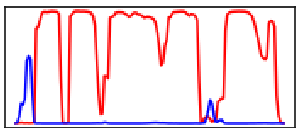 143AA	-	-
62	*Bacillus cereus VD214*	WP_000063694 (NZ_FMJK01000071.1)	WP_000651369 (NZ_FMJK01000071.1)	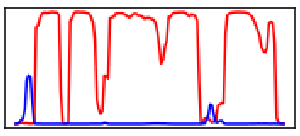 143AA	-	-
63	*Bacillus cereus VDM019*	WP_016128048 (NZ_MRWV01000027.1)	WP_016128047 (NZ_MRWV01000027.1)	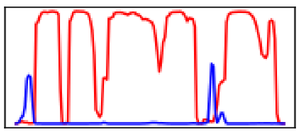 145AA	-	-
64	*Bacillus cereus VDM022*	WP_002187836 (NZ_JH791842.1)	WP_002187835 (NZ_JH791842.1)	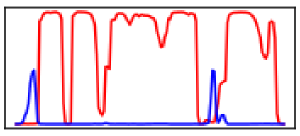 147AA	-	-
65	*Bacillus cereus VDM022*	WP_002205105 (NZ_JH791844.1)	WP_002205104 (NZ_JH791844.1)	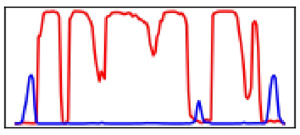 155AA	-	-
66	*Bacillus cereus VDM034*	WP_002191967 (NZ_JH791812.1)	WP_002191966 (NZ_JH791812.1)	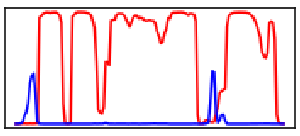 147AA	-	-
67	*Bacillus cereus VDM062*	WP_002193611 (NZ_JH791805.1)	WP_002193612 (NZ_JH791805.1)	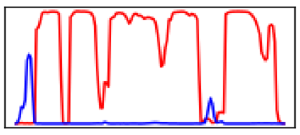 143AA	-	-
68	*Bacillus cereus group*	WP_044782914 (NZ_JYCM01000093.1)	WP_044782916 (NZ_CP020748.1)	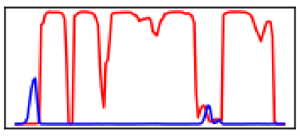 138AA	WP_085313642 (NZ_CP020748.1)	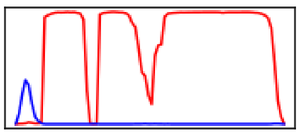 84AA
69	*Bacillus endophyticus*	WP_019392513 (NZ_ALIM01000017.1)	WP_019392514 (NZ_ALIM01000017.1)	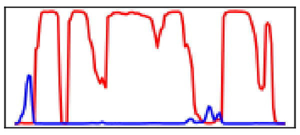 147AA	-	-

70	*Bacillus endophyticus*	WP_061802768 (NZ_FOXX01000003.1)	WP_061802769 (NZ_FOXX01000003.1)	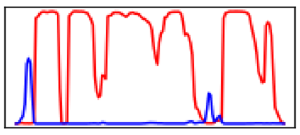 147AA	-	-
71	*Bacillus flexus*	WP_078990611 (NZ_CP016791.1)	WP_078990612 (NZ_CP016791.1)	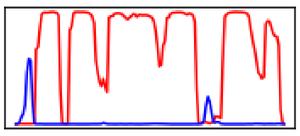 145AA	-	-
72	*Bacillus gaemokensis*	WP_033678232 (NZ_LTAQ01000007.1)	WP_033678234 (NZ_LTAQ01000007.1)	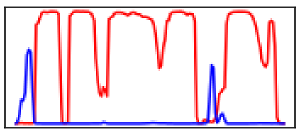 145AA	-	-
73	*Bacillus ginsengihumi*	WP_025731348 (NZ_JAGM01000050.1)	WP_025731349 (NZ_JAGM01000050.1)	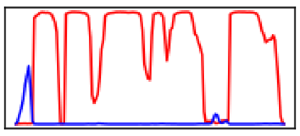 145AA	-	-
74	*Bacillus licheniformis*	WP_061578343 (NZ_LQYK01000030.1)	WP_061578342 (NZ_LQYK01000030.1)	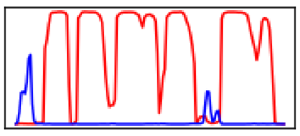 147AA	WP_061578341 (NZ_LQYK01000030.1)	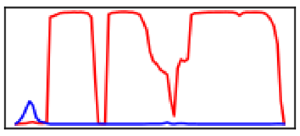 79AA
75	*Bacillus licheniformis*	WP_073461430 (NZ_MEDB01000012.1)	WP_073461429 (NZ_MEDB01000012.1)	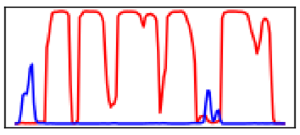 148AA	WP_073461428 (NZ_MEDB01000012.1)	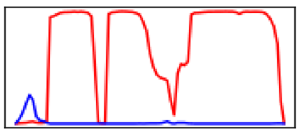 79AA
76	*Bacillus licheniformis*	WP_085059417 (NZ_JYGX01000015.1)	WP_083460654 (NZ_JYGX01000015.1)	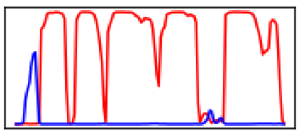 138AA	WP_054287334 (NZ_JYGX01000015.1)	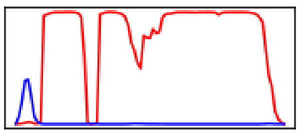 87AA
77	*Bacillus licheniformis*	WP_003186655 (NZ_CP012110.1)	WP_003186653 (NZ_CP012110.1)	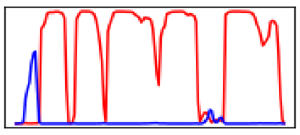 138AA	WP_003186651 (NZ_CP012110.1)	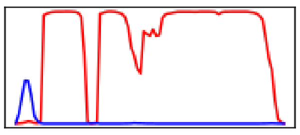 87AA
78	*Bacillus licheniformis*	WP_073461179 (NZ_MEDB01000003.1)	WP_073461180 (NZ_MEDB01000003.1)	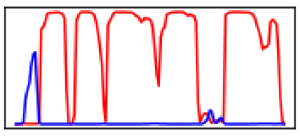 138AA	WP_073461181 (NZ_MEDB01000003.1)	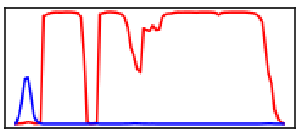 87AA
79	*Bacillus megaterium*	WP_074898597 (NZ_FOPA01000040.1)	WP_074898596 (NZ_FOPA01000040.1)	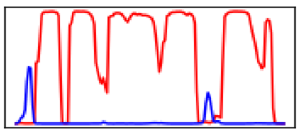 145AA	-	-
80	*Bacillus megaterium*	WP_061859914 (NZ_LUCO01000008.1)	WP_061859913 (NZ_LUCO01000008.1)	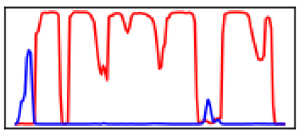 145AA	-	-
81	*Bacillus megaterium*	WP_075422499 (NZ_MRUF01000021.1)	WP_075422500 (NZ_MRUF01000021.1)	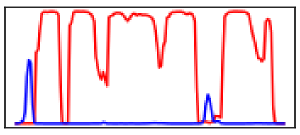 145AA	-	-
82	*Bacillus megaterium*	WP_078082417 (NZ_CP018877.1)	WP_047934013 (NZ_CP018877.1)	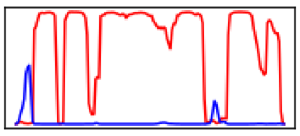 159AA	-	-
83	*Bacillus megaterium*	WP_060746292 (NZ_LRPG01000101.1)	WP_060746291 (NZ_LRPG01000101.1)	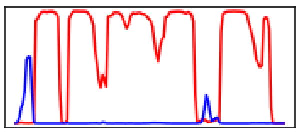 146AA	-	-

84	*Bacillus megaterium*	WP_061860157 (NZ_LUCO01000013.1)	WP_061860156 (NZ_LUCO01000013.1)	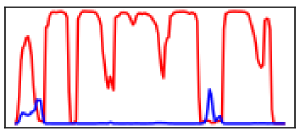 152AA	-	-
85	*Bacillus megaterium*	WP_075422174 (NZ_MRUF01000011.1)	WP_075422173 (NZ_MRUF01000011.1)	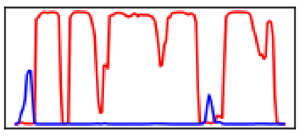 144AA	-	-
86	*Bacillus megaterium*	WP_047933298 (NZ_LDPJ01000046.1)	WP_047933296 (NZ_LDPJ01000046.1)	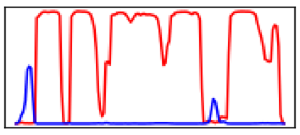 141AA	-	-
87	*Bacillus megaterium NBRC 15308*	WP_034655615 (NZ_BCVB01000018.1)	WP_034655612 (NZ_BCVB01000018.1)	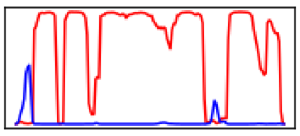 159AA	-	-
88	*Bacillus megaterium NBRC 15308*	WP_034656079 (NZ_CP009921.1)	WP_034656077 (NZ_CP009921.1)	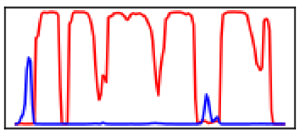 146AA	-	-
89	*Bacillus mycoides*	WP_078214262 (NZ_MUAR01000030.1)	WP_078214263 (NZ_MUAR01000030.1)	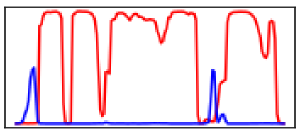 147AA	-	-
90	*Bacillus mycoides*	WP_088010068 (NZ_MRWT01000021.1)	WP_088010069 (NZ_MRWT01000021.1)	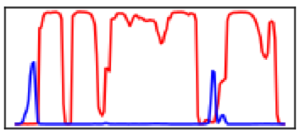 147AA	-	-
91	*Bacillus mycoides*	WP_088290976 (NZ_NIUP01000002.1)	WP_088290975 (NZ_NIUP01000002.1)	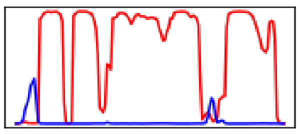 145AA	-	-
92	*Bacillus mycoides*	WP_078203788 (NZ_MUAH01000015.1)	WP_078203787 (NZ_MUAH01000015.1)	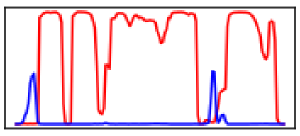 147AA	-	-
93	*Bacillus mycoides*	WP_078204107 (NZ_MUAH01000029.1)	WP_078204106 (NZ_MUAH01000029.1)	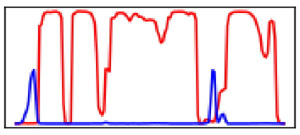 147AA	-	-
94	*Bacillus mycoides*	WP_041488843 (NZ_CM000743.1)	WP_080548498 (NZ_CM000743.1)	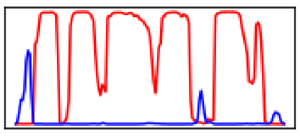 153AA	-	-
95	*Bacillus mycoides*	WP_085313577 (NZ_CP020747.1)	WP_085313578 (NZ_CP020747.1)	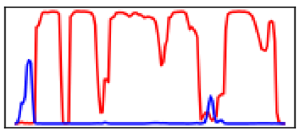 143AA	-	-
96	*Bacillus mycoides*	WP_060749750 (NZ_LRPH01000035.1)	WP_060749749 (NZ_LRPH01000035.1)	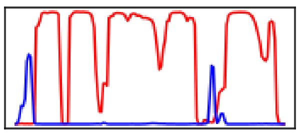 145AA	-	-
97	*Bacillus mycoides*	WP_033796261 (NZ_CM000744.1)	WP_033796262 (NZ_CM000744.1)	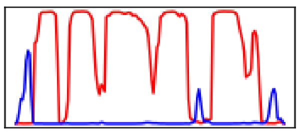 155AA	-	-

98	*Bacillus mycoides*	WP_088010098 (NZ_MRWT01000023.1)	WP_070168322 (NZ_MRWT01000023.1)	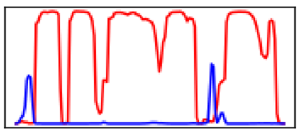 145AA	-	-
99	*Bacillus mycoides*	WP_033798168 (NZ_JMQD01000068.1)	WP_033798169 (NZ_JMQD01000068.1)	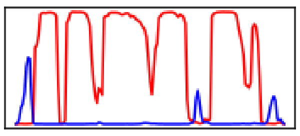 153AA	-	-
100	*Bacillus mycoides*	WP_088265974 (NZ_NIZC01000030.1)	WP_088265975 (NZ_NIZC01000030.1)	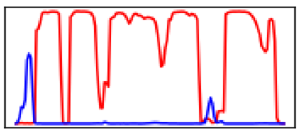 143AA	-	-
101	*Bacillus mycoides*	WP_078178728 (NZ_MUAK01000060.1)	WP_078178727 (NZ_MUAK01000060.1)	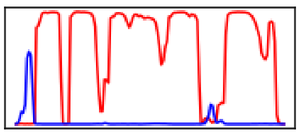 143AA	-	-
102	*Bacillus mycoides*	WP_041488765 (NZ_CM000743.1)	WP_080548490 (NZ_CM000743.1)	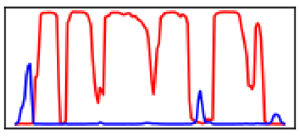 151AA	-	-
103	*Bacillus mycoides*	WP_042978675 (NZ_JMQC01000007.1)	WP_042978676 (NZ_JMQC01000007.1)	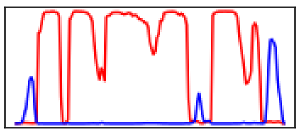 155AA	-	-
104	*Bacillus mycoides*	WP_085313641 (NZ_CP020748.1)	WP_044782916 (NZ_CP020748.1)	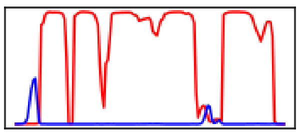 138AA	-	-
105	*Bacillus mycoides Rock3-17*	EEM10632 (CM000744.1)	EEM10631 (CM000744.1)	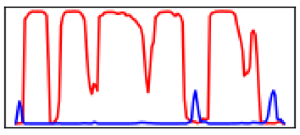 149AA	-	-
106	*Bacillus pseudomycoides*	WP_088094468 (NZ_MWPX01000038.1)	WP_088094469 (NZ_MWPX01000038.1)	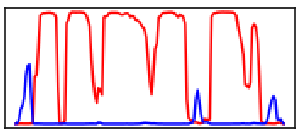 153AA	-	-
107	*Bacillus pseudomycoides DSM 12442*	WP_006097284 (NZ_CM000745.1)	WP_033799439 (NZ_CM000745.1)	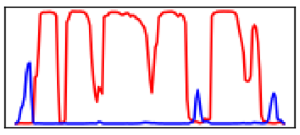 153AA	-	-
108	*Bacillus smithii*	WP_061778547 (NZ_BCVY01000066.1)	WP_061778546 (NZ_BCVY01000066.1)	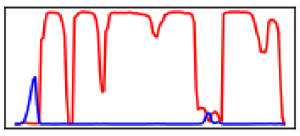 138AA	WP_061778545 (NZ_BCVY01000066.1)	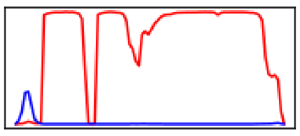 86AA
109	*Bacillus sp. 112mf*	SEB22412 (FNRB01000037.1)	SEB22409 (FNRB01000037.1)	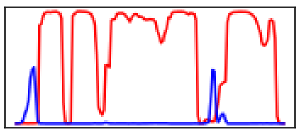 147AA	-	-
110	*Bacillus sp. 166amftsu*	SDZ40837 (FNQA01000029.1)	SDZ40847 (FNQA01000029.1)	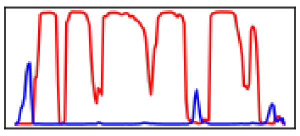 154AA	-	-
111	*Bacillus sp. 4048*	WP_071743591 (NZ_NIZB01000015.1)	WP_071743592 (NZ_NIZB01000015.1)	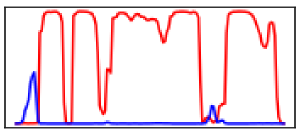 145AA	-	-

112	*Bacillus sp. 5mfcol3.1*	SFM43181 (FOST01000060.1)	SFM43218 (FOST01000060.1)	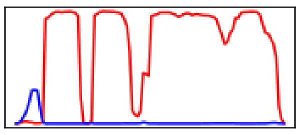 100AA	-	-
113	*Bacillus sp. 5mfcol3.1*	SFM43667 (FOST01000062.1)	SFM43696 (FOST01000062.1)	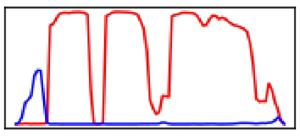 87AA	-	-
114	*Bacillus sp. CDB3*	OQR53283 (ALBR01000127.1)	OQR53282 (ALBR01000127.1)	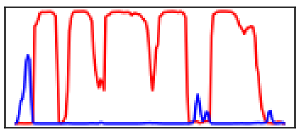 156AA	-	-
115	*Bacillus sp. FDAARGOS_235*	WP_080609136 (NZ_CP020434.1)	WP_080609134 (NZ_CP020434.1)	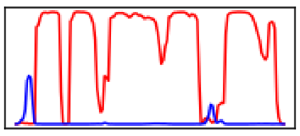 143AA	-	-
116	*Bacillus sp. FJAT-20673*	WP_063577872 (NZ_LWJH01000005.1)	WP_063577873 (NZ_LWJH01000005.1)	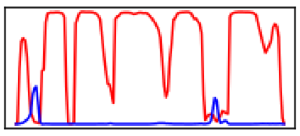 144AA	-	-
117	*Bacillus sp. FJAT-21351*	WP_053488181 (NZ_LITO01000005.1)	WP_053488180 (NZ_LITO01000005.1)	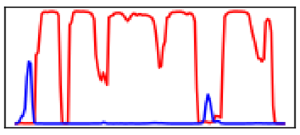 145AA	-	-
118	*Bacillus sp. FJAT-27231*	WP_049666858 (NZ_LFZU01000002.1)	WP_049666860 (NZ_LFZU01000002.1)	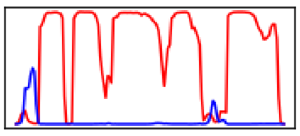 144AA	-	-
119	*Bacillus sp. H1a*	WP_025147935 (NZ_AYMH01000016.1)	WP_025147936 (NZ_AYMH01000016.1)	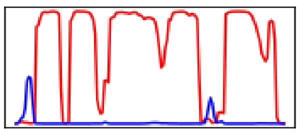 143AA	-	-
120	*Bacillus sp. K2I17*	WP_088609614 (NZ_NJGF01000025.1)	WP_088609613 (NZ_NJGF01000025.1)	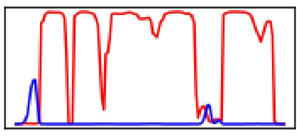 138AA	WP_001101622 (NZ_NJGF01000025.1)	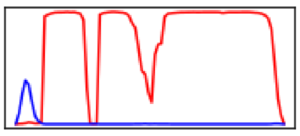 84AA
121	*Bacillus sp. KCTC 13219*	WP_066163345 (NZ_LUFJ01000001.1)	WP_066163346 (NZ_LUFJ01000001.1)	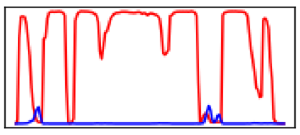 154AA	-	-
122	*Bacillus sp. LK2*	WP_048375747 (NZ_LDUK01000051.1)	WP_048375749 (NZ_LDUK01000051.1)	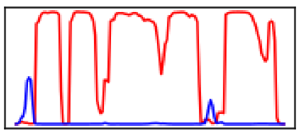 143AA	-	-
123	*Bacillus sp. Leaf75*	WP_055989992 (NZ_LMRL01000008.1)	WP_055989989 (NZ_LMRL01000008.1)	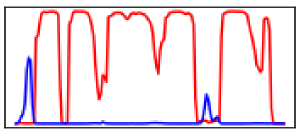 146AA	-	-
124	*Bacillus sp. NH11B*	WP_071770376 (NZ_MAOG01000021.1)	WP_016128428 (NZ_MAOG01000021.1)	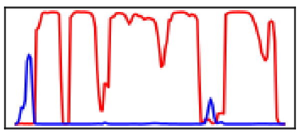 143AA	-	-
125	*Bacillus sp. NH24A2*	WP_071717409 (NZ_MAOI01000007.1)	WP_071717410 (NZ_MAOI01000007.1)	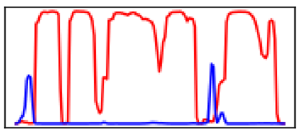 145AA	-	-

126	*Bacillus sp. P4B(2010)*	SDJ00360 (FNDU01000018.1)	SDJ00390 (FNDU01000018.1)	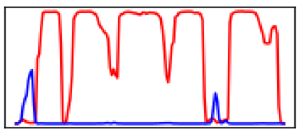 149AA	-	-
127	*Bacillus sp. RP1137*	WP_029325353 (NZ_AXZS01000034.1)	WP_029325352 (NZ_AXZS01000034.1)	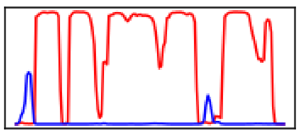 145AA	-	-
128	*Bacillus sp. RP1137*	WP_029325432 (NZ_AXZS01000037.1)	WP_029325431 (NZ_AXZS01000037.1)	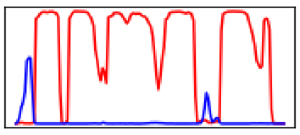 146AA	-	-
129	*Bacillus sp. Root131*	WP_001057732 (NZ_NFCT01000035.1)	WP_000651369 (NZ_FMJK01000071.1)	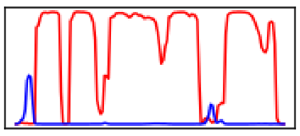 143AA	-	-
130	*Bacillus sp. Root239*	WP_057242255 (NZ_LMJS01000004.1)	WP_057242256 (NZ_LMJS01000004.1)	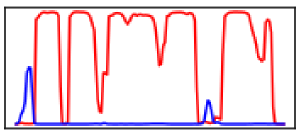 145AA	-	-
131	*Bacillus sp. Root239*	WP_057242331 (NZ_LMJS01000004.1)	WP_057242330 (NZ_LMJS01000004.1)	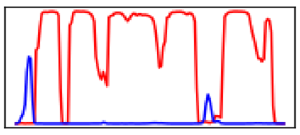 145AA	-	-
132	*Bacillus sp. SGD-V-25*	WP_041114324 (NZ_MWMM01000021.1)	WP_041114325 (NZ_MWMM01000021.1)	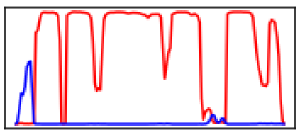 147AA	-	-
133	*Bacillus sp. Soil531*	WP_057275478 (NZ_LMSY01000033.1)	WP_057275479 (NZ_FMZY01000007.1)	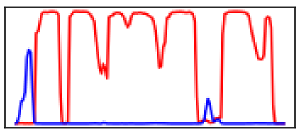 145AA	-	-
134	*Bacillus sp. TD42*	WP_071744295 (NZ_MACH01000036.1)	WP_071744294 (NZ_MACH01000036.1)	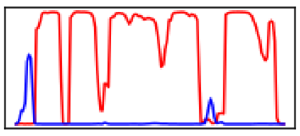 143AA	-	-
135	*Bacillus sp. UNCCL81*	SFD59801 (FOMF01000012.1)	SFD59782 (FOMF01000012.1)	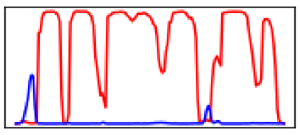 153AA	-	-
136	*Bacillus sp. ok634*	WP_002191648 (NZ_MUAI01000041.1)	WP_002191647 (NZ_MRWV01000034.1)	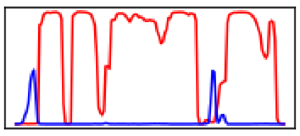 147AA	-	-
137	*Bacillus sp. yr331*	WP_000063691 (NZ_MWMG01000037.1)	WP_000104363 (NZ_MWMG01000037.1)	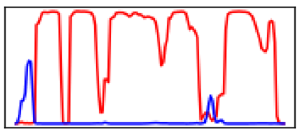 143AA	-	-
138	*Bacillus subtilis*	WP_072176350 (NZ_CYHS01000062.1)	WP_072176349 (NZ_CYHS01000062.1)	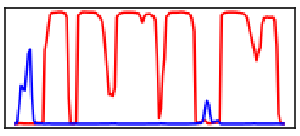 147AA	WP_072176348 (NZ_CYHS01000062.1)	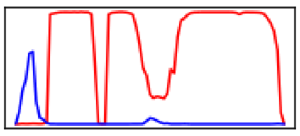 79AA
139	*Bacillus subtilis*	WP_069479544 (NZ_MQSR01000011.1)	WP_069479545 (NZ_MQSR01000011.1)	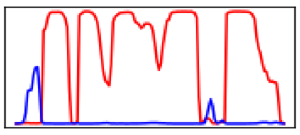 136AA	-	-

140	*Bacillus subtilis*	WP_041335083 (NZ_JXHN01000031.1)	WP_041335084 (NZ_JXHN01000031.1)	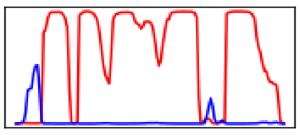 136AA	-	-
141	*Bacillus subtilis Miyagi-4*	WP_013603221 (NZ_CP017764.1)	WP_013603220 (NZ_CP021893.1)	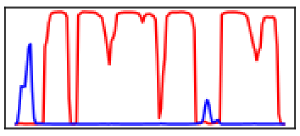 147AA	WP_013603219 (NZ_CP021893.1)	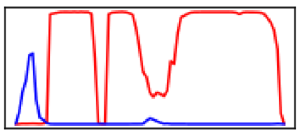 79AA
142	*Bacillus subtilis group*	WP_025809625 (NZ_CP022288.1)	WP_025809623 (NZ_CP022288.1)	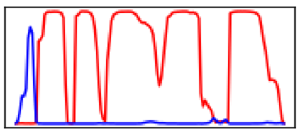 130AA	WP_075178023 (NZ_CM007615.1)	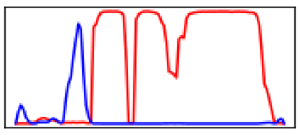 108AA
143	*Bacillus subtilis subsp. subtilis*	WP_088272624 (NZ_CP021893.1)	WP_013603220 (NZ_CP021893.1)	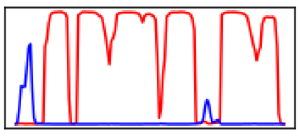 147AA	-	-
144	*Bacillus thuringiensis*	WP_012263715 (NZ_MSFC01000065.1)	WP_012263714 (NZ_MSFC01000065.1)	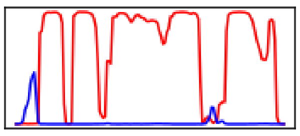 145AA	-	-
145	*Bacillus thuringiensis*	WP_071714076 (NZ_LZRA01000051.1)	WP_071714075 (NZ_LZRA01000051.1)	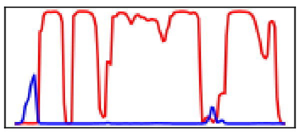 145AA	-	-
146	*Bacillus thuringiensis*	ANS51746 (CP015351.1)	ANS51747 (CP015351.1)	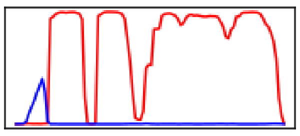 102AA	-	-
147	*Bacillus thuringiensis*	WP_029442968 (NZ_AYSM01000019.1)	WP_029442967 (NZ_AYSM01000019.1)	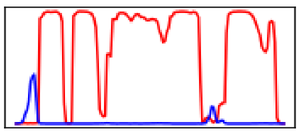 145AA	-	-
148	*Bacillus thuringiensis*	WP_070174355 (NZ_LXLL01000068.1)	WP_070174356 (NZ_LXLL01000068.1)	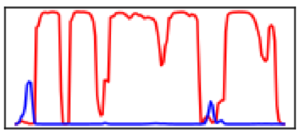 143AA	-	-
149	*Bacillus thuringiensis*	WP_050845700 (NZ_CP012103.1)	WP_050845701 (NZ_CP012103.1)	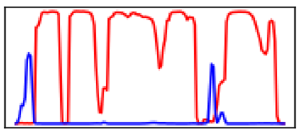 145AA	-	-
150	*Bacillus thuringiensis IBL 200*	WP_001095809 (NZ_NFEB01000143.1)	WP_000038317 (NZ_NFEB01000143.1)	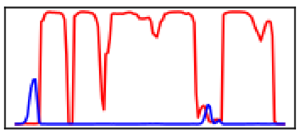 138AA	WP_000382812 (NZ_NFEB01000143.1)	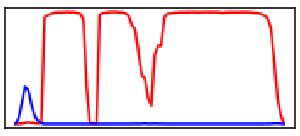 84AA
151	*Bacillus thuringiensis MC28*	WP_000063693 (NC_018688.1)	WP_000526094 (NC_018688.1)	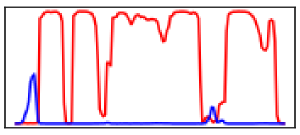 145AA	-	-
152	*Bacillus thuringiensis serovar argentinensis*	WP_088081678 (NZ_MOOO01000132.1)	WP_088081677 (NZ_MOOO01000132.1)	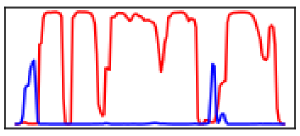 147AA	-	-
153	*Bacillus thuringiensis serovar asturiensis*	WP_088045307 (NZ_NFDI01000056.1)	WP_088045308 (NZ_NFDI01000056.1)	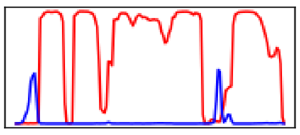 143AA	-	-

154	*Bacillus thuringiensis serovar cameroun*	WP_087950768 (NZ_NFCI01000130.1)	WP_087950767 (NZ_NFCI01000130.1)	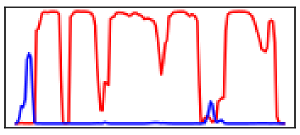 143AA	-	-
155	*Bacillus thuringiensis serovar cameroun*	WP_087950772 (NZ_NFCI01000131.1)	WP_087950771 (NZ_NFCI01000131.1)	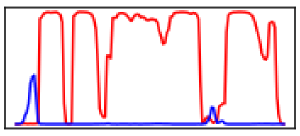 145AA	-	-
156	*Bacillus thuringiensis serovar cameroun*	WP_087951308 (NZ_NFCI01000152.1)	WP_087951307 (NZ_NFCI01000152.1)	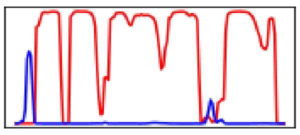 143AA	-	-
157	*Bacillus thuringiensis serovar graciosensis*	WP_061685796 (NZ_NFDP01000061.1)	WP_061685795 (NZ_NFDP01000061.1)	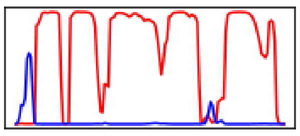 143AA	-	-
158	*Bacillus thuringiensis serovar iberica*	WP_086401212 (NZ_MOOP01000065.1)	WP_000104364 (NZ_MOOP01000065.1)	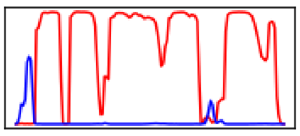 143AA	-	-
159	*Bacillus thuringiensis serovar iberica*	WP_086401514 (NZ_MOOP01000101.1)	WP_086401513 (NZ_MOOP01000101.1)	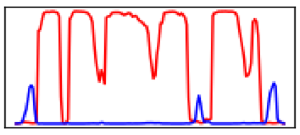 155AA	-	-
160	*Bacillus thuringiensis serovar jegathesan*	WP_086404729 (NZ_MOOS01000189.1)	WP_079004575 (NZ_MOOS01000189.1)	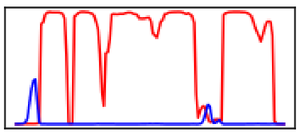 138AA	WP_033694040 (NZ_MTAT01000011.1)	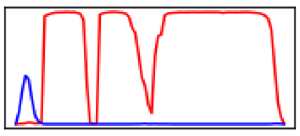 84AA
161	*Bacillus thuringiensis serovar konkukian*	WP_087997510 (NZ_MOOF01000305.1)	WP_087997509 (NZ_MOOF01000305.1)	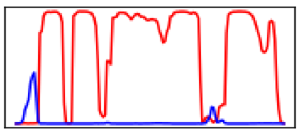 145AA	-	-
162	*Bacillus thuringiensis serovar konkukian*	WP_087994898 (NZ_MOOF01000107.1)	WP_087994897 (NZ_MOOF01000107.1)	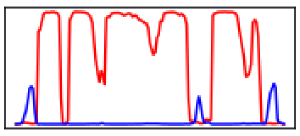 155AA	-	-
163	*Bacillus thuringiensis serovar kumamotoensis*	WP_086391238 (NZ_NFEH01000017.1)	WP_086391239 (NZ_NFEH01000017.1)	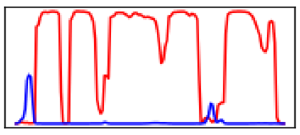 143AA	-	-
164	*Bacillus thuringiensis serovar leesis*	WP_086413233 (NZ_MOOG01000065.1)	WP_086413232 (NZ_MOOG01000065.1)	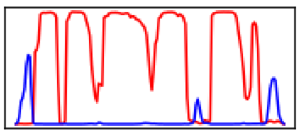 153AA	-	-
165	*Bacillus thuringiensis serovar malayensis*	WP_087968928 (NZ_MOOI01000097.1)	WP_087968929 (NZ_MOOI01000097.1)	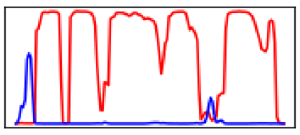 143AA	-	-
166	*Bacillus thuringiensis serovar medellin*	WP_088066791 (NZ_MOOV01000106.1)	WP_088066794 (NZ_MOOV01000106.1)	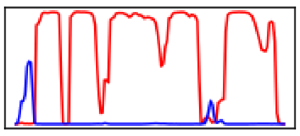 143AA	-	-
167	*Bacillus thuringiensis serovar medellin*	WP_088070537 (NZ_MOOV01000270.1)	WP_088070539 (NZ_MOOV01000270.1)	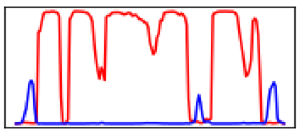 155AA	-	-

168	*Bacillus thuringiensis serovar monterrey*	WP_000093052 (NZ_NFCF01000119.1)	WP_000104362 (NZ_NFCF01000119.1)	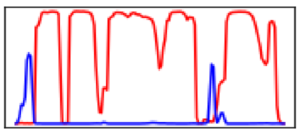 145AA	-	-
169	*Bacillus thuringiensis serovar navarrensis*	WP_088031984 (NZ_NFDG01000103.1)	WP_088031982 (NZ_NFDG01000103.1)	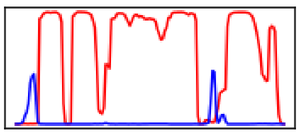 147AA	-	-
170	*Bacillus thuringiensis serovar ostriniae*	WP_086422685 (NZ_NFEJ01000062.1)	WP_086422686 (NZ_NFEJ01000062.1)	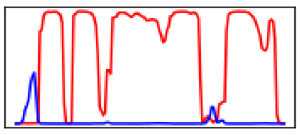 145AA	-	-
171	*Bacillus thuringiensis serovar pirenaica*	WP_086424293 (NZ_MOON01000113.1)	WP_086424294 (NZ_MOON01000113.1)	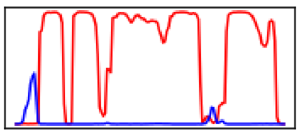 145AA	-	-
172	*Bacillus thuringiensis serovar sinensis*	WP_088047468 (NZ_MOOW01000072.1)	WP_088047469 (NZ_MOOW01000072.1)	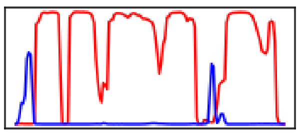 145AA	-	-
173	*Bacillus thuringiensis serovar thompsoni*	WP_062804446 (NZ_NFEC01000144.1)	WP_062804445 (NZ_NFEC01000144.1)	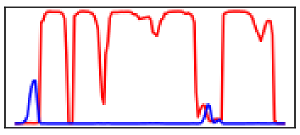 138AA	WP_001101622 (NZ_NJGF01000025.1)	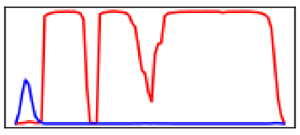 84AA
174	*Bacillus thuringiensis serovar yosoo*	WP_087966572 (NZ_NFDN01000063.1)	WP_017674549 (NZ_NFDN01000063.1)	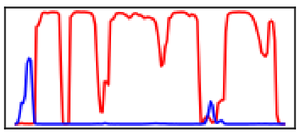 143AA	-	-
175	*Bacillus thuringiensis serovar yunnanensis*	WP_086411207 (NZ_MOOH01000402.1)	WP_086411208 (NZ_MOOH01000402.1)	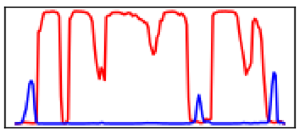 155AA	-	-
176	*Bacillus thuringiensis serovar zhaodongensis*	WP_086390906 (NZ_MOOR01000114.1)	WP_086390907 (NZ_MOOR01000114.1)	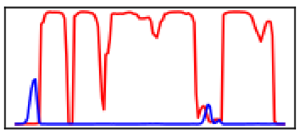 138AA	WP_001101623 (NZ_MOOR01000114.1)	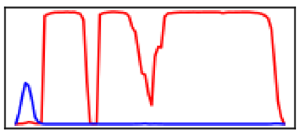 84AA
177	*Bacillus thuringiensis str. Al Hakam*	WP_018783109 (NZ_CP009650.1)	WP_018783110 (NZ_CP009650.1)	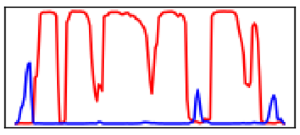 153AA	-	-
178	*Bacillus toyonensis*	WP_085450839 (NZ_MSAB01000007.1)	WP_085450840 (NZ_MSAB01000007.1)	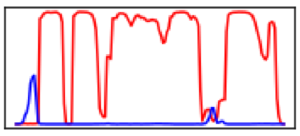 145AA	-	-
179	*Bacillus weihenstephanensis*	WP_078177147 (NZ_MUAI01000045.1)	WP_078177146 (NZ_MUAI01000045.1)	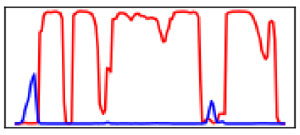 145AA	-	-
180	*Bacillus weihenstephanensis*	WP_088099813 (NZ_FMAK01000080.1)	WP_002166613 (NZ_FMAK01000080.1)	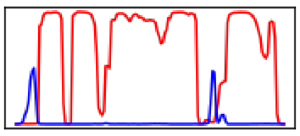 147AA	-	-
181	*Bacillus weihenstephanensis*	WP_070146072 (NZ_LXLU01000131.1)	WP_070146071 (NZ_LXLV01000105.1)	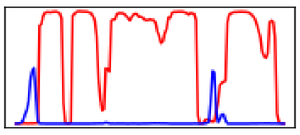 147AA	-	-

182	*Bacillus weihenstephanensis*	KEZ79999 (JNLY01000024.1)	KEZ80000 (JNLY01000024.1)	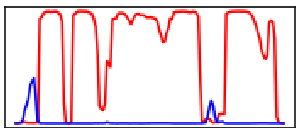 145AA	-	-
183	*Bacillus weihenstephanensis*	WP_070146009 (NZ_LXLV01000043.1)	WP_070146008 (NZ_LXLV01000043.1)	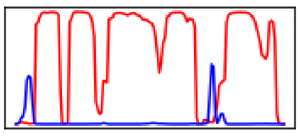 145AA	-	-
184	*Bacillus weihenstephanensis*	WP_088040842 (NZ_FLZU01000033.1)	WP_088040843 (NZ_FLZU01000033.1)	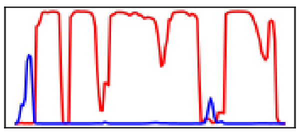 143AA	-	-
185	*Bacillus weihenstephanensis*	WP_070146035 (NZ_LXLV01000027.1)	WP_002166613 (NZ_FMAK01000080.1)	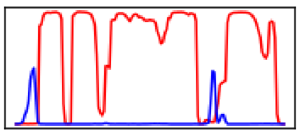 147AA	-	-
186	*Clostridiales bacterium 41_12_two_minus*	OKZ70040 (MNRD01000129.1)	OKZ70041 (MNRD01000129.1)	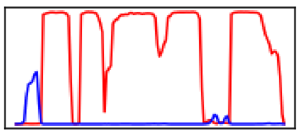 128AA	-	-
187	*Clostridiales bacterium KLE1615*	WP_066557635 (NZ_KV441294.1)	WP_066557632 (NZ_KV441294.1)	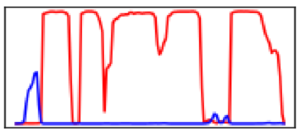 128AA	-	-
188	*Clostridium baratii*	WP_055208748 (NZ_CZBO01000008.1)	WP_055208747 (NZ_CZBO01000008.1)	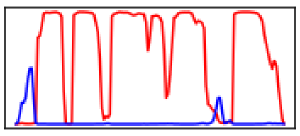 135AA	-	-
189	*Clostridium bifermentans ATCC 19299*	WP_021430704 (NZ_AVNB01000039.1)	WP_021430703 (NZ_AVNB01000039.1)	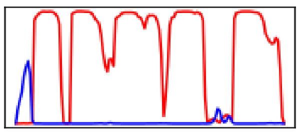 130AA	WP_021430702 (NZ_AVNB01000039.1)	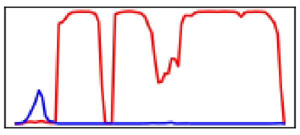 82AA
190	*Clostridium bifermentans ATCC 638*	WP_021434459 (NZ_AVNC01000022.1)	WP_021434439 (NZ_AVNC01000022.1)	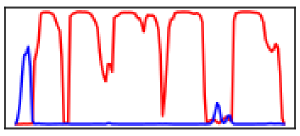 129AA	WP_021434422 (NZ_AVNC01000022.1)	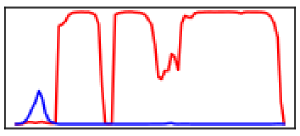 82AA
191	*Clostridium botulinum*	WP_061302148 (NZ_LFQW01000045.1)	WP_061302149 (NZ_LFQW01000045.1)	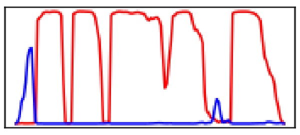 136AA	WP_061302150 (NZ_LFQW01000045.1)	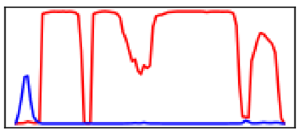 91AA
192	*Clostridium botulinum*	AIW54804 (KJ776581.1)	AIW54803 (KJ776581.1)	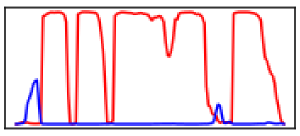 139AA	-	-
193	*Clostridium botulinum*	WP_035784585 (NZ_JQOJ01000142.1)	WP_035784583 (NZ_JQOJ01000142.1)	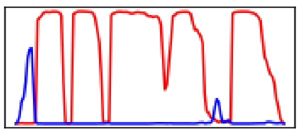 136AA	WP_012431092 (NZ_JQOJ01000142.1)	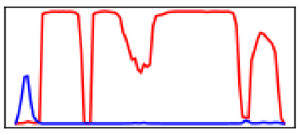 91AA
194	*Clostridium botulinum*	WP_049177648 (NZ_JUWA01000027.1)	WP_049177647 (NZ_JUWA01000027.1)	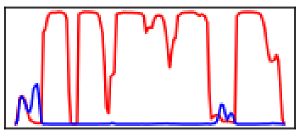 137AA	WP_034867246 (NZ_JUWA01000027.1)	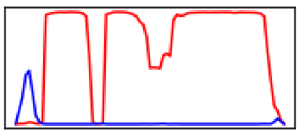 81AA
195	*Clostridium botulinum B str. Eklund 17B(NRP)*	WP_012431069 (NC_018653.1)	WP_012431064 (NC_018653.1)	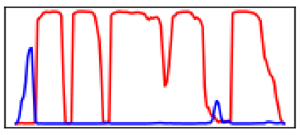 136AA	WP_012431092 (NZ_JQOJ01000142.1)	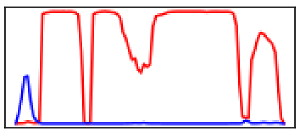 91AA

196	*Clostridium difficile*	WP_066024784 (NZ_MBGB01000023.1)	WP_066024783 (NZ_MBGB01000023.1)	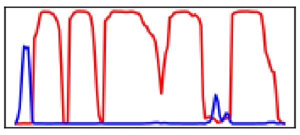<br>130AA	-	-
197	*Clostridium difficile P49*	WP_021424744 (NZ_AVMN01000016.1)	WP_021424743 (NZ_AVMN01000016.1)	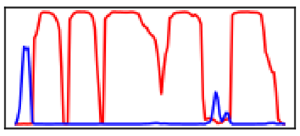<br>130AA	-	-
198	*Clostridium disporicum*	WP_055291686 (NZ_CYYT01000020.1)	WP_055291685 (NZ_CYYT01000020.1)	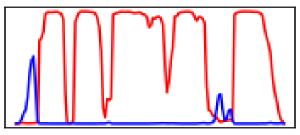<br>136AA	-	-
199	*Clostridium disporicum*	WP_055276942 (NZ_CYZV01000023.1)	WP_055276941 (NZ_CYZV01000023.1)	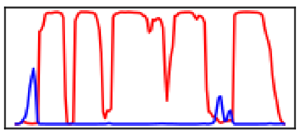<br>136AA	-	-
200	*Clostridium paraputrificum*	WP_051196084 (NZ_KE384123.1)	WP_051196085 (NZ_KE384123.1)	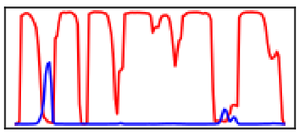<br>144AA	WP_034867246 (NZ_JUWA01000027.1)	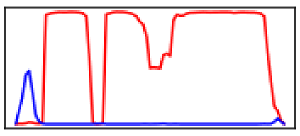<br>81AA
201	*Clostridium perfringens*	WP_075841154 (NZ_MARQ01000073.1)	WP_003482626 (NZ_MARQ01000073.1)	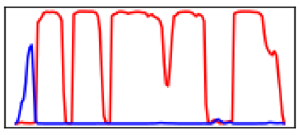<br>135AA	WP_024269848 (NZ_MARQ01000073.1)	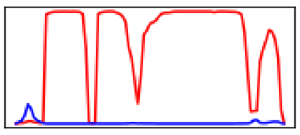<br>89AA
202	*Clostridium perfringens*	WP_060796918 (NZ_KQ956330.1)	WP_060796917 (NZ_KQ956330.1)	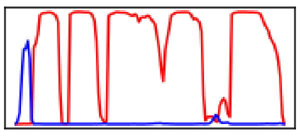<br>134AA	-	-
203	*Clostridium perfringens*	WP_061416268 (NZ_MASC01000049.1)	WP_075841142 (NZ_MARQ01000067.1)	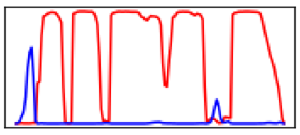<br>136AA	WP_061416272 (NZ_MASC01000049.1)	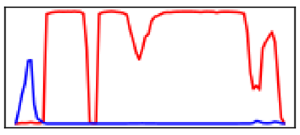<br>88AA
204	*Clostridium perfringens*	WP_060794916 (NZ_KQ956182.1)	WP_060794917 (NZ_CP019576.1)	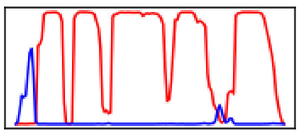<br>134AA	-	-
205	*Clostridium perfringens*	WP_075810005 (NZ_MARR01000120.1)	WP_003480912 (NZ_MARR01000120.1)	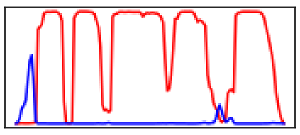<br>134AA	-	-
206	*Clostridium perfringens*	WP_078209970 (NZ_CP019576.1)	WP_061426485 (NZ_CP019576.1)	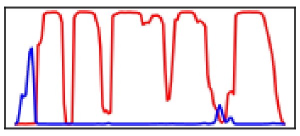<br>134AA	-	-
207	*Clostridium perfringens*	WP_061415001 (NZ_MASC01000048.1)	WP_061414998 (NZ_MASC01000048.1)	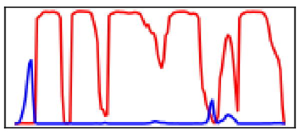<br>139AA	-	-
208	*Clostridium perfringens B str. ATCC 3626*	WP_003458987 (NZ_ABDV01000021.1)	WP_003458975 (NZ_ABDV01000021.1)	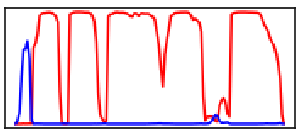<br>134AA	-	-
209	*Clostridium perfringens B str. ATCC 3626*	WP_003457695 (NZ_ABDV01000012.1)	WP_003457771 (NZ_ABDV01000012.1)	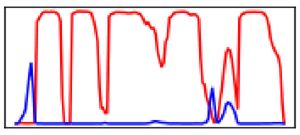<br>139AA	-	-

210	*Clostridium perfringens C str. JGS1495*	WP_003453260 (NZ_ABDU01000064.2)	WP_003453340 (NZ_ABDU01000064.2)	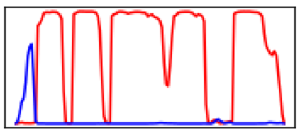 135AA	WP_003453436 (NZ_ABDU01000064.2)	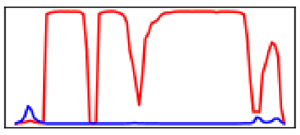 88AA
211	*Clostridium perfringens D str. JGS1721*	WP_003474246 (NZ_ABOO01000010.1)	WP_004460120 (NZ_ABOO01000010.1)	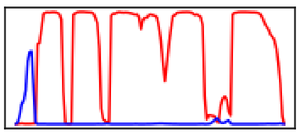 136AA	-	-
212	*Clostridium perfringens D str. JGS1721*	WP_003476279 (NZ_ABOO01000058.1)	WP_003476285 (NZ_ABOO01000058.1)	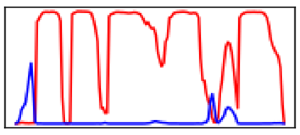 139AA	-	-
213	*Clostridium perfringens D str. JGS1721*	WP_003476060 (NZ_ABOO01000047.1)	WP_003476062 (NZ_ABOO01000047.1)	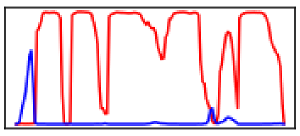 139AA	-	-
214	*Clostridium perfringens E str. JGS1987*	WP_003464984 (NZ_ABDW01000026.1)	WP_003464931 (NZ_ABDW01000026.1)	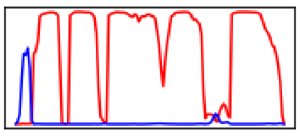 134AA	-	-
215	*Clostridium perfringens E str. JGS1987*	WP_003464757 (NZ_ABDW01000025.1)	WP_003464743 (NZ_ABDW01000025.1)	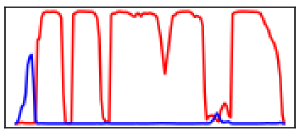 136AA	-	-
216	*Clostridium perfringens E str. JGS1987*	WP_003461650 (NZ_ABDW01000002.1)	WP_003461588 (NZ_ABDW01000002.1)	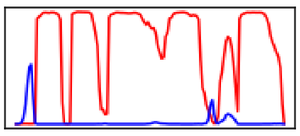 139AA	-	-
217	*Clostridium perfringens F262*	WP_003482620 (NZ_CM001479.1)	WP_003482626 (NZ_MARQ01000073.1)	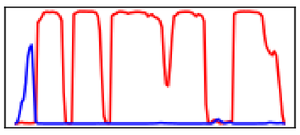 135AA	-	-
218	*Clostridium perfringens F262*	WP_003480913 (NZ_CM001477.1)	WP_003480912 (NZ_MARR01000120.1)	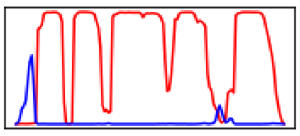 134AA	-	-
219	*Clostridium perfringens WAL-14572*	WP_004461164 (NZ_JH594541.1)	WP_004461165 (NZ_JH594541.1)	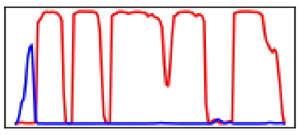 135AA	WP_004461166 (NZ_JH594541.1)	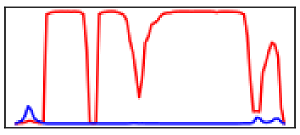 88AA
220	*Clostridium sordellii*	WP_057553361 (NZ_CDNQ01000030.1)	WP_057553362 (NZ_CDNQ01000030.1)	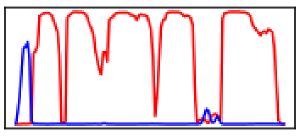 136AA	WP_057553363 (NZ_CDNQ01000030.1)	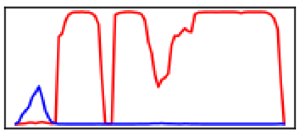 82AA
221	*Clostridium sordellii*	WP_057560209 (NZ_CELH01000008.1)	WP_057560208 (NZ_CELH01000008.1)	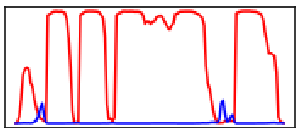 142AA	-	-
222	*Clostridium sordellii*	CEK36627 (LN681233.1)	CEK36626 (LN681233.1)	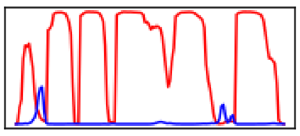 142AA	-	-
223	*Clostridium sordellii*	CEK36536 (CDLK01000054.1)	CEK36535 (CDLK01000054.1)	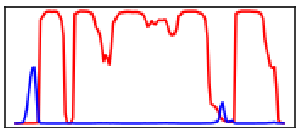 141AA	-	-

224	*Clostridium sordellii*	WP_057560165 (NZ_CELH01000007.1)	WP_057560166 (NZ_CELH01000007.1)	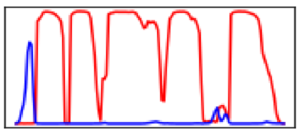 137AA	-	-
225	*Clostridium sordellii 8483*	WP_054630257 (NZ_CDNU01000030.1)	WP_021121840 (NZ_CDNU01000030.1)	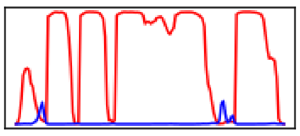 142AA	-	-
226	*Clostridium sordellii 8483*	KLR51157 (AJXR01000838.1)	KLR51156 (AJXR01000838.1)	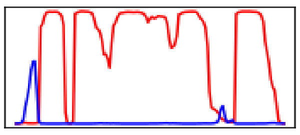 141AA	-	-
227	*Clostridium sordellii VPI 9048*	WP_021127617 (NZ_AQGJ01000153.1)	WP_021127616 (NZ_AQGJ01000153.1)	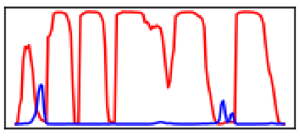 142AA	-	-
228	*Domibacillus sp. S6*	WP_069939977 (NZ_MAMP01000026.1)	WP_069939976 (NZ_MAMP01000026.1)	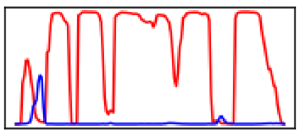 155AA	-	-
229	*Enterococcus*	WP_086304200 (NZ_NIBK01000001.1)	WP_086304202 (NZ_NIBK01000001.1)	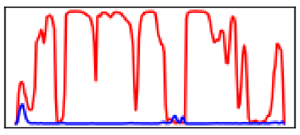 182AA	WP_086304204 (NZ_NIBK01000001.1)	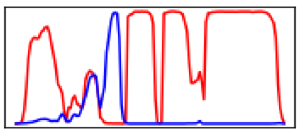 128AA
230	*Enterococcus casseliflavus 14-MB-W-14*	WP_016610085 (NZ_KE350240.1)	WP_016610086 (NZ_KE350240.1)	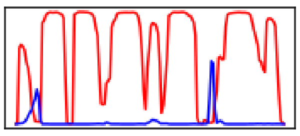 152AA	-	-
231	*Enterococcus faecalis*	WP_085390652 (NZ_MSQN01000020.1)	WP_085390650 (NZ_MSQN01000020.1)	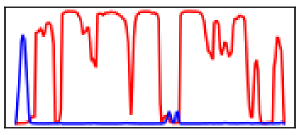 185AA	WP_080348497 (NZ_MSQN01000020.1)	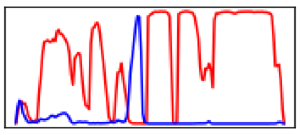 151AA
232	*Enterococcus faecalis*	WP_075582998 (NZ_FPDZ01000016.1)	WP_016616629 (NZ_FPDZ01000016.1)	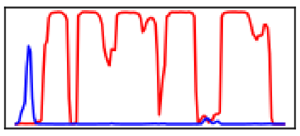 147AA	WP_057086699 (NZ_LKGS01000035.1)	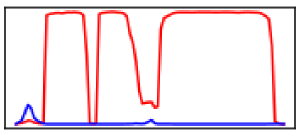 88AA
233	*Enterococcus faecalis*	WP_081115927 (NZ_MJEC01000007.1)	WP_081115926 (NZ_MJEC01000007.1)	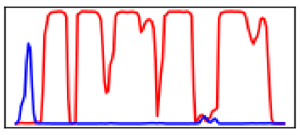 149AA	WP_010831089 (NZ_MJEC01000007.1)	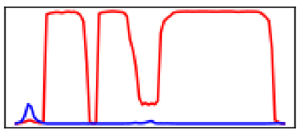 88AA
234	*Enterococcus faecalis 02-MB-BW-10*	WP_016616630 (NZ_KE351241.1)	WP_016616629 (NZ_FPDZ01000016.1)	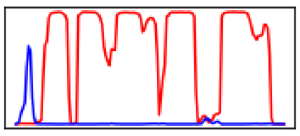 147AA	-	-
235	*Enterococcus faecalis 02-MB-BW-10*	WP_016616489 (NZ_KE351312.1)	WP_016616488 (NZ_KE351312.1)	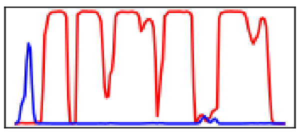 149AA	WP_010831089 (NZ_MJEC01000007.1)	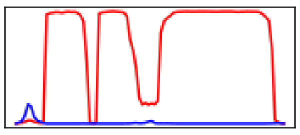 88AA
236	*Enterococcus faecalis 06-MB-DW-09*	EPH98227 (KE351352.1)	EPH98226 (KE351352.1)	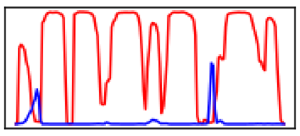 152AA	-	-
237	*Enterococcus faecalis 918*	WP_002358469 (NZ_CP019512.1)	WP_002358242 (NZ_MJBV01000010.1)	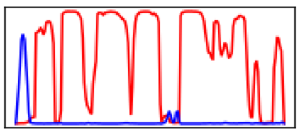 185AA	WP_002358468 (NZ_MJBX01000020.1)	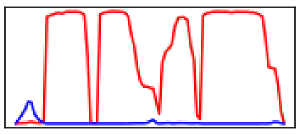 87AA

238	*Enterococcus faecalis D32*	WP_014862505 (NC_018223.1)	WP_014862504 (NC_018223.1)	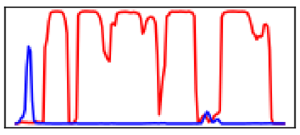 147AA	WP_014862503 (NZ_MIQF01000045.1)	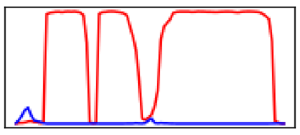 88AA
239	*Enterococcus faecalis EnGen0080*	WP_010711773 (NZ_KB932374.1)	WP_010711774 (NZ_KB932374.1)	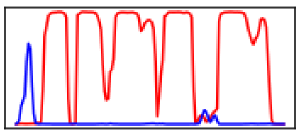 149AA	WP_010711775 (NZ_KB932374.1)	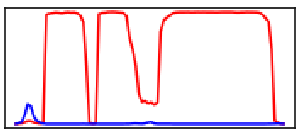 88AA
240	*Enterococcus faecalis EnGen0112*	WP_010715713 (NZ_MJBX01000020.1)	WP_010785147 (NZ_MJBX01000020.1)	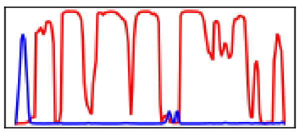 185AA	WP_002358468 (NZ_MJBX01000020.1)	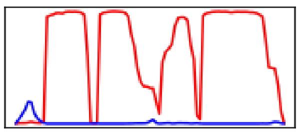 87AA
241	*Enterococcus faecalis EnGen0338*	WP_010717251 (NZ_MSQH01000021.1)	WP_010717250 (NZ_KB932571.1)	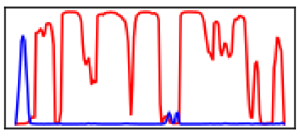 185AA	WP_002358241 (NZ_MSQH01000021.1)	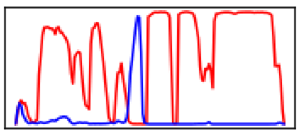 151AA
242	*Enterococcus faecalis EnGen0341*	WP_010815379 (NZ_KB944525.1)	WP_010831088 (NZ_KB947474.1)	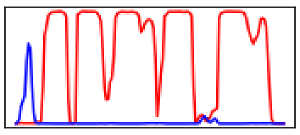 149AA	-	-
243	*Enterococcus faecalis EnGen0364*	WP_010818214 (NZ_KB944656.1)	WP_010818215 (NZ_KB944656.1)	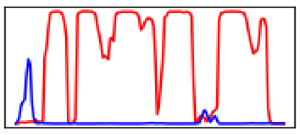 149AA	WP_010818216 (NZ_KB944656.1)	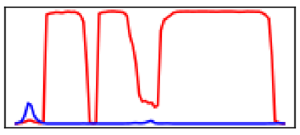 88AA
244	*Enterococcus faecium*	WP_086325252 (NZ_NGLB01000004.1)	WP_086325251 (NZ_NGLB01000004.1)	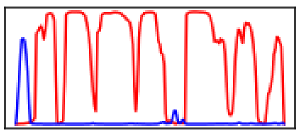 182AA	WP_086325250 (NZ_NGLB01000004.1)	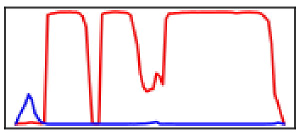 85AA
245	*Enterococcus faecium*	WP_086333149 (NZ_NGMG01000002.1)	WP_086333148 (NZ_NGMG01000002.1)	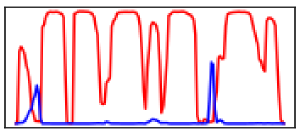 152AA	-	-
246	*Enterococcus faecium 13.SD.W.09*	EPH63194 (KE350296.1)	EPH63195 (KE350296.1)	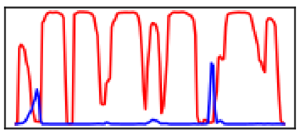 152AA	-	-
247	*Enterococcus faecium R497*	WP_002350170 (NZ_MSIB01000042.1)	WP_002350169 (NZ_MSIB01000042.1)	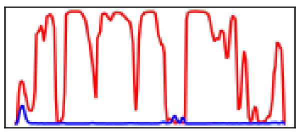 182AA	WP_002350168 (NZ_MSIB01000042.1)	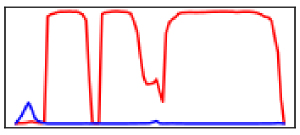 85AA
248	*Enterococcus gilvus*	OJG41140 (JXKO01000016.1)	OJG41141 (JXKO01000016.1)	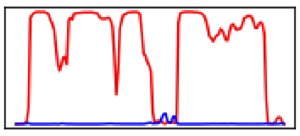 148AA	-	-
249	*Enterococcus gilvus ATCC BAA-350*	WP_010782477 (NZ_JXKO01000016.1)	WP_010782478 (NZ_JXKO01000016.1)	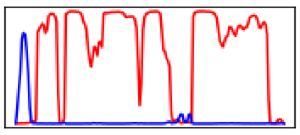 172AA	WP_010782479 (NZ_JXKO01000016.1)	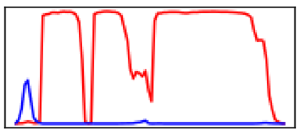 90AA
250	*Enterococcus pallens ATCC BAA-351*	WP_010760170 (NZ_JXKX01000025.1)	WP_010760169 (NZ_JXKX01000025.1)	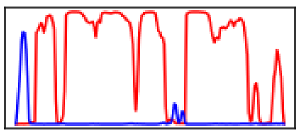 182AA	WP_016250098 (NZ_JXKX01000025.1)	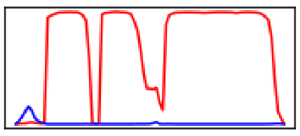 85AA
251	*Enterococcus ratti*	WP_071856131 (NZ_JXLB01000024.1)	WP_071856130 (NZ_JXLB01000024.1)	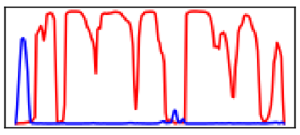 182AA	WP_071856129 (NZ_JXLB01000024.1)	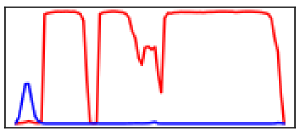 84AA

252	*Enterococcus rivorum*	WP_069697423 (NZ_MIEK01000005.1)	WP_084386993 (NZ_MIEK01000005.1)	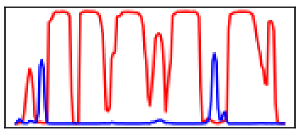 155AA	-	-
253	*Enterococcus sp. 5B3_DIV0040*	WP_086337833 (NZ_NGLC01000001.1)	WP_086337832 (NZ_NGLC01000001.1)	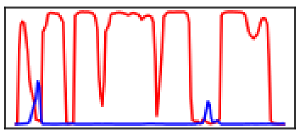 159AA	-	-
254	*Enterococcus sp. 7L76*	WP_015543650 (NZ_MJBV01000010.1)	WP_002358242 (NZ_MJBV01000010.1)	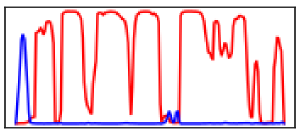 185AA	-	-
255	*Enterococcus sp. 8E11_MSG4843*	WP_087665168 (NZ_NIBP01000005.1)	WP_087665167 (NZ_NIBP01000005.1)	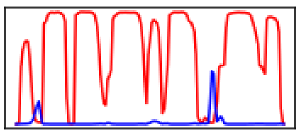 153AA	-	-
256	*Eubacterium sp. ER2*	WP_052083966 (NZ_JPJE01000014.1)	WP_033125282 (NZ_JPJE01000014.1)	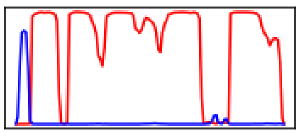 131AA	-	-
257	*Exiguobacterium acetylicum*	WP_050678824 (NZ_LFQN01000031.1)	WP_050678825 (NZ_LFQN01000031.1)	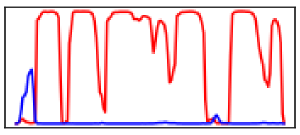 151AA	-	-
258	*Exiguobacterium aurantiacum*	WP_029336116 (NZ_JNIQ01000002.1)	WP_051638970 (NZ_JNIQ01000002.1)	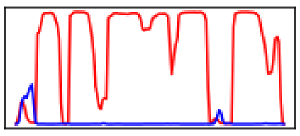 149AA	-	-
259	*Exiguobacterium indicum*	WP_075643085 (NZ_MPSZ01000008.1)	WP_075643084 (NZ_MPSZ01000008.1)	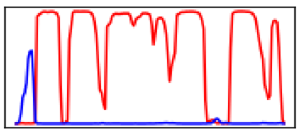 151AA	-	-
260	*Exiguobacterium marinum*	WP_026824333 (NZ_JHZT01000004.1)	WP_051545906 (NZ_JHZT01000004.1)	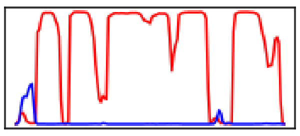 149AA	-	-
261	*Exiguobacterium oxidotolerans*	WP_088838313 (NZ_CP022239.1)	WP_088838312 (NZ_CP022239.1)	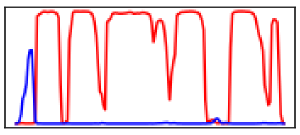 151AA	-	-
262	*Exiguobacterium pavilionensis*	WP_058765878 (NZ_JMEH01000014.1)	WP_058765877 (NZ_JMEH01000014.1)	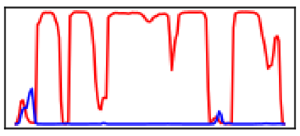 149AA	-	-
263	*Exiguobacterium pavilionensis*	WP_029596486 (NZ_JNIP01000002.1)	WP_051627821 (NZ_JNIP01000002.1)	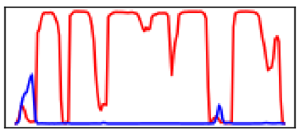 149AA	-	-
264	*Exiguobacterium pavilionensis*	WP_021065848 (NZ_ATCL01000012.1)	WP_021065849 (NZ_ATCL01000012.1)	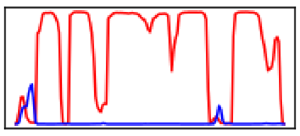 149AA	-	-
265	*Exiguobacterium profundum*	WP_074037999 (NZ_MRSV01000002.1)	WP_074037998 (NZ_MRSV01000002.1)	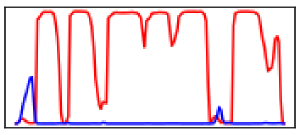 149AA	-	-

266	*Exiguobacterium sp. 8-11-1*	WP_024372255 (NZ_ATKK01000012.1)	WP_024372256 (NZ_ATKK01000012.1)	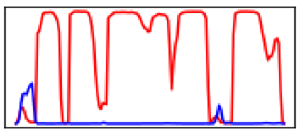 149AA	-	-
267	*Exiguobacterium sp. AB2*	WP_034808439 (NZ_JNAA01000081.1)	WP_051622693 (NZ_JNAA01000081.1)	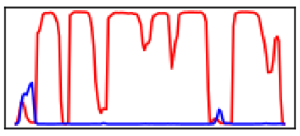 149AA	-	-
268	*Exiguobacterium sp. BMC-KP*	WP_053451913 (NZ_LGIW01000011.1)	WP_053451912 (NZ_LGIW01000011.1)	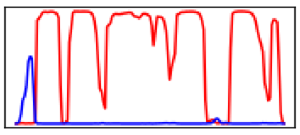 151AA	-	-
269	*Exiguobacterium sp. NG55*	WP_031424837 (NZ_JPOD01000005.1)	WP_051829474 (NZ_JPOD01000005.1)	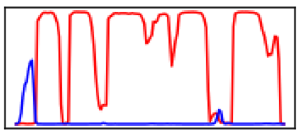 149AA	-	-
270	*Exiguobacterium sp. NG55*	WP_031424731 (NZ_JPOD01000005.1)	WP_031424733 (NZ_JPOD01000005.1)	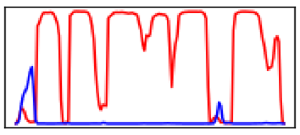 149AA	-	-
271	*Exiguobacterium sp. NIOC09*	WP_058266049 (NZ_FMYN01000007.1)	WP_058266050 (NZ_FMYN01000007.1)	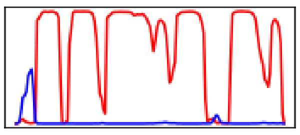 151AA	-	-
272	*Exiguobacterium sp. OS-77*	WP_035399428 (NZ_BARY01000020.1)	WP_035399425 (NZ_BARY01000020.1)	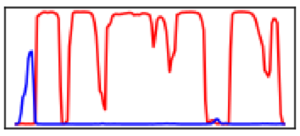 151AA	-	-
273	*Exiguobacterium sp. SH31*	WP_071399712 (NZ_LYTG01000114.1)	WP_071399713 (NZ_LYTG01000114.1)	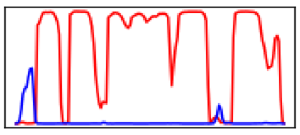 149AA	-	-
274	*Exiguobacterium sp. SH31*	WP_084813005 (NZ_LYTG01000045.1)	WP_071398596 (NZ_LYTG01000045.1)	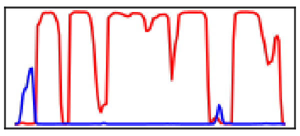 149AA	-	-
275	*Exiguobacterium undae*	WP_082892472 (NZ_LVVL01000007.1)	WP_064506603 (NZ_LVVL01000007.1)	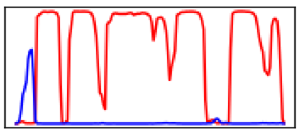 151AA	-	-
276	*Exiguobacterium undae*	WP_081775981 (NZ_JHZU01000007.1)	WP_026831512 (NZ_JHZU01000007.1)	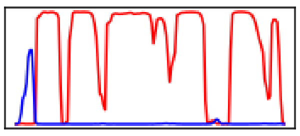 151AA	-	-
277	*Exiguobacterium undae*	WP_028105229 (NZ_LVVL01000005.1)	WP_028105230 (NZ_LVVL01000005.1)	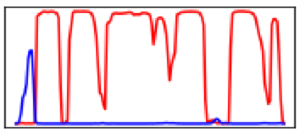 151AA	-	-
278	*Halobacillus alkaliphilus*	SFF88493 (FOOG01000012.1)	SFF88472 (FOOG01000012.1)	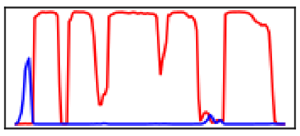 142AA	-	-
279	*Halobacillus dabanensis*	WP_075038110 (NZ_FOSB01000015.1)	WP_075038111 (NZ_FOSB01000015.1)	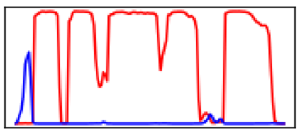 142AA	-	-

280	*Halobacillus dabanensis*	WP_035507940 (NZ_LT629827.1)	WP_035507942 (NZ_LT629827.1)	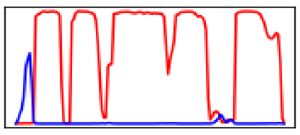 135AA	-	-
281	*Halobacillus halophilus DSM 2266*	WP_014644920 (NZ_CP022106.1)	WP_014644919 (NZ_CP022106.1)	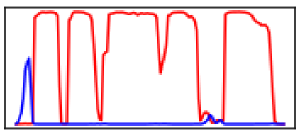 142AA	-	-
282	*Halobacillus halophilus DSM 2266*	WP_014641918 (NZ_CP022106.1)	WP_014641919 (NZ_CP022106.1)	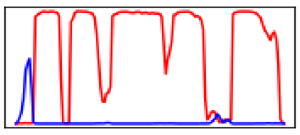 137AA	-	-
283	*Halobacillus hunanensis*	WP_079529945 (NZ_FVZB01000007.1)	WP_079529946 (NZ_FVZB01000007.1)	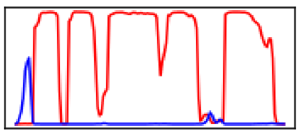 142AA	-	-
284	*Halobacillus sp. BAB-2008*	WP_008637125 (NZ_ANPF01000049.1)	WP_008637124 (NZ_ANPF01000049.1)	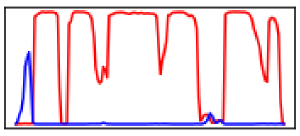 142AA	-	-
285	*Halobacillus sp. BBL2006*	WP_035548780 (NZ_JRNX01000506.1)	WP_035548783 (NZ_JRNX01000506.1)	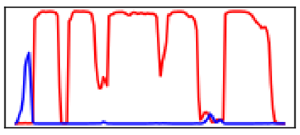 142AA	-	-
286	*Jeotgalibacillus sp. WS 4628*	WP_050182282 (NZ_LAHL01000022.1)	WP_050182283 (NZ_LAHL01000022.1)	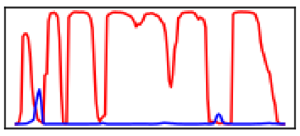 148AA	-	-
287	*Lactobacillus camelliae DSM 22697 = JCM 13995*	WP_054663362 (NZ_AYZJ01000024.1)	WP_054663359 (NZ_AYZJ01000024.1)	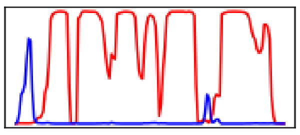 147AA	WP_054663354 (NZ_AYZJ01000024.1)	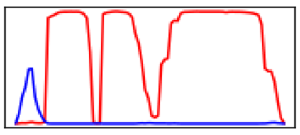 84AA
288	*Lactobacillus casei*	WP_047107641 (NZ_LCUN01000023.1)	WP_019884467 (NZ_LCUN01000023.1)	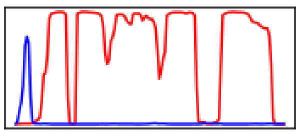 145AA	WP_019884468 (NZ_LCUN01000023.1)	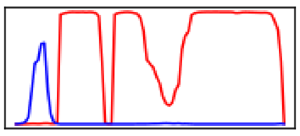 85AA
289	*Lactobacillus casei*	WP_049172362 (NZ_JUPZ01000166.1)	WP_049172364 (NZ_JUPZ01000166.1)	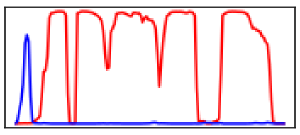 145AA	WP_049172366 (NZ_JUPZ01000166.1)	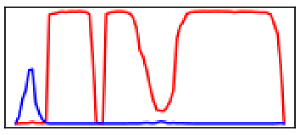 81AA
290	*Lactobacillus casei*	WP_075761296 (NZ_LOJN01000168.1)	WP_003589992 (NZ_LOJN01000168.1)	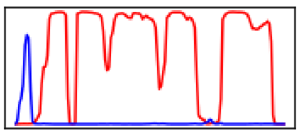 145AA	-	-
291	*Lactobacillus casei*	WP_049146572 (NZ_JUQW01000187.1)	WP_049146575 (NZ_JUQW01000187.1)	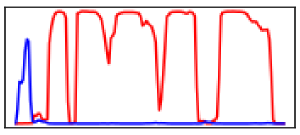 145AA	-	-
292	*Lactobacillus casei*	WP_063557999 (NZ_LTDP01000068.1)	WP_063557998 (NZ_LTDP01000068.1)	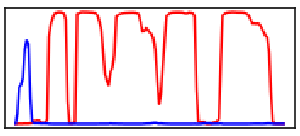 145AA	WP_063557997 (NZ_LTDP01000068.1)	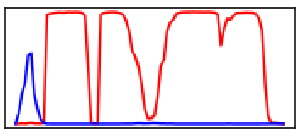 86AA
293	*Lactobacillus casei*	WP_060612217 (NZ_LNQD01000038.1)	WP_039141251 (NZ_LNQD01000038.1)	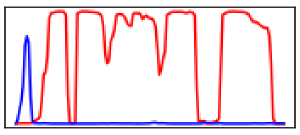 145AA	-	-

294	*Lactobacillus casei*	WP_079351764 (NZ_MWVD01000037.1)	WP_079351763 (NZ_MWVD01000037.1)	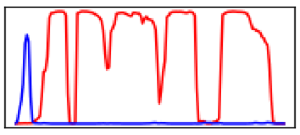 145AA	WP_003589991 (NZ_MWVD01000037.1)	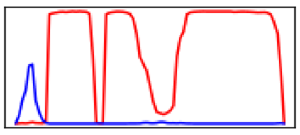 81AA
295	*Lactobacillus casei*	WP_079351732 (NZ_MWVD01000035.1)	WP_003593120 (NZ_MWVD01000035.1)	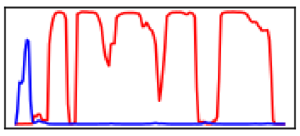 145AA	-	-
296	*Lactobacillus casei*	WP_080596974 (NZ_AUYM01000008.1)	WP_003605670 (NZ_AZCT01000027.1)	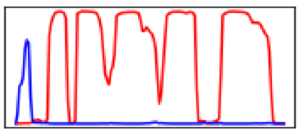 145AA	WP_003605672 (NZ_AZCT01000027.1)	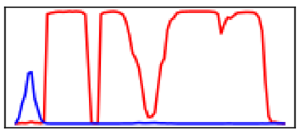 86AA
297	*Lactobacillus casei CRF28*	WP_003583440 (NZ_AFYN01000009.1)	WP_032786495 (NZ_AFYN01000009.1)	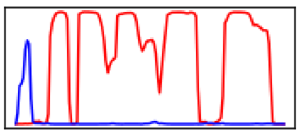 145AA	WP_003583435 (NZ_AFYN01000009.1)	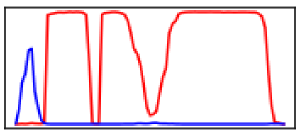 86AA
298	*Lactobacillus casei DSM 20011 = JCM 1134*	WP_011674165 (NZ_MOAG01000229.1)	WP_011674164 (NZ_MOAG01000229.1)	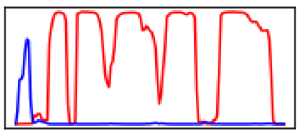 145AA	WP_003593118 (NZ_MOAG01000229.1)	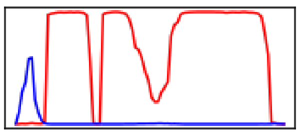 84AA
299	*Lactobacillus casei LOCK919*	WP_020751452 (NC_021721.1)	WP_020751451 (NC_021721.1)	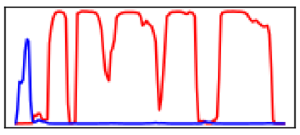 145AA	WP_020751450 (NC_021721.1)	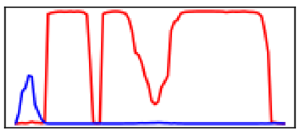 84AA
300	*Lactobacillus casei M36*	WP_003587046 (NZ_AFYO01000017.1)	WP_003587044 (NZ_AFYO01000017.1)	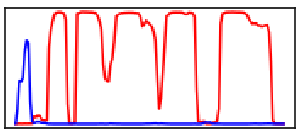 145AA	-	-
301	*Lactobacillus casei subsp. casei ATCC 393*	WP_025013808 (NZ_AP012544.1)	WP_003589992 (NZ_LOJN01000168.1)	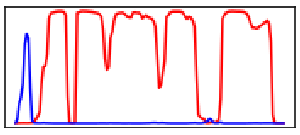 145AA	-	-
302	*Lactobacillus fermentum MTCC 8711*	WP_003570613 (NZ_JUIR01000012.1)	WP_003570610 (NZ_JUIR01000012.1)	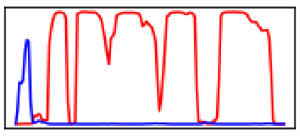 145AA	WP_016379708 (NZ_JUIR01000012.1)	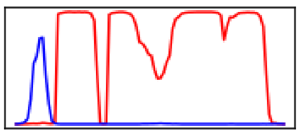 90AA
303	*Lactobacillus manihotivorans*	WP_054715260 (NZ_AZEU01000133.1)	WP_054715259 (NZ_AZEU01000133.1)	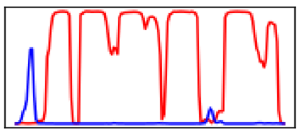 146AA	WP_056963501 (NZ_AZEU01000133.1)	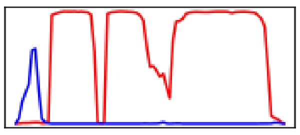 83AA
304	*Lactobacillus manihotivorans*	WP_056963710 (NZ_AZEU01000148.1)	WP_054719252 (NZ_AZEU01000148.1)	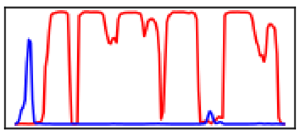 145AA	WP_054719246 (NZ_AZEU01000148.1)	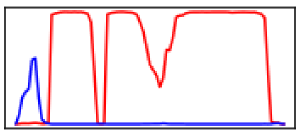 83AA
305	*Lactobacillus paracasei*	WP_086430649 (NZ_NCSN01000010.1)	WP_086430648 (NZ_NCSN01000010.1)	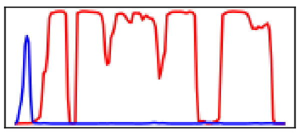 145AA	WP_086430647 (NZ_NCSN01000010.1)	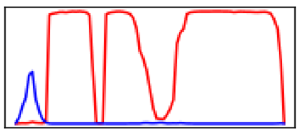 81AA
306	*Lactobacillus paracasei*	WP_076665404 (NZ_MKFY01000019.1)	WP_016382049 (NZ_MKFY01000019.1)	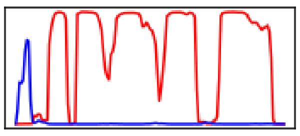 145AA	-	-
307	*Lactobacillus paracasei*	WP_081528904 (NZ_CP014988.1)	WP_016372366 (NZ_CP014988.1)	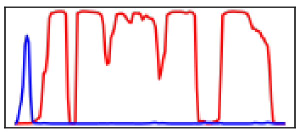 145AA	WP_016370734 (NZ_CP014988.1)	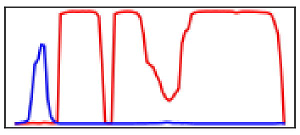 85AA

308	*Lactobacillus paracasei*	WP_086430639 (NZ_NCSN01000007.1)	WP_049173607 (NZ_NCSN01000007.1)	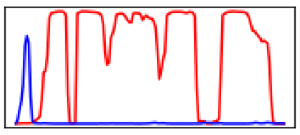 145AA	WP_032798701 (NZ_NCSN01000007.1)	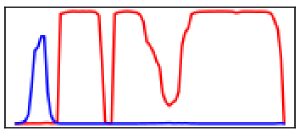 85AA
309	*Lactobacillus paracasei*	WP_085694267 (NZ_NDXH01000020.1)	WP_003572397 (NZ_NDXH01000020.1)	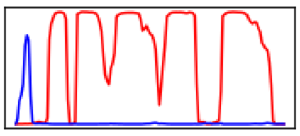 145AA	WP_003572395 (NZ_NDXH01000020.1)	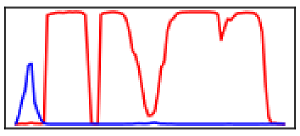 86AA
310	*Lactobacillus paracasei*	WP_081528686 (NZ_CP014985.1)	WP_081528687 (NZ_CP014985.1)	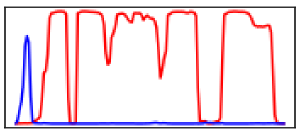 144AA	WP_081528688 (NZ_CP014985.1)	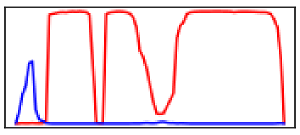 81AA
311	*Lactobacillus paracasei*	WP_071252620 (NZ_MBTZ01000069.1)	WP_016372366 (NZ_CP014988.1)	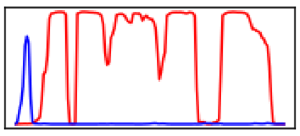 145AA	-	-
312	*Lactobacillus paracasei N1115*	WP_025376372 (NZ_CP007124.1)	WP_025376373 (NZ_CP007124.1)	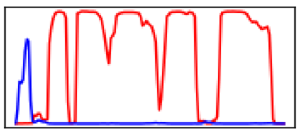 145AA	-	-
313	*Lactobacillus paracasei NRIC 0644*	WP_045627043 (NZ_BAYM01000047.1)	WP_045627045 (NZ_BAYM01000047.1)	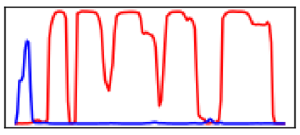 145AA	WP_019891174 (NZ_BAYM01000047.1)	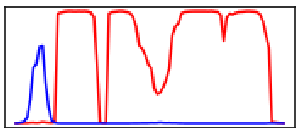 90AA
314	*Lactobacillus paracasei subsp. paracasei ATCC 25302*	WP_003663069 (NZ_AZGH01000008.1)	WP_003663067 (NZ_AZGH01000008.1)	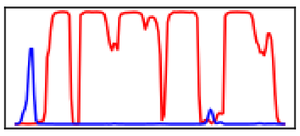 146AA	WP_003663066 (NZ_AZGH01000008.1)	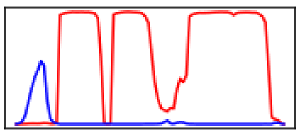 86AA
315	*Lactobacillus paracasei subsp. paracasei CNCM I-2877*	WP_019891173 (NZ_ANLE01000061.1)	WP_016381682 (NZ_ANLE01000061.1)	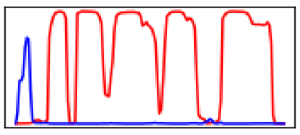 145AA	WP_019891174 (NZ_BAYM01000047.1)	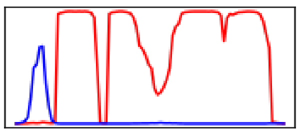 90AA
316	*Lactobacillus paracasei subsp. paracasei CNCM I-4270*	WP_016368668 (NZ_ANJX01000002.1)	WP_003607076 (NZ_KV820602.1)	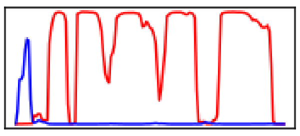 145AA	WP_003607075 (NZ_KV820602.1)	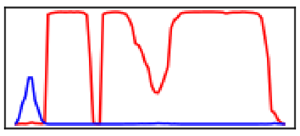 84AA
317	*Lactobacillus paracasei subsp. paracasei CNCM I-4649*	WP_016381630 (NZ_ANKH01000634.1)	WP_016381629 (NZ_ANKH01000634.1)	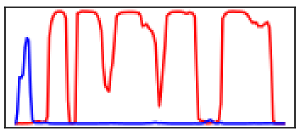 145AA	WP_003587042 (NZ_ANKH01000634.1)	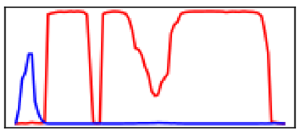 84AA
318	*Lactobacillus paracasei subsp. paracasei CNCM I-4649*	WP_016381681 (NZ_ANKH01000659.1)	WP_016381682 (NZ_ANLE01000061.1)	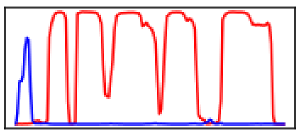 145AA	-	-
319	*Lactobacillus paracasei subsp. paracasei DSM 5622*	BAN72694 (AP012541.1)	BAN72695 (AP012541.1)	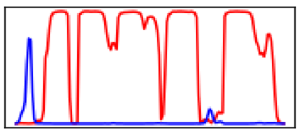 145AA	-	-
320	*Lactobacillus paracasei subsp. paracasei Lpp122*	WP_016370736 (NZ_ANKW01000035.1)	WP_016370735 (NZ_ANKA01000087.1)	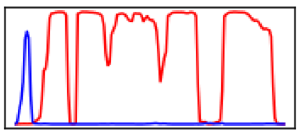 144AA	WP_016370734 (NZ_CP014988.1)	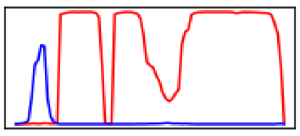 85AA
321	*Lactobacillus paracasei subsp. paracasei Lpp122*	WP_016383874 (NZ_ANKW01000069.1)	WP_003597597 (NZ_ANKW01000069.1)	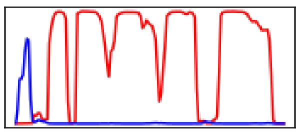 145AA	WP_016383873 (NZ_ANKW01000069.1)	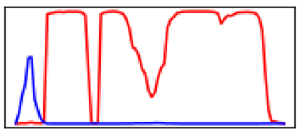 86AA

322	*Lactobacillus paracasei subsp. paracasei Lpp122*	WP_016383855 (NZ_ANKW01000065.1)	WP_003570610 (NZ_JUIR01000012.1)	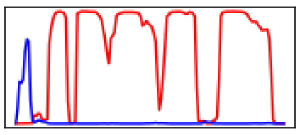 145AA	-	-
323	*Lactobacillus paracasei subsp. paracasei Lpp125*	WP_019887489 (NZ_ANKM01000049.1)	WP_019887490 (NZ_ANKM01000049.1)	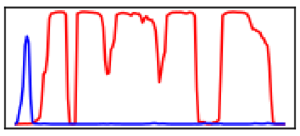 145AA	-	-
324	*Lactobacillus paracasei subsp. paracasei Lpp17*	WP_016385204 (NZ_ANMH01000013.1)	WP_016385205 (NZ_ANMH01000013.1)	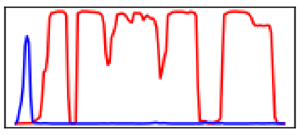 144AA	-	-
325	*Lactobacillus paracasei subsp. paracasei Lpp221*	WP_016379700 (NZ_ANKF01000143.1)	WP_016379701 (NZ_ANKF01000143.1)	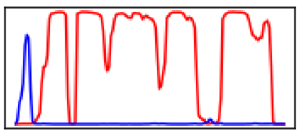 145AA	-	-
326	*Lactobacillus paracasei subsp. paracasei Lpp223*	WP_012491069 (NZ_MODS01000064.1)	WP_012491068 (NZ_MODS01000064.1)	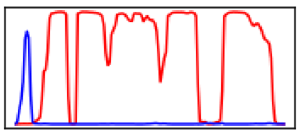 144AA	WP_012491067 (NZ_MODS01000064.1)	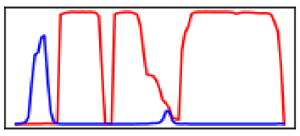 85AA
327	*Lactobacillus paracasei subsp. paracasei Lpp225*	WP_016388588 (NZ_ANMM01000028.1)	WP_016388587 (NZ_ANMM01000028.1)	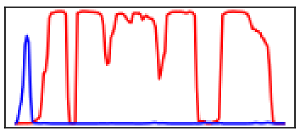 145AA	-	-
328	*Lactobacillus paracasei subsp. paracasei Lpp226*	WP_016388777 (NZ_ANMN01000037.1)	WP_032789665 (NZ_ANMN01000037.1)	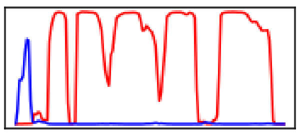 145AA	-	-
329	*Lactobacillus paracasei subsp. paracasei Lpp229*	WP_016364064 (NZ_ANJT01000068.1)	WP_016364063 (NZ_ANJT01000068.1)	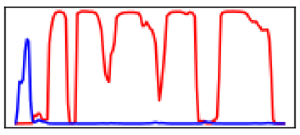 145AA	-	-
330	*Lactobacillus paracasei subsp. paracasei Lpp41*	EPC70981 (ANKE01000615.1)	EPC70980 (ANKE01000615.1)	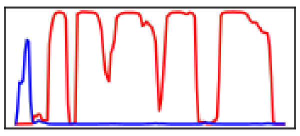 145AA	EPC70979 (ANKE01000615.1)	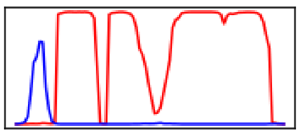 90AA
331	*Lactobacillus paracasei subsp. paracasei Lpp48*	WP_019916522 (NZ_MPOP01000034.1)	WP_016379004 (NZ_MPOP01000034.1)	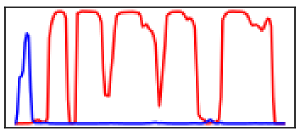 145AA	WP_019916526 (NZ_MPOP01000034.1)	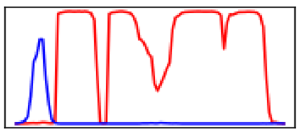 90AA
332	*Lactobacillus paracasei subsp. paracasei Lpp49*	WP_016383000 (NZ_ANKJ01000091.1)	WP_016383001 (NZ_ANKJ01000091.1)	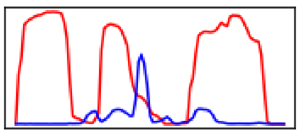 95AA	-	-
333	*Lactobacillus paracasei subsp. paracasei Lpp7*	WP_016366324 (NZ_ANJV01000379.1)	WP_016366325 (NZ_ANLF01000018.1)	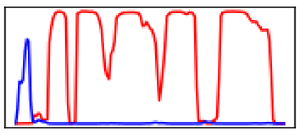 145AA	WP_016366326 (NZ_ANJV01000379.1)	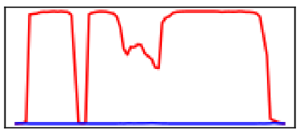 78AA
334	*Lactobacillus paracasei subsp. paracasei Lpp70*	WP_019897686 (NZ_ANLF01000018.1)	WP_016366325 (NZ_ANLF01000018.1)	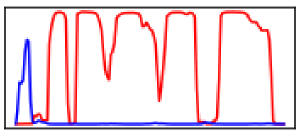 145AA	-	-
335	*Lactobacillus rhamnosus*	WP_049180014 (NZ_JUON01000169.1)	WP_049180015 (NZ_JUON01000169.1)	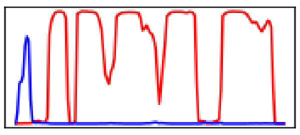 145AA	-	-

336	*Lactobacillus rhamnosus*	WP_064537301 (NZ_JTIR01000048.1)	WP_064537299 (NZ_JTIR01000048.1)	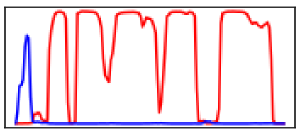 145AA	WP_064537297 (NZ_JTIR01000048.1)	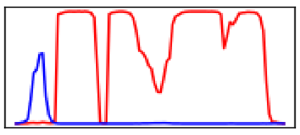 90AA
337	*Lactobacillus rhamnosus*	WP_049152495 (NZ_LPNV01000050.1)	WP_003660096 (NZ_LPNV01000050.1)	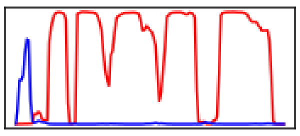 145AA	WP_016382191 (NZ_LPNV01000050.1)	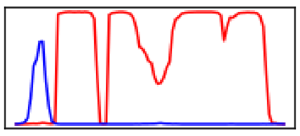 90AA
338	*Lactobacillus rhamnosus*	WP_049150937 (NZ_JVPR01000100.1)	WP_049150940 (NZ_JVIZ01000273.1)	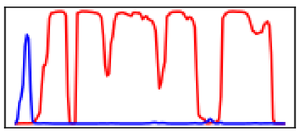 145AA	WP_049150941 (NZ_JVIZ01000273.1)	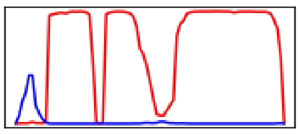 81AA
339	*Lactobacillus rhamnosus*	WP_064657561 (NZ_JTIH01000020.1)	WP_064657560 (NZ_JTIH01000020.1)	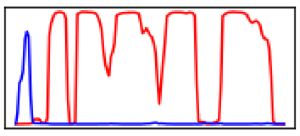 145AA	WP_019916526 (NZ_MPOP01000034.1)	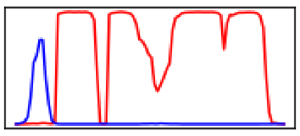 90AA
340	*Lactobacillus rhamnosus 2166*	WP_016379706 (NZ_JTIF01000226.1)	WP_016379707 (NZ_ANKF01000144.1)	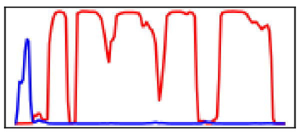 145AA	-	-
341	*Lactobacillus sharpeae JCM 1186*	WP_054679368 (NZ_AYYO01000001.1)	WP_054679365 (NZ_AYYO01000001.1)	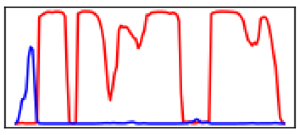 126AA	WP_054679361 (NZ_AYYO01000001.1)	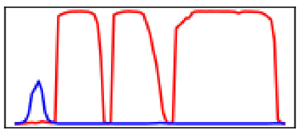 83AA
342	*Lactobacillus sp. HMSC066G01*	WP_070635826 (NZ_KV808018.1)	WP_016386252 (NZ_KV808018.1)	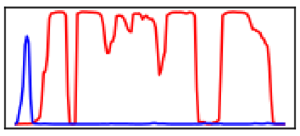 145AA	WP_064517355 (NZ_KV808018.1)	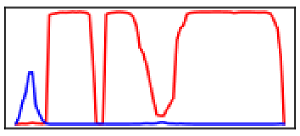 81AA
343	*Lactobacillus sp. HMSC068B07*	WP_070543897 (NZ_KV798300.1)	WP_070543898 (NZ_KV798300.1)	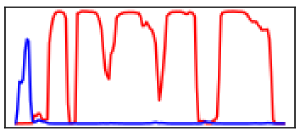 145AA	-	-
344	*Lactobacillus sp. HMSC073B09*	WP_070693973 (NZ_KV814084.1)	WP_031547246 (NZ_KV814084.1)	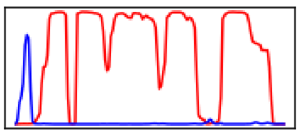 145AA	WP_003589991 (NZ_MWVD01000037.1)	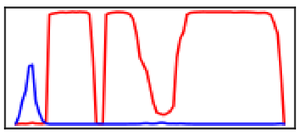 81AA
345	*Lactobacillus sp. HMSC073D04*	WP_003607077 (NZ_KV820602.1)	WP_003607076 (NZ_KV820602.1)	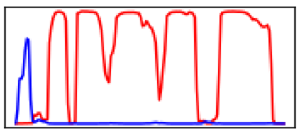 145AA	-	-
346	*Lactobacillus zeae DSM 20178 = KCTC 3804*	WP_010493216 (NZ_AZCT01000027.1)	WP_003605670 (NZ_AZCT01000027.1)	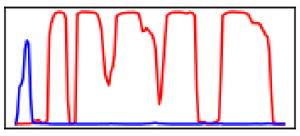 145AA	-	-
347	*Lentibacillus jeotgali*	WP_010529081 (NZ_AGAV01000003.1)	WP_010529080 (NZ_AGAV01000003.1)	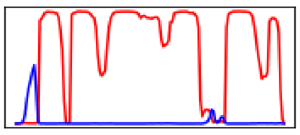 145AA	-	-
348	*Lentibacillus jeotgali*	WP_010531158 (NZ_AGAV01000014.1)	WP_010531159 (NZ_AGAV01000014.1)	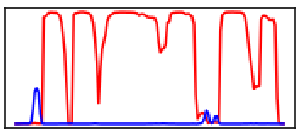 153AA	-	-
349	*Lentibacillus jeotgali*	WP_010529469 (NZ_AGAV01000004.1)	WP_010529468 (NZ_AGAV01000004.1)	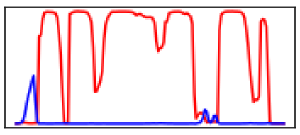 150AA	-	-

350	*Lentibacillus persicus*	SFE33741 (FOMR01000013.1)	SFE33704 (FOMR01000013.1)	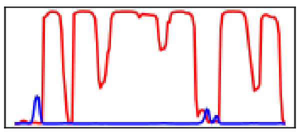 153AA	-	-
351	*Lentibacillus sp. LAM0015*	WP_068441622 (NZ_CP013862.1)	WP_068441620 (NZ_CP013862.1)	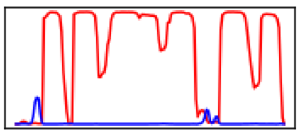 153AA	-	-
352	*Lentibacillus sp. LAM0015*	WP_068441327 (NZ_CP013862.1)	WP_068441324 (NZ_CP013862.1)	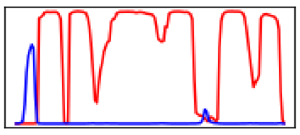 150AA	-	-
353	*Listeria grayi DSM 20601*	WP_003759306 (NZ_CP022021.1)	WP_003725255 (NZ_CP022021.1)	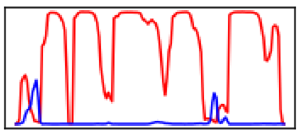 143AA	-	-
354	*Listeria monocytogenes*	WP_061385572 (NZ_LUEM01000011.1)	WP_061385573 (NZ_LUEM01000011.1)	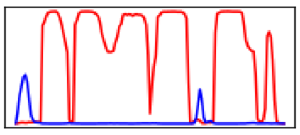 141AA	WP_061385574 (NZ_LUEM01000011.1)	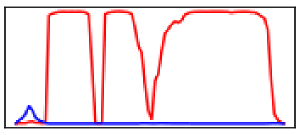 82AA
355	*Listeria monocytogenes*	WP_070263476 (NZ_MKOY01000014.1)	WP_070263475 (NZ_MKOY01000014.1)	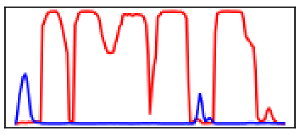 141AA	WP_003740246 (NZ_MKOY01000014.1)	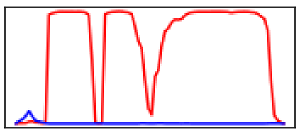 82AA
356	*Listeria monocytogenes*	WP_031659845 (NZ_MDOG01000002.1)	WP_031659847 (NZ_MDOG01000002.1)	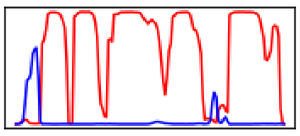 143AA	-	-
357	*Listeria monocytogenes FSL J1-208*	WP_003740248 (NZ_CM001470.1)	WP_003740247 (NZ_CM001470.1)	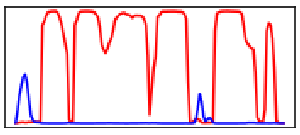 141AA	-	-
358	*Listeria riparia*	WP_052008952 (NZ_AODL01000031.1)	WP_036101884 (NZ_AODL01000031.1)	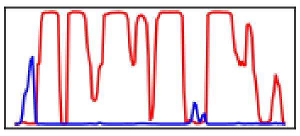 159AA	-	-
359	*Listeriaceae bacterium FSL M6-0635*	WP_036091936 (NZ_JNFB01000013.1)	WP_036091940 (NZ_JNFB01000013.1)	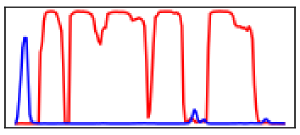 153AA	WP_036091950 (NZ_JNFB01000013.1)	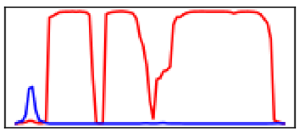 81AA
360	*Lysinibacillus fusiformis ZC1*	WP_004233231 (NZ_ADJR01000099.1)	WP_004233228 (NZ_ADJR01000099.1)	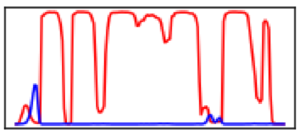 156AA	-	-
361	*Lysinibacillus sp. A1*	WP_036124811 (NZ_CP010820.1)	WP_052323572 (NZ_CP010820.1)	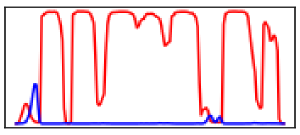 156AA	-	-
362	*Lysinibacillus sp. AR18-8*	WP_066036877 (NZ_MDGU01000006.1)	WP_066036878 (NZ_MDGU01000006.1)	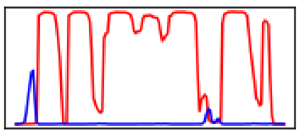 153AA	-	-
363	*Lysinibacillus sp. BF-4*	WP_036142074 (NZ_JPUW01000001.1)	WP_036142072 (NZ_JPUW01000001.1)	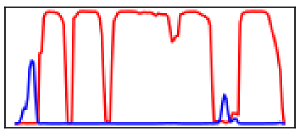 135AA	-	-

364	*Lysinibacillus sp. TC-13*	SCY83743 (FMVP01000028.1)	SCY83734 (FMVP01000028.1)	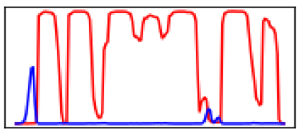 153AA	-	-
365	*Lysinibacillus sphaericus*	WP_036165803 (NZ_JPDL01000082.1)	WP_051891007 (NZ_JPDL01000082.1)	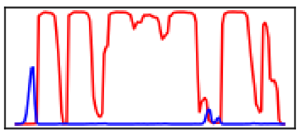 153AA	-	-
366	*Lysinibacillus sphaericus*	WP_031417611 (NZ_JPDK01000103.1)	WP_051889595 (NZ_JPDK01000103.1)	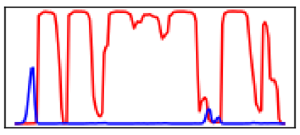 153AA	-	-
367	*Marinococcus halophilus*	WP_079473990 (NZ_FVZD01000003.1)	WP_079473991 (NZ_FVZD01000003.1)	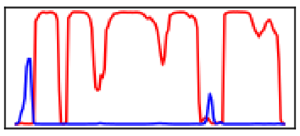 149AA	-	-
368	*Marinococcus halotolerans*	WP_022795075 (NZ_ATVM01000031.1)	WP_022795074 (NZ_ATVM01000031.1)	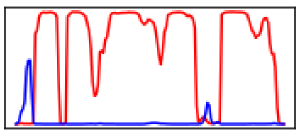 151AA	-	-
369	*Natribacillus halophilus*	SDI43370 (FNEN01000002.1)	SDI43350 (FNEN01000002.1)	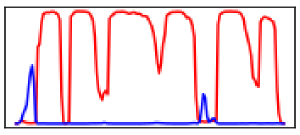 147AA	-	-
370	*Oceanobacillus massiliensis*	WP_010652039 (NZ_HE610981.1)	WP_010652040 (NZ_HE610981.1)	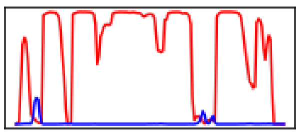 156AA	-	-
371	*Oceanobacillus picturae*	WP_058950451 (NZ_BBXV01000027.1)	WP_082667709 (NZ_BBXV01000027.1)	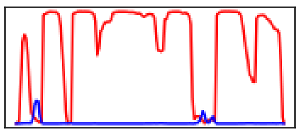 156AA	-	-
372	*Oceanobacillus sp. Marseille-P3532*	WP_080871717 (NZ_LT800496.1)	WP_080871718 (NZ_LT800496.1)	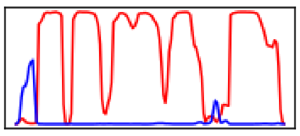 141AA	-	-
373	*Paraliobacillus ryukyuensis*	WP_079708040 (NZ_FVZO01000014.1)	WP_079708041 (NZ_FVZO01000014.1)	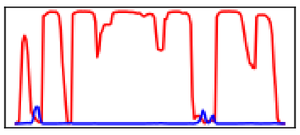 156AA	-	-
374	*Planococcus sp. L10.15*	WP_083553759 (NZ_CP016541.2)	WP_049694968 (NZ_CP016541.2)	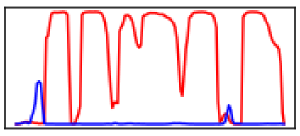 151AA	-	-
375	*Salimicrobium sp. MJ3*	WP_008590576 (NZ_CP011361.2)	WP_008590574 (NZ_CP011361.2)	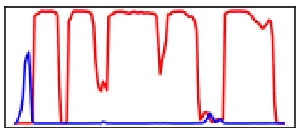 142AA	-	-
376	*Streptococcus pneumoniae*	CRG01734 (CVLS01000410.1)	CRG01733 (CVLS01000410.1)	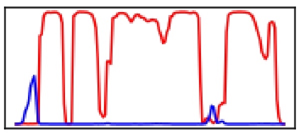 145AA	-	-
377	*Thalassobacillus devorans*	WP_028784619 (NZ_KI543238.1)	WP_028784620 (NZ_KI543238.1)	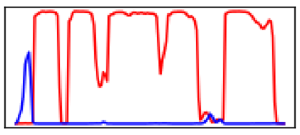 142AA	-	-

378	*Thalassobacillus sp. C254*	WP_054637624 (NZ_BAXQ01000033.1)	WP_054637625 (NZ_BAXQ01000033.1)	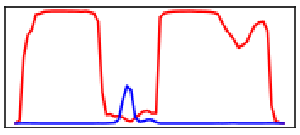 66AA	-	-
379	*Thalassobacillus sp. TM-1*	WP_062440437 (NZ_CTEA01000004.1)	WP_062440434 (NZ_CTEA01000004.1)	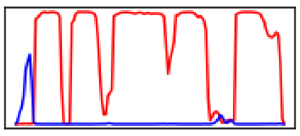 135AA	-	-
380	*Virgibacillus dokdonensis*	WP_077706711 (NZ_LT745763.1)	WP_077706712 (NZ_LT745763.1)	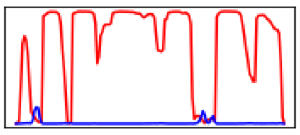 156AA	-	-
381	*Virgibacillus halodenitrificans*	WP_077356148 (NZ_FUHR01000003.1)	WP_077356150 (NZ_FUHR01000003.1)	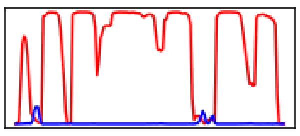 156AA	-	-
382	*Virgibacillus sp. LM2416*	WP_089060631 (NZ_CP022315.1)	WP_089060632 (NZ_CP022315.1)	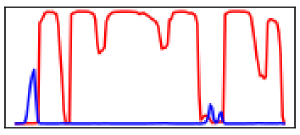 146AA	-	-
383	*Virgibacillus sp. LM2416*	WP_089060877 (NZ_CP022315.1)	WP_089060878 (NZ_CP022315.1)	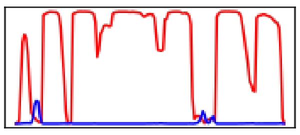 156AA	-	-
384	*Virgibacillus sp. Marseille-P3469*	WP_088051883 (NZ_LT732554.1)	WP_088051882 (NZ_LT732554.1)	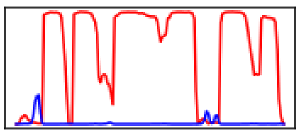 153AA	-	-
385	*uncultured Clostridium sp.*	SCJ53531 (FMGS01000017.1)	SCJ53548 (FMGS01000017.1)	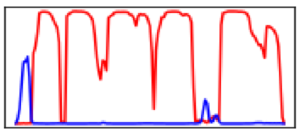 137AA	SCJ53562 (FMGS01000017.1)	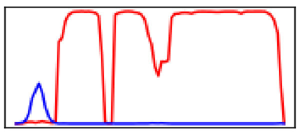 82AA
386	*uncultured Clostridium sp.*	SCJ44736 (FMGS01000008.1)	SCJ44746 (FMGS01000008.1)	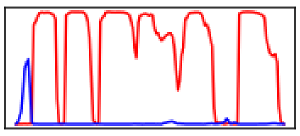 150AA	-	-
387	*uncultured Lachnospira sp.*	SCJ25282 (FMGY01000006.1)	SCJ25252 (FMGY01000006.1)	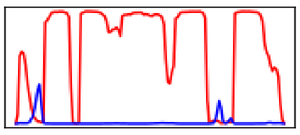 137AA	-	-

* For each hit the protein accession number is given followed by the corresponding DNA identifier (in brackets), and the size of the protein (Nº residues).

** secondary structure was predicted using the algorithms available at webpage http://raptorx.uchicago.edu/StructurePropertyPred/predict/. In each plot the probability to form a β-strand (blue line) or an α-helix (red line) (y-axes) is given as a function of the protein position. Independent of the protein size, a fixed X-axes width was used for plotting the primary protein sequence positions. Note that three of the 87 putative Aux1$_{LS20}$ (3.4%) and 24 of the 387 putative Aux2$_{LS20}$ (6.2%) homologs appear to contain a structural element 5′ of the Ribbon-Helix-Helix signature.
