# Supplementary material for: The Bacillus subtilis Conjugative Plasmid pLS20 Encodes Two Ribbon-Helix-Helix Type Auxiliary Relaxosome Proteins That Are Essential for Conjugation
Source: Front Microbiol. 2017 Nov 3;8:2138. doi: 10.3389/fmicb.2017.02138 (PMC5675868; doi:10.3389/fmicb.2017.02138)
Supplement: Supplementary file 5 [file Image_1.PDF]

Supplemental Figure 1

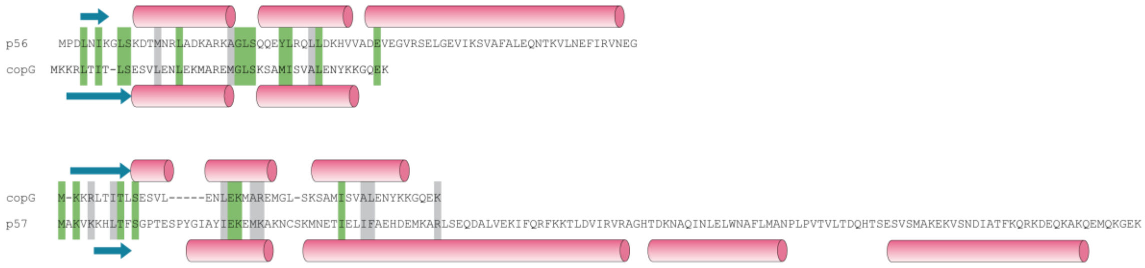

Figure S1. pLS20cat proteins p56 and p57 are predicted to contain a Ribbon-Helix-Helix (RHH) DNA binding motif in their N-terminal region. Primary sequences of the deduced protein sequences of pLS20cat genes *56* and *57* are aligned with the classical RHH CopG protein encoded by plasmid pMV158 (Gomis-Ruth et al., 1998). The positions of the (predicted) ribbon and helical structures are presented by blue arrows and pink cylinders, respectively. Identical and conserved residues are indicated against a green and grey background, respectively.

Gomis-Ruth, F. X., Sola, M., Acebo, P., Parraga, A., Guasch, A., Eritja, R. et al. (1998). The structure of plasmid-encoded transcriptional repressor CopG unliganded and bound to its operator. *EMBO J.* 17, 7404-7415.
