# Supplementary material for: The Bacillus subtilis Conjugative Plasmid pLS20 Encodes Two Ribbon-Helix-Helix Type Auxiliary Relaxosome Proteins That Are Essential for Conjugation
Source: Front Microbiol. 2017 Nov 3;8:2138. doi: 10.3389/fmicb.2017.02138 (PMC5675868; doi:10.3389/fmicb.2017.02138)
Supplement: Supplementary file 6 [file Image_2.PDF]

Supplemental Figure 2

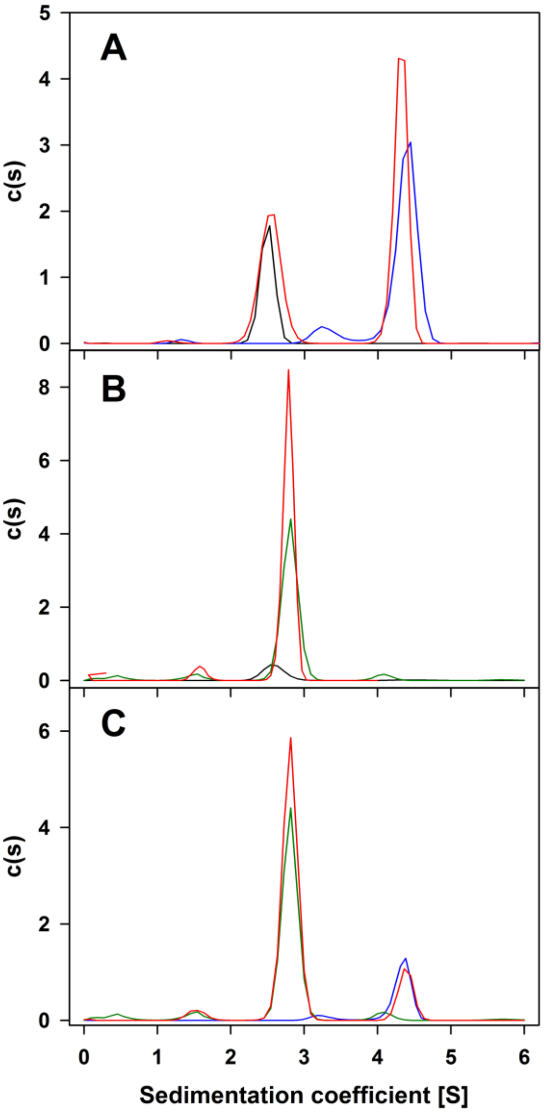

Figure S2. The pLS20cat relaxosome proteins do not interact in solution. Purified proteins in solution at 12 µM were studied by sedimentation velocity. Graphics show the sedimentation coefficient distribution c(s) profiles of samples containing separately or both of the following two proteins. (**A**) Aux1$_{LS20}$ (black line), Aux2$_{LS20}$ (blue line) and both (red line); (**B**) Aux1$_{LS20}$ (black line), Rel$_{LS20}$ (green line) and both (red line); (**C**) Aux2$_{LS20}$ (blue line), Rel$_{LS20}$ (green line) and both (red line).
