# Supplementary material for: The Bacillus subtilis Conjugative Plasmid pLS20 Encodes Two Ribbon-Helix-Helix Type Auxiliary Relaxosome Proteins That Are Essential for Conjugation
Source: Front Microbiol. 2017 Nov 3;8:2138. doi: 10.3389/fmicb.2017.02138 (PMC5675868; doi:10.3389/fmicb.2017.02138)
Supplement: Supplementary file 7 [file Image_3.PDF]

Supplemental Figure 3

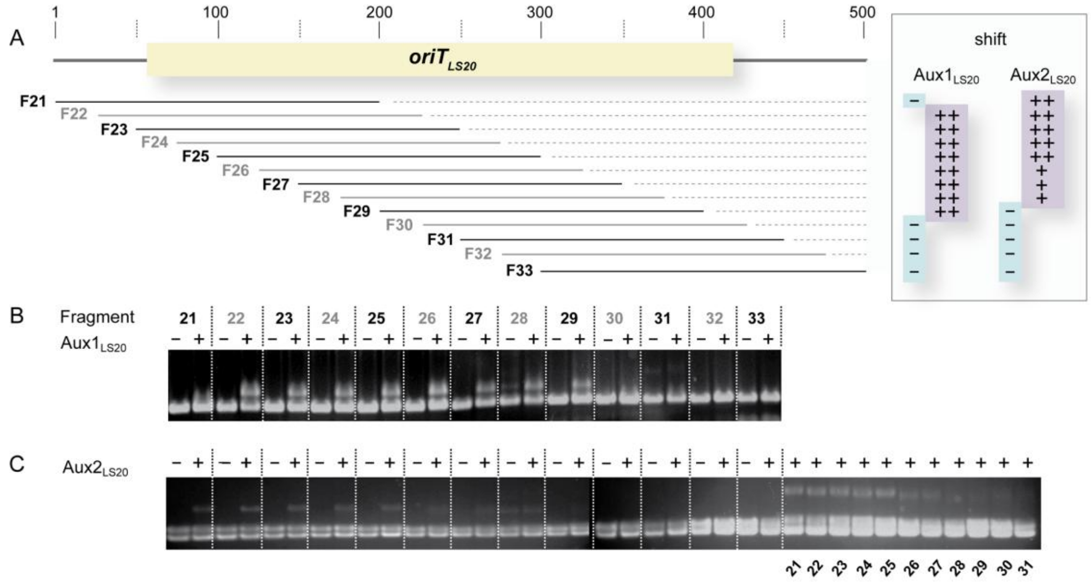

Figure S3. Delineation of the binding sites of Aux1$_{LS20}$ and Aux2$_{LS20}$ in *oriT$_{LS20}$*. (A). Schematic presentation of the different DNA fragments F21-F33 used in EMSAs. The 362 bp *oriT$_{LS20}$* region is presented as a black bar on the top of the Figure. The grey region summarizes the results of the EMSAs obtained for Aux1$_{LS20}$ and Aux2$_{LS20}$, which are presented in (**B**) and (**C**), respectively. Each lane contained 90 nM of protein together with 170 ng of the indicated DNA fragment. In the case of Aux2$_{LS20}$ each lane also contained an additional control DNA fragment of 176 bp to observe possible unspecific binding (220 ng, corresponding to pLS20cat positions 63,774-63,950 of accession number NC_015148.1). To illustrate better the difference in binding of Aux2$_{LS20}$ to fragments F21-F25 compared to fragments F26-F28, these samples are loaded in duplicate at the right part of Figure (**C**). DNA was loaded with/out protein as indicated.
